# Identification of novel Y chromosome encoded transcripts by testis transcriptome analysis of mice with deletions of the Y chromosome long arm

**DOI:** 10.1186/gb-2005-6-12-r102

**Published:** 2005-12-02

**Authors:** Aminata Touré, Emily J Clemente, Peter Ellis, Shantha K Mahadevaiah, Obah A Ojarikre, Penny AF Ball, Louise Reynard, Kate L Loveland, Paul S Burgoyne, Nabeel A Affara

**Affiliations:** 1 Division of Developmental Genetics, MRC National Institute for Medical Research, Mill Hill, London, NW7 1AA, UK; 2 University of Cambridge, Department of Pathology, Tennis Court Road, Cambridge, CB2 1QP, UK; 3 Monash Institute of Medical Research, Monash University, and The Australian Research Council Centre of Excellence in Biotechnology and Development, Melbourne, Victoria 3168 Australia

## Abstract

**Background:**

The male-specific region of the mouse Y chromosome long arm (MSYq) is comprised largely of repeated DNA, including multiple copies of the spermatid-expressed *Ssty *gene family. Large deletions of MSYq are associated with sperm head defects for which *Ssty *deficiency has been presumed to be responsible.

**Results:**

In a search for further candidate genes associated with these defects we analyzed changes in the testis transcriptome resulting from MSYq deletions, using testis cDNA microarrays. This approach, aided by accumulating mouse MSYq sequence information, identified transcripts derived from two further spermatid-expressed multicopy MSYq gene families; like *Ssty*, each of these new MSYq gene families has multicopy relatives on the X chromosome. The *Sly *family encodes a protein with homology to the chromatin-associated proteins XLR and XMR that are encoded by the X chromosomal relatives. The second MSYq gene family was identified because the transcripts hybridized to a microarrayed X chromosome-encoded testis cDNA. The X loci ('*Astx*') encoding this cDNA had 92-94% sequence identity to over 100 putative Y loci ('*Asty*') across exons and introns; only low level *Asty *transcription was detected. More strongly transcribed recombinant loci were identified that included *Asty *exons 2-4 preceded by *Ssty1 *exons 1, 2 and part of exon 3. Transcription from the *Ssty1 *promotor generated spermatid-specific transcripts that, in addition to the variable inclusion of *Ssty1 *and *Asty *exons, included additional exons because of the serendipitous presence of splice sites further downstream.

**Conclusion:**

We identified further MSYq-encoded transcripts expressed in spermatids and deriving from multicopy Y genes, deficiency of which may underlie the defects in sperm development associated with MSYq deletions.

## Abstract 2

Microarray analysis of the changes in the testis transcriptome resulting from deletions of the male-specific region on the mouse chromosome long arm (MSYq) identified novel Y chromosome-encoded transcripts.

## Background

The mammalian Y chromosome seems predisposed to accumulating multiple copies of genes that play a role in spermatogenesis [1-7]. Determining the precise functions of such multicopy genes is inherently difficult. In humans and mouse, indications as to function have so far derived from the analysis of naturally occurring deletion mutants. In the mouse, deletions in MSYq (the Y chromosome long arm, excluding the pseudo-autosomal region) affect sperm development (spermiogenesis) and function, with the severity of the sperm defects being correlated with the extent of the deletion [3,8-14]. Mouse MSYq appears to be composed predominantly of highly repeated DNA sequences [15-22], and when the present project was initiated the only known MSYq encoded testis transcripts derived from the complex multicopy *Ssty *gene family [3,23-25]. This gene family, with two distinct subfamilies, namely *Ssty1 *and *Ssty2*, is expressed in the testis during spermiogenesis and, in the absence of other candidates, it had been postulated that *Ssty *deficiency is responsible for the defective sperm development in MSYq deficient mice.

In this study we utilized custom-made testis cDNA microarrays to identify further Y encoded testis transcripts that are absent or reduced in level as a consequence of MSYq deficiencies.

## Results

### Identifying MSYq encoded testis transcripts by microarray analysis

**Figure 1:**
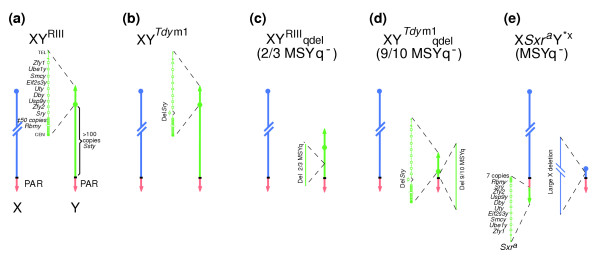
Sex chromosome complements of the mice with MSYq deficiencies and relevant control mice. **(a) **XY^RIII ^control, illustrating the previously documented male specific gene content of the mouse Y chromosome. The short arm (shown expanded) carries seven single copy genes, one duplicated gene (*Zfy*), and multiple copies of *Rbmy*. MSYq carries multiple copies of the *Ssty *gene family. **(b) **XY^*Tdym*1 ^control. This male has a normal Y gene complement except that a 11 kb deletion has removed the testis determinant *Sry*; the *Sry *deletion is complemented by an *Sry *transgene located on an autosome. **(c) **The variant Y^RIII^qdel has a deletion removing about two-thirds of MSYq. **(d) **The variant Y^*Tdym*1^qdel has a large deletion removing about nine-tenths of MSYq, together with the small 11 kb deletion removing *Sry *(complemented by an *Sry *transgene). **(e) **X*Sxr*^*a*^Y* ^X ^mice are male because of the presence of the Y^RIII ^short arm derived, sex reversal factor *Sxr*^*a *^attached distal to the X pseudo-autosomal region (PAR). *Sxr*^*a *^comprises most of the Y short arm except for a substantial reduction in copies of *Rbmy*. The Y* ^X ^chromosome is in effect an X chromosome with a deletion from just proximal to *Amel *(close to the X PAR boundary) to within the *DXHXF34 *sequence cluster adjacent to the X centromere. It provides a second PAR, which is essential in order to avoid meiotic arrest. CEN, centromere; kb, kilobase; TEL, telomere.

Three MSYq-deficient mouse models were used in this study (Figure 1): XY^RIII^qdel, with deletion of about two-thirds of MSYq; XY^*Tdym*1^qdel*Sry*, with deletion of about nine tenths of MSYq; and X*Sxr*^*a*^Y* ^X^, in which the only Y specific sequences are provided by the Y short arm derived sex-reversal factor *Sxr*^*a*^, and thus lack all of MSYq. These models are hereafter abbreviated as 2/3MSYq^-^, 9/10MSYq^- ^and MSYq^-^.

**Figure 2:**
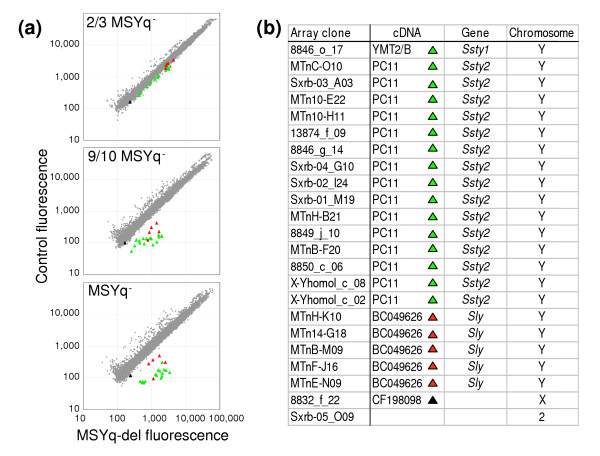
Microarray analysis of the testis transcriptomes of the three MSYq deficient models. **(a) **Scatter plots showing transcription levels for the testis transcripts of MSYq deficient models relative to their controls. Expression in the MSYq deficient mice (y-axis, Cy3 label, arbitrary units) for each clone is plotted versus expression in age- and strain-matched normal testis control (x-axis, Cy5 label, arbitrary units). Data from four technical replicates are combined. Data are normalized on the median signal for each channel and then filtered (as described in Materials and methods) to show only clones with data for two technical replicates from each model. The data points showing significant reduction are plotted as enlarged triangles (Y clones green or red, and one X clone black - for clone identities see b). **(b) **The 23 cDNA clones identifying transcripts that were significantly reduced in one or more of the MSYq deficient models. The clones deriving from the two Y families and the one X family are color coded.

To analyze the testis transcriptomes of these MSYq deficient mice we used microarrayed testis cDNA clones enriched for cDNAs deriving from spermatogenic cells (see Materials and methods, below). Total testis RNA from each of the MSYq-deficient mice (labeled with the fluorochrome Cy3) and from matched controls (labeled with Cy5) was hybridized to the array; four technical replicates were obtained for each model. The initial raw data were filtered to select only clones for which fluorescence intensity data were available from at least two of the technical replicates for each model. After this filtering, 14,681 clones remained from the complete set. As expected, scatter plots of these filtered data show that expression of the vast majority of clones is unchanged between the MSYq deficient and control mice (Figure 2a).

For transcripts encoded by single or multiple copy Y genes mapping to MSYq, substantially reduced levels should occur in one or more of the deletion models. In order to focus on potential MSYq encoded transcripts we restricted our analysis to clones exhibiting a twofold or greater reduction in fluorescence intensity relative to control and a *t* test probability of under 1% for the comparison between the replicates for the Cy3 and Cy5 fluorescence intensities. Twenty-three clones were identified as substantially reduced by these criteria in one or more of the MSYq deletion models. BLAST (basic local alignment search tool) comparisons were used to identify matching cDNAs (if previously identified) and/or the chromosomal locations of the encoding sequences. Sixteen of the 23 proved to be *Ssty *cDNA clones, thus demonstrating the efficacy of the strategy. Of the remaining seven clones, five proved to be Y encoded *Sly *cDNAs (see below), one was X encoded and one was autosomally encoded (Figure 2a, b).

### *Sly *transcription is reduced in proportion to the extent of MSYq deficiency

All five of the additional Y encoded clones were found to have homology to regions of the cDNA clone BC049626 previously identified in a large-scale cDNA sequencing project [26] (Additional data file 1). This cDNA clone initially had no chromosomal assignment, but it was subsequently ascribed to a gene, *Sly*, that maps to MSYq (MGI:2687328; Mouse Chromosome Y Mapping Project [Jessica E Alfoldi, Helen Skaletsky, Steve Rozen and David C Page at the Whitehead Institute for Biomedical Research, Cambridge, MA, USA, and the Washington University Genome Sequencing Center, St. Louis, MO, USA]). *Sly *is a member of a multicopy family, and in December 2004 a total of 65 *Sly *family members were predicted based on the Y sequence data. There is a related multicopy X gene family that includes *Xmr *and *Xlr *[27,28].

**Figure 3:**
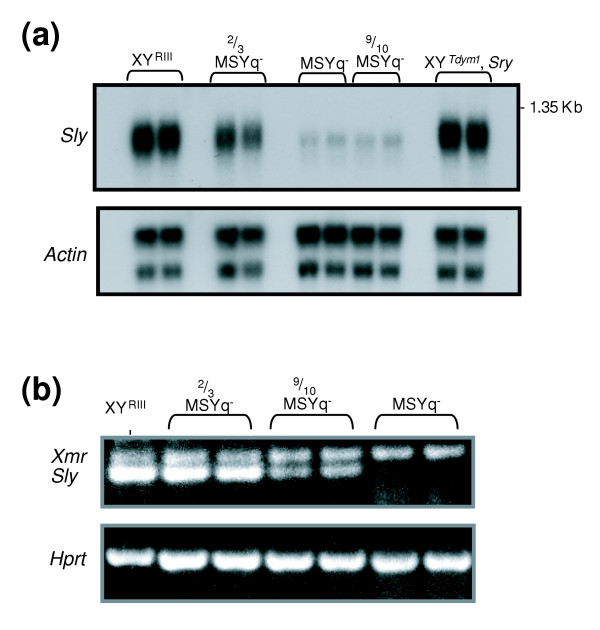
Transcription of *Sly *is reduced or absent in the MSYq deficient males. **(a) **Northern blot of total testis RNA probed with the microarrayed *Sly *cDNA clone MTnH-K10 and with an actin probe as a loading control. Hybridization to the *Sly *probe is clearly reduced in 2/3MSYq^- ^males and is further markedly reduced in the two models with more severe MSYq deficiency. **(b) **Reverse transcriptase polymerase chain reaction analysis of testis cDNA with primers that distinguish between *Sly *and *Xmr *transcripts and with *Hprt *primers as an amplification control. Some *Sly *transcripts are retained in 9/10MSYq^- ^males but they are absent in MSYq^- ^males.

To confirm the downregulation of the *Sly *transcript in the MSYq deletion mice, we probed a northern blot of total testis RNA from the three MSYq deficient models and their controls with microarray clone MTnH-K10 (Figure 3a) and subsequently with the full BC049626 cDNA (not shown). The hybridization with both probes revealed high transcript levels in the control testes, a clear reduction in 2/3MSYq^- ^testes, and further marked reductions in 9/10MSYq^- ^and MSYq^- ^testes. The reductions as estimated by phosphorimager analysis with clone MTnH-K10 (with the microarray estimates in brackets) were as follows: 2/3MSYq^-^, 47% (37%); 9/10MSYq^-^, 81% (84%); and MSYq^-^, 83% (91%). Because *Sly *has substantial homology to *Xmr*, which is also strongly transcribed in the testis, we considered that the remaining hybridization in the two models with severe MSYq deficiency could be due to cross-hybridization with *Xmr *transcripts. We therefore designed primers for reverse transcriptase polymerase chain reaction (RT-PCR) that distinguished between the two transcripts and used these to amplify template from testis cDNAs derived from the three MSYq deficient models and controls (Figure 3b). A 266 base-pair (bp) product was amplified from all samples, and sequencing confirmed that this derived from *Xmr*. A 227 bp product was also amplified from the control, 2/3MSYq^- ^and 9/10MSYq^- ^testes (although clearly reduced), but there was no amplified product from MSYq^- ^testes. Sequencing of this 227 bp product from 2/3MSYq^- ^and 9/10MSYq^- ^testes confirmed that it derived from *Sly*. Thus, only MSYq^- ^completely lacks *Sly *transcripts.

### Identification of another family of Y encoded testis transcripts reduced in MSYq deficient mice

In December 2004, a BLAST analysis of the array clone 8832_f_22 against the mouse genome registered 41 hits on the mouse X chromosome and 710 hits on the mouse Y chromosome. The arrayed cDNA clone was apparently X encoded, there being eight putative loci (for example, gi:4640881, 3118-6344) with four exons that would encode matching cDNAs; the remaining nine hits were from incomplete loci or short sequence fragments. To investigate the coding potential of the Y sequences identified in the initial analysis, a further BLAST analysis was carried out with a complete X locus, and this identified 123 putative Y chromosomal loci (for example, gi:33667254, 73667-76894) that retain the same intron/exon structure as the X loci, and with 92-94% sequence identity across exons and introns.

**Figure 4:**
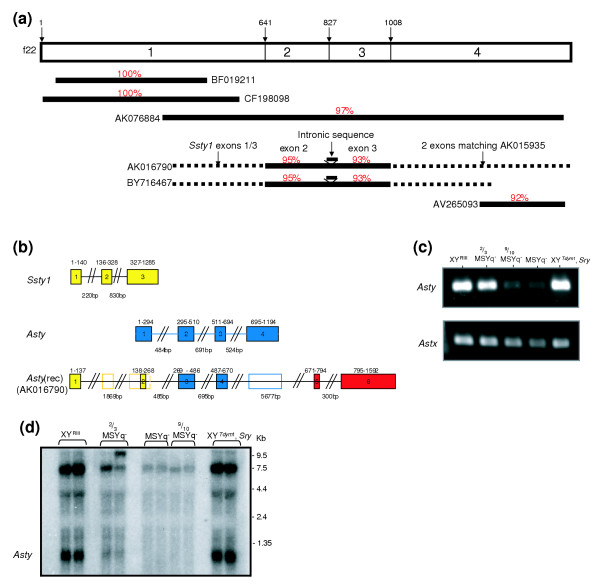
Identification of the novel MSYq encoded transcripts *Asty *and *Asty*(rec). **(a) **Testis transcripts identified from a BLAST (basic local alignment search tool) with the arrayed X encoded *Astx *cDNA clone 8832_f_22. BF019211, CF198098 and AK076884 are X encoded transcripts, whereas the rest are Y encoded transcripts. The Y encoded transcripts AK016790 and BY716467 include exons that do not derive from *Asty *(including two exons matching *Ssty1*). We refer to these transcripts as *Asty*(rec) because they derive from novel 'recombinant' loci on MSYq. For all the transcripts the percentage sequence identity is given for those regions that match the microarrayed clone. **(b) **The structure of the *Asty*(rec) locus encoding AK016790. The exons included in AK016790 are indicated by filled color coded rectangles and the inter-exonic distances are given in base pairs. The position of other *Ssty1 *and *Asty *exons not included in the AK016790 transcript are also indicated. (Note that the *Ssty1 *exon 3 is truncated in the *Asty*(rec) locus.) The two exons colored red are those that match the previously described transcript AK015935. **(c) **Reverse transcriptase polymerase chain reaction of testis cDNA with primers designed to specifically amplify *Asty*/*Asty*(rec) or *Astx *transcripts. It is clear that it is the *Asty*/*Asty*(rec) transcripts that are preferentially reduced in the MSYq deficient males. **(d) **Northern blot of total testis RNA probed with an *Asty *exon 4 probe and with an actin probe as a loading control. Transcripts ranging in size from about 1 kilobase (kb) to more than 9.5 kb are detected with the *Asty *exon 4 probe; the approximately 7.5 kb and larger transcripts definitely derive from the *Asty*/*Asty*(rec) loci (see text) and (in contrast to AK016790) must include *Asty *exon 4.

The arrayed X encoded cDNA clone shared homology with three previously identified testis cDNA clones - two apparently X encoded (CF198098, AK076884) and one Y encoded (AK016790) - and a BLAST of mouse expressed sequence tags (ESTs) identified further related testis transcripts together with others derived from small intestine, aorta and eight cell embryos. The testis cDNAs and ESTs are summarized in Figure 4a. The arrayed X encoded cDNA, together with the two exactly matching transcripts BF019211 and CF198098, derive from purified spermatocyte cDNA libraries [29]. Of the Y encoded testis transcripts, EST AV265093 matches only a short stretch of exon 4 and the two remaining transcripts, namely AK016790 and BY716467, show only segments of homology to the arrayed clone. Nevertheless, amplification from testis cDNA with an *Asty *exon 1/exon 4 primer pair generated products of 437 bp and 622 bp, and sequencing confirmed that these derived from Y chromosomal loci, the smaller product lacking exon 3. We refer to the similar X and Y loci encoding these X and Y transcripts as *Astx *and *Asty *(Amplified spermatogenic transcripts X encoded/Y encoded), respectively (for *Astx *and *Asty *transcript sequences, see Additional data file 2).

Intriguingly, the Y encoded transcripts AK016790 and BY716467, in addition to the sequence matching *Astx*/*Asty *exons 2 and 3 (and part of the intervening intron), proved to have exonic sequence matching *Ssty1 *exon 1 and part of exon 3, together with further sequence matching another testis cDNA AK015935 (Figure 4a). BLAST searches identified 'recombinant' Y genomic loci (comprising partial *Ssty1 *and *Asty *loci followed by sequence that includes exons matching AK015935) that could encode these transcripts (Figure 4b, Additional data file 3). We refer to these loci as *Asty*(rec).

The close homology between *Astx *and *Asty *suggested that the arrayed *Astx *cDNA clone would cross-hybridize with *Asty *and *Asty*(rec) RNAs. We designed primers from *Astx*/*Asty *exon 4 for RT-PCR that we thought should specifically amplify either *Astx *or *Asty*, but further analysis (see below) identified *Asty*(rec) transcripts that also include exon 4. RT-PCR analysis using these primers showed that *Asty *and/or *Asty*(rec), rather than *Astx *transcripts, are reduced in MSYq deficient mice (Figure 4c). Probing the northern blot of total testis RNA from the three MSYq deficient models and their controls with an *Asty *exon 4 probe revealed a transcript of about 1 kilobase (kb), together with other larger transcripts with sizes ranging up to more than 9.5 kb. All bands exhibited progressive reduction in intensity with increasing MSYq deficiency (Figure 4d), and thus we are confident that it is not due to cross-hybridization to *Astx*. The size for the two transcripts identified by RT-PCR is of course unknown. However, given that the microarrayed *Astx *cDNA is 1.5 kb, it is reasonable to assume that the two *Asty *transcripts should be approximately 1.3 kb (lacking exon 3) and 1.5 kb; faint bands approximating these sizes are present. In further attempts to determine the origins of these multiple sized transcripts we probed the blot with a probe matching part of exon 6 of the *Asty*(rec) transcript AK016790 (Figure 4b) and found that the bands of about 7.5 kb and above hybridized to this probe (not shown). Thus, there are transcripts derived from the 'recombinant' loci that also include *Asty *exon 4. Because the recombinant loci lack *Asty *exon 1 we then probed the blot with an *Asty *probe from exon 1, but no convincing hybridization was obtained. We conclude that the transcripts detected by the exon 4 probe that dose with the extent of the MSYq deletions derive predominantly from the *Asty*(rec) loci.

### Multiple copies of *Sly *and *Asty *are present on MSYq

**Figure 5:**
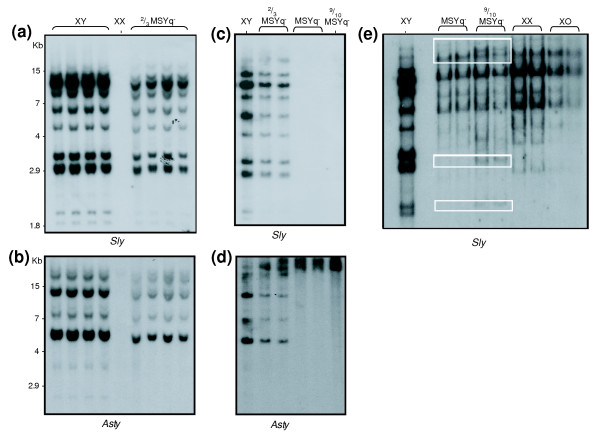
Multiple copies of *Sly *and *Asty*/*Asty*(rec) map to MSYq. **(a, b) **Southern of *Eco*RI digested DNAs showing hybridization of *Sly *probe MTnH-K10 and *Asty *exon 4 probe, respectively, to multiple male specific bands, with reduced hybridization to all bands in 2/3MSYq^- ^males. **(c, d) **Southern of *Eco*RI-digested DNAs from 2/3MSYq^-^, 9/10MSYq^-^, and MSYq^- ^males together with control XY^RIII^, showing reduced hybridization in 2/3MSYq^- ^males and an apparent absence of hybridization to Y specific bands in the males with more extensive MSYq deficiency. **(e) **Two week exposure of a Southern blot of *Eco*RI digested DNAs from 9/10MSYq^- ^and MSYq^- ^males together with XO and XX females, and an underloaded XY^RIII ^control. X derived fragments now cross-hybridize with the *Sly *probe (dosing with the number of X chromosomes), but in addition there are at least three male specific bands (outlined) retained in the 9/10MSYq^- ^males that are absent in the MSYq^- ^males.

We next used the microarrayed *Sly *clone MTnH-K10 and the *Asty *exon 4 probe to hybridize to Southern blots of DNA samples from the MSYq deficient and control mice. The *Sly *probe revealed multiple hybridizing bands in control males that were reduced in intensity in 2/3MSYq^- ^males and exhibited no detectable hybridization in 9/10MSYq^- ^or MSYq^- ^males, or in females (Figure 5a, c). However, after long exposure of a further blot that included DNA from XX and XO females, multiple bands were detected in 9/10MSYq^- ^and MSYq^- ^males that were also present in XX and XO females (Figure 5e). In the females the band intensities dosed with the number of X chromosomes and thus presumably were due to cross-hybridization with *Xlr*/*Xmr*. However, at least three bands that are absent from females were retained in 9/10MSYq^- ^males, but were absent from MSYq^- ^males. Furthermore, a genomic PCR for the first *Sly *intron amplified from 9/10MSYq^- ^but not from MSYq^- ^DNA. This is consistent with the finding of *Sly *transcripts by RT-PCR in 9/10MSYq^- ^males but not in MSYq^- ^males. The *Asty *exon 4 probe also detected multiple hybridizing bands in the control males that reduced in intensity in the 2/3MSYq^- ^males (Figure 5b). There was a faintly hybridizing band in females, presumably due to cross-hybridization with *Astx*. The blot with all three MSYq deficient DNAs exhibited no hybridizing bands in 9/10MSYq^- ^and MSYq^- ^that were of the sizes of the four predominant Y-specific bands seen in the controls, but there was some unexplained intense hybridization at the level of the presumed *Astx *band and above.

### *Sly*, *Asty *and *Asty*(rec) are expressed in the testis during spermiogenesis

**Figure 6:**
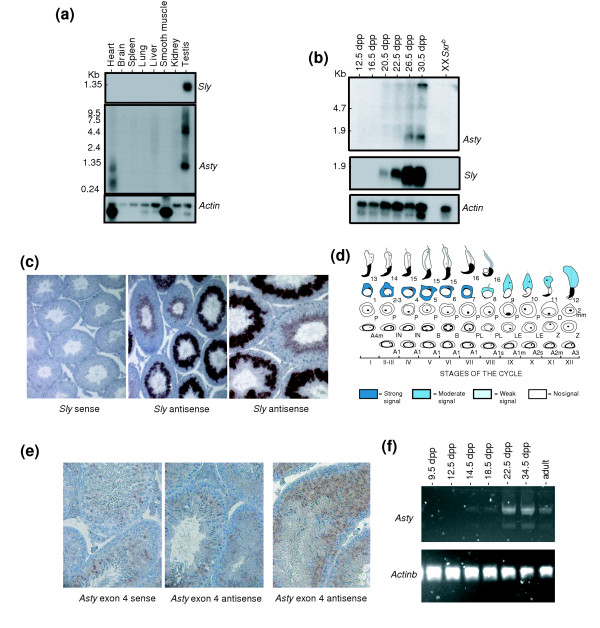
*Sly *and *Asty*/*Asty*(rec) are expressed in spermatids. **(a) **A multi-tissue northern blot of polyA^+ ^RNA probed with *Sly *clone MTnH-K10 and *Asty *exon 4 probe. *Sly *transcripts are restricted to the testis, but *Asty*/*Asty*(rec) transcripts are also seen in the heart. **(b) **A northern blot of total testis RNA from testes of mice aged 12.5-30.5 days postpartum (dpp) hybridized with the same *Sly *and *Asty *probes. No transcripts are detectable before 20.5 dpp, suggesting that the transcripts are restricted to spermatid stages. **(c) **RNA *in situ *analysis confirming that *Sly *transcription in the testis is predominantly if not exclusively in spermatids. **(d) **Diagram summarizing the expression of *Sly *in spermatids throughout the spermatogenic cycle. The spermatogenic stages starting from the basal layer and reading left to right are: spermatogonia: A1 through to B; meiotic prophase spermatocytes: preleptotene (PL), leptotene (LE), zygotene (Z), pachytene (P), diplotene (D); meiotic divisions (mm); postmeiotic spermatid stages: 1-13. **(e) **RNA *in situ *analysis with the *Asty *exon 4 probe that should detect *Asty *and *Asty*(rec) transcripts. The most convincing signal is in round spermatid nuclei, but this is seen with the antisense and sense (control) probes. However, the previously reported cDNA clone BU936708 (Additional data file 3) contains *Asty *exon 4 sequence in the antisense oritentation and which would hybridize with the sense probe. **(f) **Exon 1-4 *Asty *reverse transcriptase polymerase chain reaction from 9.5 dpp to adult testes. Sequencing of the cloned amplification products confirmed the presence of full-length *Asty *transcripts at 14.5 dpp, and a shorter transcript lacking exon 3 from 22.5 dpp.

Probing a multiple mouse tissue polyA northern blot (Figure 6a) with *Sly *clone MtnH-K10 indicated that *Sly *transcription is restricted to the testis, but the *Asty *exon 4 probe also detected transcripts in heart, consistent with there being a Y encoded EST from aorta (CA584558). In northern blots of RNAs from 12.5 days postpartum (dpp) to 30.5 dpp testes (Figure 6b), *Sly *and *Asty/Asty*(rec) transcripts were first detectable at 20.5 dpp, suggesting they are restricted to spermatid stages. To confirm spermatid expression we used the same probes for RNA *in situ *on testis sections, and for *Sly *(Figure 6c, d) high level spermatid specific expression was confirmed. For *Asty*/*Asty*(rec) hybridization was at a level not markedly above background, but it did appear to locate to round spermatids, particularly with respect to hybridization within the nucleus; however, there was a similar localization to round spermatids in the sense control (Figure 6e). We then found that the previously described transcript BU936708 derives from Y 'loci' that transcribe through *Asty *loci in the antisense orientation and include antisense sequence from *Asty *exon 4 (Additional data file 3); this transcript will hybridize to the sense control probe. Thus, we suspect that the round spermatid nuclear localization with the antisense probe does reflect the presence of *Asty*/*Asty*(rec) transcripts. However, it is apparent from the northern analysis and *in situ *analysis that *Asty*/*Asty*(rec) transcripts are much less abundant than those of *Sly*, which is consistent with there being five *Sly *clones but no *Asty*/*Asty*(rec) clones on the testis cDNA microarray.

Because we believe the transcripts detected by the *Asty *exon 4 probe derive predominantly from the *Asty*(rec) loci that are almost certainly driven by the spermatid specific *Ssty1 *promotor, we made attempts to determine whether true *Asty *transcripts are also spermatid specific. For this we used an *Asty *exon 1-4 primer pair (previously used to provide evidence for *Asty *transcripts) to amplify testis cDNA samples from 1.5 dpp to adult. Transcripts were weakly detected by these primers at 14.5 and 18.5 dpp, but the predominant expression was at 22.5 dpp onward when there were transcripts of two sizes (Figure 6f).

We know these primers can also amplify *Astx *transcripts, and so we sequenced cloned RT-PCR products to confirm their identity. This confirmed the presence of the two previously identified *Asty *transcripts (one of which lacks exon 3). The longer transcript was detected from 14.5 dpp onward, and the shorter transcript from 22.5 dpp onward. Because spermatids first appear at about 20 dpp, we conclude that the shorter *Asty *isoform appears to be spermatid specific, but that the longer one is not spermatid specific.

### The protein encoding potential of *Sly *and *Asty *family members and their X relatives

**Figure 7:**
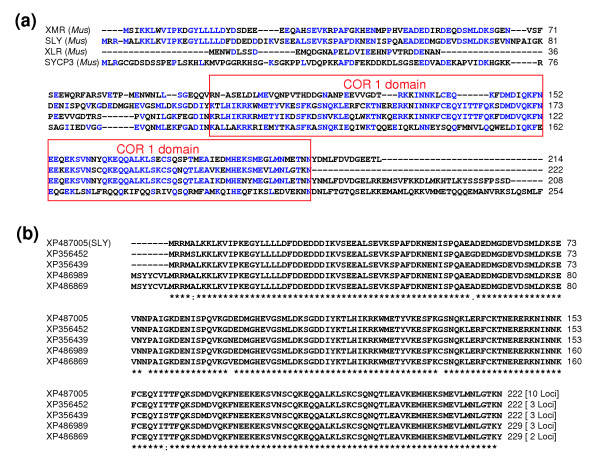
The protein encoding potential of *Sly*. **(a) ***Sly *encodes a putative protein with a COR1 region. This COR 1 region is shared with two closely related proteins, namely XMR and XLR, and with the less closely related synaptonemal complex protein SYCP3. **(b) **A comparison of the predicted protein sequence for SLY with the predicted proteins for other *Sly *related Y loci that are each represented by more than one copy (information collated from sequence information available in December 2004).

Of the 65 loci of the *Sly *gene family, 34 have retained coding potential for a protein that is related to three previously described chromatin associated nuclear proteins: XLR and XMR, which are encoded by members of a complex multicopy X gene family [28,30-32], and the autosomally encoded SYCP3 protein, via a conserved COR1 region [33,34] (Figure. 7a, b). The putative SLY protein is most similar to XMR (amino acid identities: XMR 48%, XLR 46% and SCP3 30%), but within the COR1 region it is most similar to XLR (amino acid identities: XLR 79%, XMR 50% and SCP3 37%).

**Figure 8:**
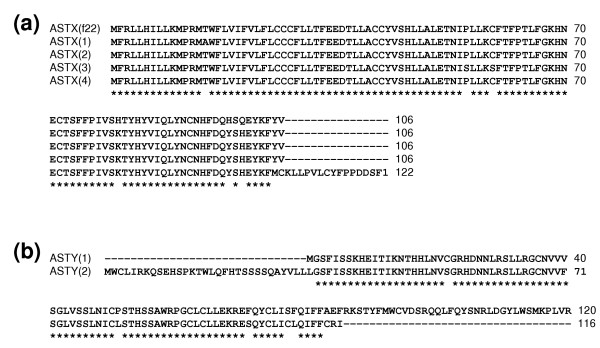
A comparison of the protein encoding potentials of *Astx *and *Asty*. **(a) **The predicted protein for the conserved open reading frame in exon 4 of the arrayed *Astx *clone (f22) and for the four protein variants predicted from the eight putative X chromosomal loci. **(b) **The two predicted ASTY protein variants encoded by two overlapping open reading frames in exon 1 present in six *Asty *loci.

All eight *Astx *loci have a modest open reading frame (ORF) in exon 4, six of which would encode a protein of 106 amino acids and two would encode a protein with a carboxyl-terminal extension to 122 amino acids (Figure 8a). Surprisingly, given the greater than 92% sequence identity of the *Asty *and *Astx *loci, this exon 4 ORF is not conserved in any of the putative *Asty *loci so far identified. There are six *Asty *loci with an ORF in exon 1 that could encode proteins of 116 or 120 amino acids (Figure 8b). However, the *Asty *sequence we obtained by RT-PCR from testis cDNA (Additional data file 2) did not match the sequence of this subset of loci with an exon 1 ORF; thus, it appears likely that the Y-linked members of this family no longer produce a functional protein. Assessing whether any of the novel *Asty*(rec) transcripts have significant protein encoding potential will require detailed characterization of this complex family of transcripts.

## Discussion

The objective of the present study was to try to establish whether members of the *Ssty *gene family are the only Y genes present on MSYq that are expressed in the testis during sperm development. Our strategy was to use testis cDNA microarrays to identify transcripts that are reduced or absent in the testes of mice with MSYq deficiencies, and then to determine their chromosomal assignments. This strategy led to the identification of further testis transcripts unrelated to *Ssty*, which proved also to be encoded by multicopy Y loci on MSYq and expressed in spermatids.

The first of these, *Sly *(*Sycp3*-like Y-linked; MGI:2687328) is most closely related to the multicopy *Xlr*/*Xmr *gene family, which is located on the X chromosome. It had previously been reported, based on Southern blot evidence, that multiple *Xlr *related sequences are present on the X and Y chromosomes, but it was concluded that most if not all of these *Xlr *related sequences were likely to represent nontranscribed pseudogenes [35]. However, another X linked family member, namely *Xmr*, was subsequently described that is specifically expressed in the testis [28], and our present findings establish that *Sly *transcripts, encoded by multiple loci on MSYq, are also abundantly expressed in the testis in spermatids.

The XLR protein is a thymocyte nuclear protein that has been shown to colocalize with SATB1, a protein that binds to AT-rich sequences at the base of chromatin loops [31,36]. XMR is a testis specific nuclear protein that concentrates in the sex body of pachytene spermatocytes as the chromatin begins to condense [28]. SYCP3 is an autosomally encoded protein that is part of the synaptonemal complex of chromosomes in meiosis. XLR, XMR and SYCP3, together with the putative FAM9 proteins encoded by the human X chromosome, have been grouped into a superfamily (InterPro accession number IPR006888, PFAM accession number PF04803) because they all share a conserved Cor1 domain. The Cor1 domain of the putative SLY protein is very similar (79% identity) to that of XLR, and we already have preliminary evidence that an SLY protein is produced; we predict that this SLY protein will also associate with chromatin loops.

**Figure 9:**
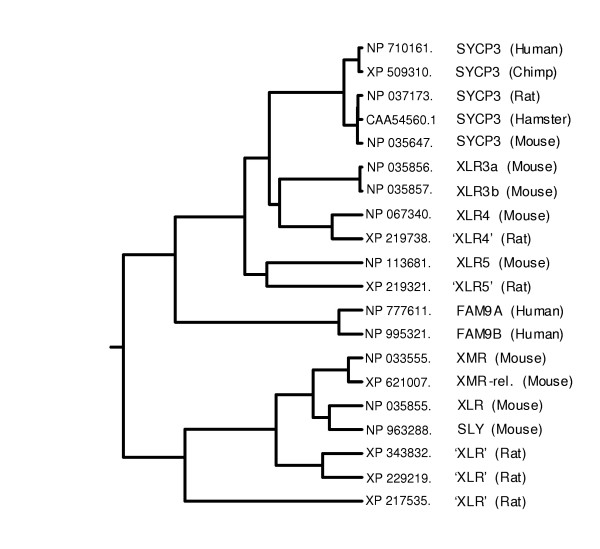
Dendrogram showing the relationship between the SLY protein and other identified or predicted mammalian Cor1 domain proteins. The autosomally encoded SYCP3 is the presumed ancestral gene because it has been identified in a wide range of vertebrate species. The remaining proteins other than SLY are X encoded. For the rat, the putative X-encoded proteins have not yet been named and the labels given are simply a reflection of their position within the dendrogram. The massive expansion in copy number is only seen in the branch containing XMR, XMR-rel., XLR and SLY. This amplification therefore occurred subsequent to the divergence of mouse and rat lineages, concurrent with the appearance of *Sly *on the Y chromosome.

Because SYCP3 is a fundamental component of the synaptonemal complex during meiosis, it is not surprising that it has been identified in a wide range of vertebrates. A comparison of mammalian Cor1 domain proteins (Figure 9) indicates that *Sycp3 *is the ancestral gene and that some time before the divergence of human and mouse lineages the gene came onto the X chromosome and became multicopy; these copies then evolved rapidly and independently. At some point after the divergence of mouse and rat, one of the X copies was brought on to the Y chromosome, creating a new X-Y homologous subfamily. This subfamily subsequently became massively amplified in copy number on both the X and Y chromosomes. Like *Sly*, *Xmr *and its close relatives are highly expressed in spermatids [37], and our recent finding that a major transcriptional consequence of MSYq deficiency is the upregulation of a number of spermatid-expressed X genes, including *Xmr*, leads us to suspect that amplification of *Xmr *and *Sly *is a result of 'genomic conflict' between sex linked meiotic drivers and suppressors [37,38].

The second MSYq encoded spermatid transcript, *Asty*, is also encoded by a multicopy Y gene. *Asty *has a very high degree of homology (92-94% identity across exons and introns) to a multicopy X gene (*Astx*), suggesting that it may be a recent arrival on the mouse Y chromosome. Intriguingly, BLAST searches with the microarrayed *Astx *clone sequence failed to detect similar sequences in the human genome, but in the rat there were four X-linked sequences matching the last third of the mouse *Astx*/*Asty *loci. Future analysis of these putative rat loci, in particular to determine whether related sequences are present on the rat Y chromosome, may help to delineate the evolutionary history of this gene family. In addition to these *Asty *loci, we have identified novel 'recombinant' loci, apparently driven by the *Ssty1 *promotor, that incorporate a subset of *Ssty1 *and *Asty *exons, and through alternative splicing and serendipitous splicing of more downstream sequences they have the potential to produce a range of novel transcripts in addition to the previously described transcripts AK016790 and AK015935.

Because all eight copies of *Astx *have retained a similar ORF in exon 4, it is reasonable to predict that they encode a protein. Interestingly, none of the more than 100 putative *Asty *loci have retained the *Astx *exon 4 ORF, despite the greater than 92% sequence identity of these loci. Six copies of *Asty *do have protein encoding potential in exon 1; however, the only *Asty *transcripts we have thus far identified do not derive from this subset of loci with exon 1 protein encoding potential. Overall, this evidence suggests that *Astx *is probably translated and that *Asty *is not. This does not, however, rule out a functional role for *Asty*, or indeed the more strongly transcribed *Asty*(rec), in sperm differentiation, especially given the increasing literature on functional RNAs. A particularly pertinent example in the present context is provided by the Stellate/suppressor of Stellate system in *Drosophila*, in which transcripts encoded by multicopy loci on the Y regulate expression of a protein expressed in spermatids and encoded by related multicopy loci on the X chromosome [39], via an antisense/small interfering RNA mechanism. It has been postulated that these RNA mediated regulatory interactions between multiple X and Y loci in *Drosophila *arose as a consequence of a past postmeiotic genomic conflict [38,40] and there are clear parallels with the regulatory interactions we have uncovered between MSYq-encoded loci and spermatid-expressed X genes [37]. In this regard it is important that we have established that *Astx *transcripts are present in spermatids (Additional data file 4) as well as being expressed in spermatocytes (BF019211, CF198098).

The identification of additional MSYq encoded, spermatid expressed transcripts provides alternatives to *Ssty *deficiency as to the cause of the abnormalities in sperm shape associated with MSYq deficiencies. Three features suggest that *Sly *deficiency is the more likely cause. First, and importantly, retention of one or more transcribed *Sly *copies in 9/10MSYq^- ^males (in contrast to *Asty *and *Ssty *[8]) provides a potential explanation for the less severe sperm abnormalities in these males, as compared with MSYq^- ^males that completely lack *Sly*. Second, *Sly *is predicted to encode a chromatin associated protein, and the related proteins encoded by *Xlr *and *Xmr *are expressed at sites of chromatin restructuring. Thus, it is reasonable to suppose that *Sly *deficiency might affect sperm head shape by disturbing chromatin organization in the nucleus. Third, *Sly *is the most strongly transcribed in spermatids. On the other hand, we consider the sex ratio distortion seen in 2/3MSYq^- ^males to be a consequence of disturbing the balance between sex-linked meiotic drivers and suppressors involved in X-Y gene conflict [37], and because this balance may have been achieved by MSYq encoded proteins or RNAs, or both, all MSYq gene families remain plausible candidates.

## Conclusion

The highly repetitive nature of the mouse Y chromosome long arm presents formidable challenges for the determination of its functional gene content. Our strategy of using expression array data to highlight transcriptionally active loci among the sea of partial and degenerate gene copies has proved successful in identifying further MSYq encoded transcripts, deficiency of which may contribute to the abnormal sperm head development and function seen in males with MSYq deficiencies. This type of approach is likely to form an important component of future functional analysis of mammalian Y chromosomes and other repetitive chromosomal regions.

## Materials and methods

### Mice

All mice were produced on a random bred albino MF1 strain (NIMR colony) background. The mice used to provide RNA for microarray analysis were the three MSYq deficient genotypes (Figure 1) that we have previously analyzed with respect to *Ssty *expression and sperm abnormalities, together with appropriate age- and strain-matched controls.

#### XY^RIII^qdel males (2/3 MSYq^-^)

These mice have an RIII strain Y chromosome with a deletion removing approximately two thirds of MSYq. The sperm have mild distortions of head shape; the mice are nevertheless fertile with a distortion of the sex ratio in favor of females [3]. XY^RIII ^males are the appropriate controls.

#### XY^*Tdym*1^qdelSry males (9/10 MSYq^-^)

These mice have a 129 strain Y chromosome with a deletion removing approximately nine-tenths of MSYq, and also a small deletion (*Tdy*^m1^) removing the testis determinant *Sry *from the short arm, this deficiency being complemented by an autosomally located *Sry *transgene. These males are sterile with virtually all of the sperm having grossly distorted heads [8]. XY^*Tdym*1^*Sry *males are the appropriate controls.

#### XSxr^*a*^Y*X males (MSYq^-^)

In these mice the only Y specific material is provided by the Y short arm derived *Sxr*^*a *^factor, which is attached to the X chromosome distal to the pseudo-autosomal region (PAR); the Y* ^X ^chromosome provides a second PAR, thus allowing fulfillment of the requirement for PAR synapsis [41]. These males lack the entire Y specific (non-PAR) gene content of Yq; they also have a 7.5-fold reduction in copies of the *Rbmy *gene family, located on the short arm adjacent to the centromere. The males are sterile and have even more severe sperm head defects than do XY^-^qdel*Sry *males [8,41]. Our recent work indicates that *Rbmy *deficiency is unlikely to be a significant cause of the abnormal sperm development [42]. Because *Sxr*^*a *^originated from a Y^RIII ^chromosome, the appropriate controls are again XY^RIII^.

### Sample collection and microarray analysis

Testes were obtained from two of each of the MSYq deficient males and the two control genotypes, at 2 months of age. Total RNA was isolated using TRI reagent (Sigma-Aldrich, Poole, Dorset, UK) and cleaned using RNEasy columns (Qiagen, Crawley, West Sussex, UK), in accordance with to the manufacturers' protocols. Microarray hybridizations and analysis were carried out as described by Ellis and coworkers [43], except that microarray data normalization was based on the global median signal for Cy3 and Cy5 channels rather than on a panel of control genes. This form of normalization is valid because the majority of genes do not vary between the mutant and control samples for any of the models (Figure 2a). Briefly, 10 μg total RNA was fluorescently labeled using an indirect protocol, and the test and control samples were allowed to co-hybridize to the array. Four technical replicate slides were obtained for each test/control comparison. Cy3 was used to label the test (mutant) sample and Cy5 the control (normal) sample in all cases. Fluorescence intensities provide measures of the relative abundance for each hybridizing testis transcript for each genotype, whereas Cy3/Cy5 ratios for individual clones provide a measure of the levels in each of the MSYq deficient models relative to their matched controls.

The arrayed clones consisted of two subtracted adult mouse testis libraries [43], six testis cell type separated libraries (IMAGE clone plate numbers 8825-8830, 8831-8836, 8846-8850, 9339-9342, 13869-13871, 13872-13874 [29]), and appropriate controls [43]. From sequence analysis performed to date, the combined gene set included cDNAs derived from more than 4,000 genes, often represented by multiple clones, enriched for cDNAs deriving from spermatogenic cells. Slides were scanned using an ArraywoRx CCD-based scanner and the resulting images quantitated using GenePix. Raw data were processed in Excel to remove data from spots flagged as bad or not found, and from features with a background subtracted intensity of under 100 in both channels. Global median normalization, *t* tests, and fold change filtering were performed using GeneSpring. Full details of the array experiment were submitted to the ArrayExpress database (accession number E-MEXP-251).

### Southern blot analysis

The probes used for Southern analysis were *Sly *cDNA clone BC049626 [26] and an *Asty *314 bp exon 4 probe amplified with primers CAGCAAGGAGAGTGGGGAGTA and CAGTGGGATGTTGGTTTCTAATG. Genomic DNA was extracted from tail biopsies and 15 μg (or 4 μg for the control XY on the long exposure Southern blot) was digested with *Eco*RI, electrophoresed through a 0.8% agarose gel and transferred to a Hybond-N membrane (Amersham Biosciences, Little Chalfont, Buckinghamshire, UK). After UV crosslinking (StrataLinker™. Stratagene, La Jolla, CA, USA), the membrane was hybridized overnight with ^32^P-labelled probes either at low stringency (55°C; hybridization buffer: 6 × salt sodium citrate (SSC), 5 × Denhart's, 0.1% sodium dodecyl sulfate (SDS), 100 μg/ml salmon sperm DNA; two 30 minute washes (one with 0.5 × SSC and 0.1% SDS, and one with 0.1 × SSC and 0.1% SDS)) or at high stringency (60°C; hybridization buffer: 6 × SSC, 5 × Denhart's, 0.5% SDS, 100 μg/ml salmon sperm DNA; two 30 minute washes (one with 0.5 × SSC and 0.1% SDS, and one with 0.1 × SSC and 0.1%SDS)). The membrane was exposed to X-ray film or Phosphorimager screen overnight.

### Northern analysis

The probes used for northern analysis were as follows: *Sly *cDNA clones BC049626 [26] and MtnH_K10 from the microarray (Additional data file 1), and the *Asty *314 bp exon 4 probe used for northern analysis. An actin probe that recognizes α- and β-actin transcripts [44] served as a control for RNA integrity. Total RNA (20 μg) was electrophoresed in a 1.4% formaldehyde/agarose gel and transferred to Hybond-N membrane (Amersham) using 20 × SSC buffer. The RNA was cross-linked to the membrane with UV (StrataLinker™), the membrane was fixed for 1 hour at 80°C, and hybridized overnight at 60°C with ^32^P-labelled probes in hybridization buffer (6 × SSC, 5 × Denharts, 0.1% SDS, 50 mmol/l sodium phosphate, 100 μg/ml salmon sperm DNA). After two 60°C washes (30 min with 0.5 × SSC and 0.1% SDS, and 30 min with 0.1 × SSC and 0.1% SDS) the membrane was exposed to X-ray film for 5 hours and subsequently to a Phosphorimager screen to allow quantitation of hybridization using ImageQuant software.

### RT-PCR analysis

RNA samples were treated for DNA contamination using DNAse I amplification grade kit (Invitrogen, Paisley, UK). For the *Sly*/*Xmr *RT-PCR, 2 μg total RNA was reverse transcribed in a 40 μl reaction using standard procedures. A 2.5 μl aliquot was then added to a 25 μl PCR reaction. RT-PCR for *Astx*/*Asty *was performed using the Qiagen OneStep RT-PCR kit, following the manufacturers' instructions. In both cases amplification was for 30 cycles at an annealing temperature of 60°C.

The primers used were as follows: *Sly *and *Xmr*, forward primer GTGCGGTTTGGAAGTGT and reverse primer CTCAAGCAGAAGCAGATG; *Asty *and *Astx *exon 4, forward *Asty *primer GRGGAGTAGAACTCATCATC and forward *Astx *primer GGGGAGTAGAACTCATCTTTA, with common reverse primer CAGGAGATGACTAACATAGCA; *Asty *exon 1 to exon 4, forward GGCCTTGCTCTTATGTCATC and reverse CGATGATGAGTGACCTAAAGAT; and *Astx *exon 1/2 (spanning intron 1) to 3/4 (spanning intron 3), forward GCTCCAGAAGACAGAGATAC and reverse AGACTTCAAACCTCATGCAGT.

### Sequencing

RT-PCR product was purified using the QiaQuick kit (Qiagen) and cloned using the pGEM-T easy vector system I kit (Invitrogen). Sequencing reactions were performed from 5' and 3' ends using standard protocols (BigDye; Amersham). Completed reactions were analyzed by the Cambridge Department of Genetics sequencing service using a 3130xl capillary system (Applied Biosystems, Warrrington, Chesire, UK). Cycling conditions for the sequencing reactions were 96°C, 55°C and 60°C for 10 s, 5 s and 4 minutes, respectively.

### RNA *in situ *analysis

*In situ *hybridization using digoxigenin-labeled cRNA probes from *Sly *clone MtnH_K10 and *Asty *and *Astx *exon 4 were used to localize each mRNA on Bouin-fixed paraffin-embedded mouse testis sections using procedures previously described [45], with hybridization and washing temperatures up to 55°C. Both antisense and sense (negative control) cRNAs were used on each sample, in every experiment, and for each set of conditions tested.

### RNA fluorescence *in situ *hybridization

RNA fluorescence *in situ *hybridization (FISH) was performed basically as described previously for Cot1 RNA FISH [46] using an X chromosomal BAC clone RP23-83P7 that contains the *Astx *locus encoding the cDNA AK076884 (BACPAC Resources, Oakland, CA, USA). Hybridization reactions consisted of 100 ng biotin-labeled BAC probe, 3 μg mouse Cot1 DNA and 10 μg salmon sperm DNA, and were carried out overnight at 37°C. Staging of spermatogenic cells was based on DAPI fluorescence morphology, together with immunolabelling for the synaptonemal complex protein SYCP3 (rabbit anti-SYCP3; Abcam, Cambridge, UK) and the phosphorylated form of histone H2AX (mouse anti-gamma H2AX; Upstate, Dundee, UK), as previously described [46,47]. The chromosomal source of the RNA FISH signals was first checked by DNA FISH with a digoxigenin labeled RP23-83P7 BAC probe prepared using the Digoxigenin Nick Translation Kit (Roche Diagnostics, Lewes, East Sussex, UK). Hybridizations were carried out as for RNA FISH with stringency washes as described previously [46], and the DNA FISH signals were developed using anti-DIG-FITC (Chemicon, Chandlers Ford, Hampshire, UK), diluted 1:10, for 1 hour at 37°C. Further confirmation of the X chromosomal source of the *Astx *transcripts was provided by X chromosome painting, as previously described [46].

## Additional data files

The following additional data are available with the online version of this paper: a file providing sequence information for the *Sly*-related clones from the microarray (Additional data file 1); a file providing sequence information for the microarrayed *Astx *clone and for the *Asty *RT-PCR products (Additional data file 2); a diagram providing sequence information on the 'recombinant' loci encoding the transcripts AK016790 and AK015935 (Additional data file 3); and images showing *Astx *transcriptional analysis (Additional data file 4).

## Supplementary Material

Additional data file 1 A file providing sequence information for the *Sly*-related clones from the microarray Click here for file

Additional data file 2 A file providing sequence information for the microarrayed *Astx *clone and for the *Asty *RT-PCR products Click here for file

Additional data file 3 A diagram providing sequence information on the 'recombinant' loci encoding the transcripts AK016790 and AK015935 Click here for file

Additional data file 4 Images showing *Astx *transcriptional analysis Click here for file
