# Correction to: Identification of novel Y chromosome encoded transcripts by testis transcriptome analysis of mice with deletions of the Y chromosome long arm

**DOI:** 10.1186/s13059-019-1779-z

**Published:** 2019-08-09

**Authors:** Aminata Touré, Emily J. Clemente, Peter J. I. Ellis, Shantha K. Mahadevaiah, Obah A. Ojarikre, Penny A. F. Ball, Louise Reynard, Kate L. Loveland, Paul S. Burgoyne, Nabeel A. Affara

**Affiliations:** 1 Division of Developmental Genetics, MRC National Institute for Medical Research, Mill Hill, London, NW7 1AA UK; 2 Department of Pathology, University of Cambridge, Tennis Court Road, Cambridge, CB2 1QP UK; 3 Monash Institute of Medical Research, Monash University, and The Australian Research Council Centre of Excellence in Biotechnology and Development, Melbourne, Victoria 3168 Australia

**Correction to: Genome Biol (2005) 6:R102**

**https://doi.org/10.1186/gb-2005-6-12-r102**

Following publication of the original article [1], the following error was reported:

The actin control panel in Fig. 3 of this paper is reproduced from Fig. 7 of Touré et al., 2004 [2] by kind permission of the Genetics Society of America. Touré et al., 2004 used Northern blotting to show that the Y-linked genes Ssty1 and Ssty2 have reduced expression in a range of mouse genotypes with deletions on the Y chromosome long arm. This paper shows that two novel genes, Sly and Asty are also present on mouse Yq and have reduced expression in these deleted genotypes. A further companion paper was published in Human Molecular Genetics (Ellis et al., 2005 [3]) showing that X-linked genes are upregulated in the various deleted genotypes. Since two of the genotypes concerned are sterile and very hard to generate, all the Northern blot experiments in these papers were performed on a single membrane that was stripped and re-probed with a range of different X- and Y-linked genes. The same beta-actin loading control image thus necessarily applies to all the data presented, and was shown in all three papers. We regret that this was not mentioned appropriately in the Methods and figure legends at the time of publication.

This correction article also provides alternate correspondence email addresses: aminata.toure@inserm.fr; P.J.I.Ellis@kent.ac.uk
