# Supplementary material for: Identification of novel Y chromosome encoded transcripts by testis transcriptome analysis of mice with deletions of the Y chromosome long arm
Source: Genome Biol. 2005 Dec 2;6(12):R102. doi: 10.1186/gb-2005-6-12-r102 (PMC1414076; doi:10.1186/gb-2005-6-12-r102)
Supplement: Additional data file 1 — A file providing sequence information for the Sly-related clones from the microarray [file gb-2005-6-12-r102-S1.pdf]

Additional Data File 1.

The 5 *Sly* clones from the microarray in Clustal alignment with the cDNA clone BC049626. The original cDNAs were digested with Rsa1 (cut site GT'AC). MTnF_J16 extends further 3’ because there is a single base change converting a GT'AC site to GTTC. MTnE_N09 lacks *Sly* exons 5 and 6.

```
BC049626  ---------ACAGAAGGGTGCGGTTTGGAAGGTGTTCTCCTCTTAGATGAGCTACTACTGAGTTCTTATGAGAAGAATGGCTCTTAA 78
MtnH_K10  ---------------------------------------------------------------------------------------
MtnE_N09  GAGCTAAGCACAGAAGGGTGCGGTTTGGAAGGTGTTCTCCTCTTAGATGAGCTACTACTGAGTTCTTATGAGAAGAATGGCTCTTAA 87
MtnF_J16  ---------------------------------------------------------------------------------------
MtnB_M09  ---------------------------------------------------------------------------------------
Mtn14_G18 ---------------------------------------------------------------------------------------
                   ******************************************************************************


GAAATTGAAGGTGATACCAAAGGAAGGTTACTTATTACTTTTGGACTTTGATGATGAGGACGATGACATAAAAGTTTCAGAGGAGGCTCTTTCGGAA 175
----------------------------------------------------GATGAGGACGATGACATAAAAGTTTCAGAGGAGGCTCTTTCGGAA 45
GAAATTGAAGGTGATACCAAAGGAAGGTTACTTATTACTTTTGGACTTTGATGATGAGGACGATGACATAAAAGTTTCAGAGGAGGCTCTTTCGGAA 184
-------------------------------------------------------------------------------------------------
-------------------------------------------------------------------------------------------------
-------------------------------------------------------------------------------------------------
*************************************************************************************************


GTAAAGAGCCCAGCATTTGATAAAAATGAGAATATATCGCCTCAAGCAGAAGCAGATGAAGATATGGGAGATGAAGTAGACAGTATGTTGGATAAAT 272
GTAAAGAGCCCAGCATTTGATAAAAATGAGAATATATCGCCTCAAGCAGAAGCAGATGAAGATATGGGAGATGAAGTAGACAGTATGTTGGATAAAT 142
GTAAAGAGCCCAGCATTTGATAAAAATGAGAATATATCGCCTCAAGCAGAAGCAGATGAAGATATGGGAGATGAAGTAGACAGTATGTTGGATAAAT 281
-------------------------------------------------------------------------------------------------
-------------------------------------------------------------------------------------------------
-------------------------------------------------------------------------------------------------
*************************************************************************************************
```

```
CTGAAGTAAATAATCCAGCAATTGGTAAAGATGAAAATATATCGCCTCAAGTAAAAGGAGATGAAGACATGGGACATGAAGTAGGCAGTATGTTGGA 369
CTGAAGTAAATAATCCAGCAATTGGTAAAGATGAAAATATATCGCCTCAAGTAAAAGGAGATGAAGACATGGGACATGAAGTAGGCAGTATGTTGGA 239
CTGAAG------------------------------------------------------------------------------------------- 287
-------------------------------------------------------------------------------------------------
-------------------------------------------------------------------------------------------------
-------------------------------------------------------------------------------------------------
*************************************************************************************************

TAAATCTGGAGATGACATTTATAAGACGCTTCACATAAAGAGAAAATGGATGGAAACTTATGTCAAAGAATCTTTCAAAGGCAGCAACCAGAAATTA 466
TAAATCTGGAGATGACATTTATAAGACGCTTCACATAAAGAGAAAATGGATGGAAACTTATGTCAAAGAATCTTTCAAAGGCAGCAACCAGAAATTA 336
-----------ATGACATTTATAAGACGCTTCACATAAAGAGAAAATGGATGGAAACTTATGTCAAAGAATCTTTCAAAGGCAGCAACCAGAAATTA 373
-------------------------------------------------------------------------------------------------
-------------------------------------------------------------------------------------------------
-------------------------------------------------------------------------------------------------
*************************************************************************************************

GAAAGATTTTGCAAAACGAACGAACGAGAGAGGAAGAACATCAACAACAAATTTTGTGAGCAGTATATAACTACATTTCAGAAGTCTGATATGGATG 563
GAAAGATTTTGCAAAACGAACGAACGAGAGAGGAAGAACATCAACAACAAATTTTGTGAGCAGTATATATCTACATTTCAGAAGTCTGATATGGATG 433
GAAAGATTTTGCAAAACGAACGAACGAGAGAGGAAGAACATCAACAACAAATTTTGTGAGCAGTATATAACTACATTTCAGAAGTCTGATATGGATG 470
-------------------------------------------------------------------------------------------------
-------------------------------------------------------------------------------------------------
-------------------------------------------------------------------------------------------------
*************************************************************************************************

TACAGAAATTCAATGAAGAAAAAGAAAAATCAGTGAATAGTTGTCAAAAAGAACAACAAGCATTGAAACTGTCCAAATGTAGTCAGAACCAGACCCT 660
T------------------------------------------------------------------------------------------------ 434
TACAGAAATTCAATGAAGGAAAAGAAAAATCAGTGAATAGTTGTCAAAAAGAACAACAAGCATTGAAACTGTCCAAATGTAGTCAGAACCAGACCCT 567
-ACAGAAATTCAATGAAGAAAAAGAAAAATCAGTGAATAGTTGTCAAAAAGAACAACAAGCATTGAAACTGTCCAAATGTAGTCAGAACCAGACCCT 96
-ACAGAAATTCAATGAAGAAAAAGAAAAATCAGTGAATAGTTGTCAAAAAGAACAACAAGCATTGAAACTGTCCAAATGTAGTCAGAACCAGACCCT 96
-ACAGAAATTCAATGAAGAAAAAGAAAAATCAGTGAATAGTTGTCAAAAAGAACAACAAGCATTGAAACTGTCCAAATGTAGTCAGAACCAGACCCT 96
****************** ******************************************************************************

GGAAGCAGTTAAAGAAATGCATGAGAAGTCCATGGAGGTTTTGATGAACTTGGGGACCAAGAACTAAGAAATGCTTTTTGGTGTAGATGGTGAACTG 757
-------------------------------------------------------------------------------------------------
GGAAGCAGTTAAAGAAATGCATGAGAAGTCCATGGAGGTTTTGATGAACTTGGGGACCAAGAACTAAGAAATGCTTTTTGGTGTAGATGGTGAACTG 664
GGAAGCAGTTAAAGAAATGCATGAGAAGTCCATGGAGGTTTTGATGAACTTGGGGACCAAGAACTAAGATATGCTTTTTGGTGTAGATGGTGAACTG 193
GGAAGCAGTTAAAGAAATGCATGAGAAGTCCATGGAGGTTTTGATGAACTTGGGGACCAAGAACTAAGATATGCTTTTTGGTGTAGATGGTGAACTG 193
GGAAGCAGTTAAAGAAATGCATGAGAAGTCCATGGAGGTTTTGATGAACTTGGGGACCAAGAACTAAGATATGCTTTTTGGTGTAGATGGTGAACTG 193
********************************************************************* ***************************
```

```
A-AAAAAAAAATGTCTATGTTTGAAAGAGCCATCATGGAGGATAATCTGAAGTACTCTTCTACTTTCCCATCTTCAGAAAAATGAAGCATGAAAAAT 853
-------------------------------------------------------------------------------------------------
AGAAAAAAAA-TGTCTATGTTTGAAAGAGCCATCATGGAGGATAATCTGAAGT-------------------------------------------- 716
AGAAAAAAAAATGTCTATGTTTGAAAGAGCCATCATGGAGAATAATCTGAAGTTCTCTTCTACTTTCCCATCTTCAGAAAAATGAAGCATGAAAAAT 290
AGAAAAAAAAATGTCTATGTTTGAAAGAGCCATCATGGAGAATAATCTGAAGT-------------------------------------------- 246
AGAAAAAAAAATGTCTATGTTTGAAAGAGCCATCATGGAGAATAATCTGAAGT-------------------------------------------- 246
* ******** ***************************** ************ *******************************************

TTTCACTTGCTGGTATATATATAAAACAAATAAAAAAATCTCTAACTTTTTTGTTTCCTACGAAAAAAAAAAAAAAAAAAAAAAAAAAAAAAA 946
---------------------------------------------------------------------------------------------
---------------------------------------------------------------------------------------------
TTTCACTTGCTGGTATATATATAAAACAAATAAAAAAATCTCTAACTTTTTTGTTTCCTATGAAACAGAAAAAAAAAAAAAAAAAAAAAA--- 380
---------------------------------------------------------------------------------------------
---------------------------------------------------------------------------------------------
************************************************************ **** * **********************
```
