# Supplementary material for: Identification of novel Y chromosome encoded transcripts by testis transcriptome analysis of mice with deletions of the Y chromosome long arm
Source: Genome Biol. 2005 Dec 2;6(12):R102. doi: 10.1186/gb-2005-6-12-r102 (PMC1414076; doi:10.1186/gb-2005-6-12-r102)
Supplement: Additional data file 2 — A file providing sequence information for the microarrayed Astx clone and for the Asty RT-PCR products [file gb-2005-6-12-r102-S2.pdf]

Additional Data File 2.

The *Astx* cDNA clone from the microarray in Clustal alignment with the *Asty* exon 1 – exon 4 sequences obtained from cloned RTPCR products using the primers highlighted in blue type. One of the Asty clones lacks exon 3. The position of the exon 4 open reading frame for *Astx* is highlighted in yellow.

```
Astx      ATTCTCTATCTGATGGAGGGCAAAAATTTCCATTTAACTGTGTCTCACATCGAACTCCTCATTTGAAAGGGGCACCAGAATGACTCTTTTTGTTCCAGCG 100

Astx      GTTCTCAAATTCACATCCTCTGTCGAAGAGGTCTGTAAATGTCATCCTGTCATACCAGGGCTTCAGTGCCTCTCCCTCTGCCTATGGGAGCCTCAGACAC 200

Astx      ACCACATCTAGTCATAAATATTTCCTCTTCCCCCTCTGTCTATTGAAGGCAACAAACCCTTCTCCTAATGCCCTATTGGACTGAAATATTTCCTTTTTTT 300

Astx      TCAGTGGTGGACTCATACCTACCCTCTCTTGCAAAGTGTAAATAGATCTTGTCTTATTGACTATGTGTGCCTGCTATATCCCAACTGTCTAAGATTACTT 400

Astx      CCAAACTTCTTCCTGCTACAAATATTCGGAGACATTCCAATATTCAAGAAAAGTGGCCTTGCTCTTTTGTCATCTGACTAAAGGCTTCATAGACCAAAGA 500
Asty1     ------------------------------------------------------GGCCTTGCTCTTATGTCATCTGACTAAAGGTTTCATAGACCAAAGA 46
Asty2     ------------------------------------------------------GGCCTTGCTCTTATGTCATCTGACTAAAGGTTTCATAGACCAAAGA 46
          **************************************   ***********************************************************

Astx      TAACCATCCAACCTGTTGGAATACTGAAACAATTGCTGCCTGCTGTCTACACACCACATAAAATATGTGGATATTCTAAATTTTGCAAAAAAGAATTGGA 600
Asty1     TAACCATCCAACTTGTTGGAATACTGAAACAATTGCTGCCTGCTGTCCACACACCACATAAAATATGTGGATTTCGTAAATTCTGCAAAAAAGGTTTGGA 146
Asty2     TAACCATCCAACTTGTTGGAATACTGAAACAATTGCTGCCTGCTGTCCACACACCACATAAAATATGTGGATTTCGTAAATTCTGCAAAAAAGGTTTGGA 146
          *********************************************** ********************************** *****************

Astx      AGCTGATCAAGCAATACTGGTACTTCCTCTGCTCCAGAAGACAGAGATACCCTGGCTTCCATGCAGAGGAATGTGTGGATGGACATATATTTATGGAAGA 700
Asty1     AGCTGATCAATCAATACTGGAACTCCCTCTTCTCCAGAAGA----GACATCCTGGCCTCCATGCAGAGGAATGTGTGGATGGACATATATTTAAGGAAGA 242
Asty2     AGCTGATCAATCAATACTGGAACTCCCTCTTCTCCAGAAGA----GACATCCTGGCCTCCATGCAGAGGAATGTGTGGATGGACATATATTTAAGGAAGA 242
          *****************************************    ** * ****** ************************************ ******

Astx      CACTAAACCAGAGACTACTACATTACACCCTCTTAGAAGACTTCTAAGATTGTTGTCATGCTTACCACGGACATTCAGATGATGTGTATTCTTGATTGTG 800
Asty1     CACTAAACCAGAAACTACTACATTACACCCTCTTAGAAGACTTCTAAGATTGTTGTCATGCCTACCACAGACATTCAGATGATGTGTATTGTTGATTGTG 342
Asty2     CACTAAACCAGAAACTACTACATTACACCCTCTTAGAAGACTTCTAAGATTGTTGTCATGCCTACCACAGACATTCAGATGATGTATATTGTTGATTGTG 342
          ****************************************************************************************** *********

Astx      ATTTCAAGTTTGGAGCTGATGAAGGAACCCAGGAGATGAACATATGCCTGAGAAGA---ACTAGTGTGGAACTGTCAGCCAGGTTTTTGATGAGTGCTCA 897
Asty1     ATTTCATGTTTGGACCTGATGAAGGAACCCAGGAGATGAACATATGCCTGAGAAGACGAACTAGTGTGGAACTGTCAGCCAGGTTTTTGATGAGTGCTCA 442
Asty2     ATTTCATGTTTGGACCTGATGAAGGA-------------------------------------------------------------------------- 368
          ****** *************************************************   *****************************************

Astx      GACTGCTTCCTGATCCGGCA-CCACATTTACTGCAGTGGACAGATGCTCTAGTCTCTGATGAGGAGTTGCTAGCTGTTAGGAAGTACCTGGAATGGGCTC 996
Asty1     GACTGATTCCTGATCTAGCAACCACATTTACTATAGTGGACAGATGCTCTAGTCTCTGATGAGGAGTTGCTAGCTGTTAGGAAGAACCTGAAATGGGCTC 542
```

```
Asty2    ----------------------------------------------------------------------------------------------------
         ***************  *** ***********  ************************************************** ***** *********

Astx     CACTGCATGAGGTTTGAAGTCTCCAGAGCAGCAAGGAGAGTGGGGAGTAGAACTCATC---TTTAGGGCACTCATCATCGATGGATTGTTCCCAACCTTA 1093
Asty1    CACTGCATGAGGTTTGAAGTCTCCAGAGCAGCAAAGAGAGTGGGGAGTAGAACTCATCATCTTTAGGTCACTCATCATCG-------------------- 622
Asty2    -----------GTTTGAAGTCTCCAGAGCAGCAAAGAGAGTGGGGAGTAGAACTCATCATCTTTAGGTCACTCATCATCG-------------------- 437
         **********************************************************   ****** ************

Astx     GTATACAGACATGGTTGGTTGAACACAAACCTGTCAGGCTGATAAAGCACTAGACATCTACTAACGTCCTTGAAACCATGTTCAGATTGCTGCACATCCT 1193

Astx     CCTGAAGATGCCACGGATGACATGGTTCCTTGTGATTTTTGTTCTCTTCTTGTGTTGCTGTTTTCTTTTAACTTTTGAAGAGGATACTTTATTAGCATGT 1293

Astx     TGCTATGTTAGTCATCTCCTGGCATTAGAAACCAACATCCCACTGCTTAAATGTTTTACATTTCCTACTCTTTTTGGAAAACATAATGAATGCACATCAT 1393

Astx     ATTTTTCCTATTGTTTCACACACATACCACTATGTAATTCAACTTTACAATTGTAATCATTTTGACCAACATTCACAAGAATATAAATTTTATGTGTAAA 1493

Astx     AAA------------------------------------------------------------------------------------------------- 1496
```
