# Supplementary material for: Identification of novel Y chromosome encoded transcripts by testis transcriptome analysis of mice with deletions of the Y chromosome long arm
Source: Genome Biol. 2005 Dec 2;6(12):R102. doi: 10.1186/gb-2005-6-12-r102 (PMC1414076; doi:10.1186/gb-2005-6-12-r102)
Supplement: Additional data file 3 — A diagram providing sequence information on the 'recombinant' loci encoding the transcripts AK016790 and AK015935 [file gb-2005-6-12-r102-S3.pdf]

Additional Data File 3.

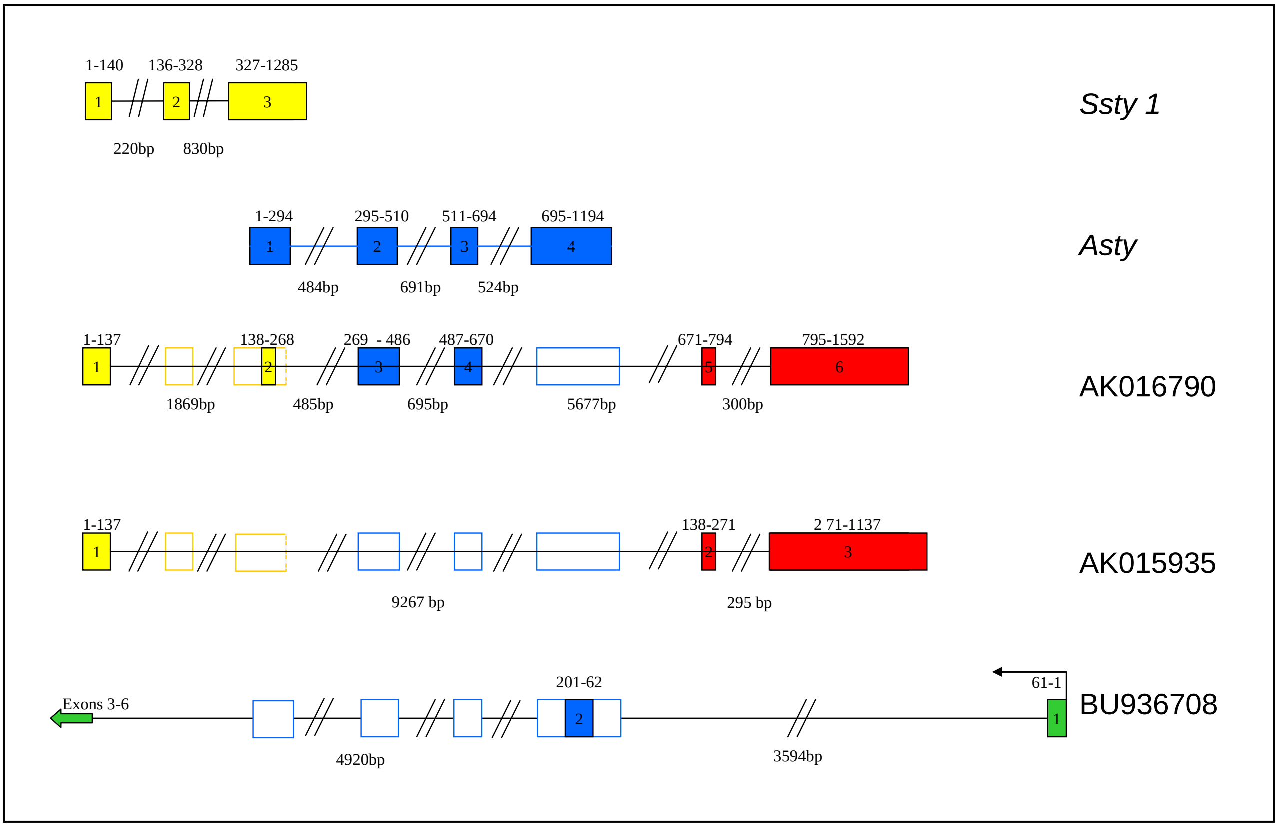

Figure. Diagram of the intron/exon structure of *Ssty1* and *Asty* loci, together with the two structurally identical 'recombinant' loci that, based on sequence matching with BLAST, apparently encode the transcripts AK016790 and AK015935. Also shown is the locus encoding transcript BU936708 that includes part of *Asty* exon 4 transcribed in the antisense direction. The numbers above each locus refer to the base positions of the transcript sequence. The number of base pairs between each transcribed exon is given below each locus. The unfilled coloured outlines show the positions of the *Ssty1* and *Asty* exons in the original loci. In the 'recombinant' loci *Ssty1* exon 3 is incomplete. For details see the clustal alignment below.

CLUSTAL W (1.82) multiple sequence alignment

Ssty1 exon positions   *Asty* exon positions   exons in clones   Homology breakdown after *Asty*

```
                                                                           G
ref|NT_099035.1 (AK016790 locus)    -----TGCACATCTATTCCACTTGGAGGCACTAGAATCCCTAGAAGGTGT 45
ref|NT_078929.2 (AK015935 locus)    ---ATTGCACATCTATTCCACTTGGAGGCACTAGAATCCCTAGAAGGTGT 47
ref|NT_099035.1 (BU936708 locus)    ATCACTCTCTCTCTCTCTCTCTCTCTCTCCCTTCCTTCCTTCCCTCCTTC 50
ref|NT_099026.1 (Ssty1 locus)       ----------------TCCACTTAGAGACACTAGAATGCCTAGAAGGTGT 34
                                                      * **      * **    * * *      *

                                     G
ref|NT_099035.1|MmY_98672_33_5      GTCCACTATTTTCCCAGTGGTGTATGAAAGGCAGGCCAGCTCCTGAACCC 95
ref|NT_078929.2|MmY_78994_33_4      GTCCACTATTTTCCCAGTGGTGTATGAAAGGCAGGCCAGCTCCTGAAGCC 97
ref|NT_099035.1|MmY_98672_33_c      CTTC-CTTTTTTTCTTTTTCTTTCTTTCTCTCTCTCTTTTTTTACTTTC 99
ref|NT_099026.1|MmY_98663_33_c      GTCCACTATTTTCCCAGTGGTCTGTGAAAGGCAAGCCAGCTCCTGAACTC 84
                                     * * ** **** *   *  * * *      *   *    *  *     *

ref|NT_099035.1|MmY_98672_33_5      CAACGTTGTTCCTCAAGTTTTTTTT-GCCTGGTCTGTATCAAAAGACAGA 144
ref|NT_078929.2|MmY_78994_33_4      CAACGTTGTTCCTCAAGTTTTTTTTTGCCTGGTCTGTATCAAAAGACAGA 147
ref|NT_099035.1|MmY_98672_33_c      TTTCTTTCTTACTTTCTTCCCTCCCTCCTTTTTTTCTTTCTTTCTTTCTT 149
ref|NT_099026.1|MmY_98663_33_c      CAAC-TTGTTCCTCAAGCTTTGTG-GCCTGGTCTGTATCAAAAGACAGA 132
                                       * ** ** **        *     * *  * * * **

ref|NT_099035.1|MmY_98672_33_5      ATCTTGATGACTAAGACATTTTCTTTGGGTAAGTACATTTATGAAGTGAA 194
ref|NT_078929.2|MmY_78994_33_4      ATCTTGATGACTAAGACATTTTCTTTGGGTAAGTACATTTATGAAGTGAA 197
ref|NT_099035.1|MmY_98672_33_c      TCTTTCCTTCCTTCAATCTTTCTTTTTCTCTCTCTCGCTTTTTGTCTCTT 199
ref|NT_099026.1|MmY_98663_33_c      ATCTTGACGACTAAGACATTTTCTTTGGGTAAGTACATTTATGAAGTAAC 182
                                       **     **   *  ***  ***         *  ** *    *

ref|NT_099035.1|MmY_98672_33_5      TGGGACCCTCATAGCTATAGAAAGCTGAAAGTTTTCCCTCCCACACTGAG 244
ref|NT_078929.2|MmY_78994_33_4      TGGGACCCTCATAGCTATAGAAAGCTGAAAGTTTTCCCTCCCACACTGAG 247
ref|NT_099035.1|MmY_98672_33_c      TC---TTTCTATCTCTCTCTCTTTCTCGCT-CTCTCCCTCCCTCATTCAC 245
ref|NT_099026.1|MmY_98663_33_c      TGGGACCATCATAGCTATAGAAAGCTGAAAGTTTTCCCTCCCACACTGAG 232
                                    *         **  ** *      **      * ******** ** * *

ref|NT_099035.1|MmY_98672_33_5      TAACTGTACATTCTAAAT-TCTCCCATGTTTGGGGCCAGGGGCATGGTTG 293
ref|NT_078929.2|MmY_78994_33_4      TAACTGTACATTCTAAAT-TCTCCCATGTTTGGGGCCAGGGGCATGGTTG 296
ref|NT_099035.1|MmY_98672_33_c      TTTCTCTCCTTTTTCTTTCTCTTTCTTTCTTTCTCTCTTTCTCTTTCTTT 295
ref|NT_099026.1|MmY_98663_33_c      TAACTGTACAGTCTAAAT-TCTCCCATGTTTGGGGCCAGGGGCATGGTTG 281
                                    *  ** * *  * *   * ***  * *  **     *     * *  **

ref|NT_099035.1|MmY_98672_33_5      ATATAGTT------TAGTTGATCTCCTGAATTTATTATCCTTGCTAACAA 337
ref|NT_078929.2|MmY_78994_33_4      ATCCAGTT------TAGTTGATCTCCTGAATTTATTATCCTTGCTAACAA 340
ref|NT_099035.1|MmY_98672_33_c      CTTTCTTTCTTTCTTTCTTTCTTTCTTTCTTTCTTTCTTTCTTTCTTCCT 345
ref|NT_099026.1|MmY_98663_33_c      ATCCAGTT------TAGTTGATCTCCTGAATTTATTATCCTTGCTAACGA 325
                                     *    **      *  **  * ** *   **  ** *   *     *

ref|NT_099035.1|MmY_98672_33_5      TATCTCCTTCACAATTCCATTAAAATGTCAAAGCTCTTCTTTGTCCGTAT 387
ref|NT_078929.2|MmY_78994_33_4      TATCTCCTTCACAATTCCATTAAAATGTCAAAGCTCTTCTTTGTCCGTAT 390
ref|NT_099035.1|MmY_98672_33_c      TCTTTCCTTCTTTCTTCCCTCCTTAATTTCTTTCTCTTCTTTCCTTCTTT 395
ref|NT_099026.1|MmY_98663_33_c      TATCTCCTTCAAAATTCCATTAAAATGCCAAAGCTCTTCATTGTCCTTAT 375
                                    * * ******    **** *    *        ****** **     * *

ref|NT_099035.1|MmY_98672_33_5      GTATTTTAGAAAATGTTAGTTCTAAATGGCTGATATTGCCTGGACTTGTT 437
ref|NT_078929.2|MmY_78994_33_4      GTATTTTAGAAAATGTTAGTTCTAAATGGCTGATATTGCCTGGACTTGTT 440
ref|NT_099035.1|MmY_98672_33_c      CCTTCTTTCCTTCCTTCCTTCCTTCCTTCCT--CCCTTCCTTCCTTCCTT 443
ref|NT_099026.1|MmY_98663_33_c      GTATTTTAGAAGATGTAACTTCTAAATGGCTGATATTGCCTGGACTTGTT 425
                                       * **        *   * **   *  **     * ***    *  **
```

```
ref|NT_099035.1|MmY_98672_33_5      GAGGCCAGAGTTGCAGTGGCAAATAATCACCAAATAATCCTGAGGCTGGG 487
ref|NT_078929.2|MmY_78994_33_4      GAGGCCAGAGTTGCAGTGGCAAATAATCACCAAATAATCCTGAGGCTGGG 490
ref|NT_099035.1|MmY_98672_33_c      CCTTCCTTCCTTCCTTCATTCCTTTGTTCCTTTCTTTTTTAAGTATTTAT 493
ref|NT_099026.1|MmY_98663_33_c      GAGGCCGGAGTTGCAGTGGCAAATAATCACCAAATAATCCTGAGGCTGGG 475
                                        **    ** *         *  *  *    *  *        *

ref|NT_099035.1|MmY_98672_33_5      TCAGGTGAC-ATAATACAGAAGGCCAGTGGTGTGC-TGTGTCCCTGG--G 533
ref|NT_078929.2|MmY_78994_33_4      TCAGGTGAC-ATAATACAGAAGGCCAGTGGTGTGC-TGTGTCCCTGG--G 536
ref|NT_099035.1|MmY_98672_33_c      TTATTTATTTATTATATATACAGTGTTCTCTATGCATGTGTGCCTGCTCA 543
ref|NT_099026.1|MmY_98663_33_c      TCAGGTAAC-ACAATACAGAAGGCCAGTGGTGTGC-TGTGTCCCTGG--G 521
                                    * *  *    *  *** * *  *       * *** ***** ****

ref|NT_099035.1|MmY_98672_33_5      ATATTTGTGAATATTGCCACCTAATTCAGA-GCTTGCAGGTACAAGAG-- 580
ref|NT_078929.2|MmY_78994_33_4      ATATTTGTGAATATTGCCACCTAATTCAGA-GCTTGCAGGTACAAGAG-- 583
ref|NT_099035.1|MmY_98672_33_c      TTAGAAGAGGGCACTGGATACCATTACAGATGTTTGTGAACTCAGGAGCC 593
ref|NT_099026.1|MmY_98663_33_c      ATATTTGTGAATATTGCCACCTATTTCACA-GCTTGCAGGTACAAGAG-- 568
                                     **   * *   * **    * * * ** * * ***      ** ***

ref|NT_099035.1|MmY_98672_33_5      --AGGGTAGGTGACAAC--TCTGACTCATTTTCTTTATTCT--TTAATCC 624
ref|NT_078929.2|MmY_78994_33_4      --AGGGTAGGTGACAAC--TCTGACTCATTTTCTTTATTCT--TTAATCC 627
ref|NT_099035.1|MmY_98672_33_c      CTGGGAGAGCCGTCAGTGTTCTTAACCACTGAATCATCTCT--CCAGCCC 641
ref|NT_099026.1|MmY_98663_33_c      --AGGGTAGGTGACAAC--TCTGGCTCATTTTCTTTATTCTGTTCTCTCC 614
                                       **  **  * **    ***    ** *   *    ***       **

ref|NT_099035.1|MmY_98672_33_5      TTGATCATTTTG--TAAAAGGCATACCAACTTCCACATTGACATAGACAT 672
ref|NT_078929.2|MmY_78994_33_4      TTGATCATTTTG--TAAAAGGCATACCAACTTCCACATTGACATAGACAT 675
ref|NT_099035.1|MmY_98672_33_c      CAGGTACTTTCT--TTTTAAGCAAAACGGGT-CCAGAAGGTCATAGAAGT 688
ref|NT_099026.1|MmY_98663_33_c      TTGATCATTTTGGGTAAAAGGCATACCAACTTCCACATTGACATAGACAT 664
                                      * *  ***    *   * *** * *   * *** *  * ******  *

ref|NT_099035.1|MmY_98672_33_5      GCAAGGAGATGTTCCTCAGCAGAGGCAAATTAGCTCAGGGCTGAAGACAT 722
ref|NT_078929.2|MmY_78994_33_4      GCAAGGAGATGTTCCTCAGAAGAGGCAAATTAGCTCAGGGCTGAAGACAT 725
ref|NT_099035.1|MmY_98672_33_c      -TGCAGATATGAACACATGCTACTCAACCTGTTTTCAAGGTATCTCACAC 737
ref|NT_099026.1|MmY_98663_33_c      GCAAGGAGATGTTCCTCAGCAGAGGCAAATTAGCTCAGGGCTGAAGACAT 714
                                         ** ***  *    *       *  *    *** **      ***

ref|NT_099035.1|MmY_98672_33_5      ATTCATCTGAGTATGTTA--TTCTGAGCTCTTGAGAATTACTTGTTAATT 770
ref|NT_078929.2|MmY_78994_33_4      ATTCATCTGAGTATGTTA--TTCTGAGCTCTCGAGAATTACTTGTTAATT 773
ref|NT_099035.1|MmY_98672_33_c      CATCACCTTTGGATGAAGGGTCATCAGATTTTCACTTGGGATAGGTGGCT 787
ref|NT_099026.1|MmY_98663_33_c      ATTCATCTGAGTATATTA--TTCTGAGCTCTCGAGAATTACTTGTTAATT 762
                                      *** **  * **      *  * ** * *  *       * * *   *

ref|NT_099035.1|MmY_98672_33_5      TCCCTTCGATAATTTCCTTGTTTCCCTTATTTATTTATTTATTTATTTAT 820
ref|NT_078929.2|MmY_78994_33_4      TCCCTTCGATAATTTCCTTGTTTCCCTTATTTATTTATTTATTTATTTAT 823
ref|NT_099035.1|MmY_98672_33_c      TTCATGACCTACTGCTTTTACCAACAATACCCAAAACCTTCCATTTCTGA 837
ref|NT_099026.1|MmY_98663_33_c      TCCCTTTGATAATTTCCTTGTTTCC------------------------- 787
                                    * * *    ** *    **     *

ref|NT_099035.1|MmY_98672_33_5      TTATTTATTTATTTATTTATTTATTTATTTATTTTTTTATTTTTTGTTTTGTTT 870
ref|NT_078929.2|MmY_78994_33_4      TTATTTATTTATTTATTTATTTATTTATTTATTTATTTATT-TATTGTTT 872
ref|NT_099035.1|MmY_98672_33_c      CCAAGGTCAGTTATAATTATGACTGGAGTTTACTATTGGAATCTCAGAAT 887
ref|NT_099026.1|MmY_98663_33_c      -----------TTTATTTATTTGTTTGTTGTTGTTGTTGTTGTTTTGTTT 826
                                               * ** ****   *    *    *  *         *  *

ref|NT_099035.1|MmY_98672_33_5      GTTCATTTTTTCTTTGGATGGGTGATTAATTTTAATTTGAGTGTGTTTTT 920
ref|NT_078929.2|MmY_78994_33_4      GTTCATTTTTTCTTTGGATGGGTGATTAATTTTAATTTGAGTGTGTTTTT 922
ref|NT_099035.1|MmY_98672_33_c      TTTCATTTTTCCCAAGGGTACCAAAATCCAGGTATCCTAAGAGCATCCCT 937
ref|NT_099026.1|MmY_98663_33_c      GTTTGTTTTTTCTTTGGATGGGTGTTTAATTTTACTTTGAGTGTGTTTGT 876
                                     **   ***** *   ** *       *     **   * ** *  *   *
```

```
ref|NT_099035.1|MmY_98672_33_5      GTGTAGCTTTGGCTGTTAGGAAAATCACTCTGTAGAATAG-ACTAGCCTT 969
ref|NT_078929.2|MmY_78994_33_4      GTGTAGCTTTGGCTGTTAGGAAAATCACTCTGTAGAATAG-ACTAGCCTT 971
ref|NT_099035.1|MmY_98672_33_c      ACATACATATGGGCCTCAAATATAACACTCCATTCGTGAGCGTTAGATCA 987
ref|NT_099026.1|MmY_98663_33_c      GTGTAGCTTTGGCTGTTAGGAAAATCACTTTGTAGAATAG-ACTAGCCTT 925
                                       **  * ***   * *   * * ****   *     **   ***

ref|NT_099035.1|MmY_98672_33_5      TATCTCACTAAGGTTTGCCTCC------CTCTGCCTC-TTGAGTACTAGG 1012
ref|NT_078929.2|MmY_78994_33_4      TATCTCACTAAGGTTTGCCTCC------CTCTGCCTC-TTGAGTACTAGG 1014
ref|NT_099035.1|MmY_98672_33_c      TTTCTCTCCAAACATGGTCTCTAGGAAATTATACTTCACTTAATTCTGCC 1037
ref|NT_099026.1|MmY_98663_33_c      TATCTCACTAAGGTTTGCCTCC------CTCTGCCTC-TTGAGTACTAGG 968
                                    * **** * **   * * ***        * * * **  * * * **

ref|NT_099035.1|MmY_98672_33_5      ATTTAAGACTTGGGTCACTAACACCTCCTATTAACTTCTGATTTGATTGT 1062
ref|NT_078929.2|MmY_78994_33_4      ATTTAAGACTTGGGTCACTAACACCTCCTATTAACTTCTGATTTGATTGT 1064
ref|NT_099035.1|MmY_98672_33_c      CTGCATTAACAGAGCCTGAAACATAGGTCATCTATCCTTCCTACCATCCT 1087
ref|NT_099026.1|MmY_98663_33_c      ATTAAAGATTTGGGTTACTACCATCTCCTATTAACTTCTGATTTGATTGT 1018
                                     *  *  *   * *     * **      **   *    *  *   **  *

ref|NT_099035.1|MmY_98672_33_5      TTGTTTTGTTTTTTGTTTGTTTT-TCATTTTTGTTTTTTTTGTTTTGTTT 1111
ref|NT_078929.2|MmY_78994_33_4      TTGTTTTGTTTTTTGTTTGTTTT-TCATTTTTTTTTGTTTTGTTT--GTT 1111
ref|NT_099035.1|MmY_98672_33_c      TCATGCAAACACCCATATAACTGATGATAAGCTTCAAGAGTGCTTAGAGA 1137
ref|NT_099026.1|MmY_98663_33_c      TTGTTTTGTTTTGT-TCTGTTTG------TTTTTCTTTTTTGATT---TT 1058
                                    *  *           * *   *           *      ** **

ref|NT_099035.1|MmY_98672_33_5      TGTTTGTTTGTTTGTTTGTTTTTTGTTGTTGTTTTTTTGTTTAAGTTGTT 1161
ref|NT_078929.2|MmY_78994_33_4      TGTTTGTTTGTTTGTTT-TTTGTTGTTGTTGTTTTTTTGTTTAAGTTGTT 1160
ref|NT_099035.1|MmY_98672_33_c      CTTACATTCATGC-CTGATGCATAATGCTTGTATTGCTGCCAGGCTTACC 1186
ref|NT_099026.1|MmY_98663_33_c      TGTTTGTATGTTT--TGTTTTGTTTTTGTTGGTTTTTTGTTTTAGTTGTT 1106
                                      *   *   *    *  *   *  *  ***  **  **      **

ref|NT_099035.1|MmY_98672_33_5      AATGTTGTTGCTGATTTGGAGACATGGTTCCTTTGTCTACCACTGGCTGC 1211
ref|NT_078929.2|MmY_78994_33_4      AATGTTGTTGCTGATTTGGAGACATGGTTCCTTTGTCTACCACTGGCTGC 1210
ref|NT_099035.1|MmY_98672_33_c      AAGGTGTATGTGAAGATGG-GACTCTTCTACGTTTTGAAGCAGTTCTACC 1235
ref|NT_099026.1|MmY_98663_33_c      AATGTTGTAGCTGATTTGGAGACATTTTTCTTTGTCTATCACTGGCTGC 1156
                                    ** **    *   *  *** ***     * * ** *  * ** *     *

ref|NT_099035.1|MmY_98672_33_5      CCTGGAACTCACTTTGTAGACCAGGCTGGTCTCTAAGTC----AGAAATC 1257
ref|NT_078929.2|MmY_78994_33_4      CCTGGAACTCACTTTGTAGACCAGGCTGGTCTCTAAGTC----AGAAATC 1256
ref|NT_099035.1|MmY_98672_33_c      TCTCCTGCTTAAG--GTAGGCTAGGTCACTCCCTTAACCCTAAAGAGGTT 1283
ref|NT_099026.1|MmY_98663_33_c      CCTGGAACTCACTTTGTAGACCAGGCTGGTCTCTAAGTC----AGAAATC 1202
                                     **    ** *    **** * ***    ** ** *  *    ***  *

ref|NT_099035.1|MmY_98672_33_5      TGCCTTATCCTGCCTTCTGAATGCTGAGTTTAAATGCTTGTGCCACCATA 1307
ref|NT_078929.2|MmY_78994_33_4      TGCCTTATCCTGCCTTCTGAATGCTGAGTTTAAATGCTTGTGCCACCATA 1306
ref|NT_099035.1|MmY_98672_33_c      GGGGACATTAAGCAGACTCTAATTCTGCTTTAATCTTCTACTCCTAAACA 1333
ref|NT_099026.1|MmY_98663_33_c      TGCCTGACCCTGCCTTCTGAAAGCTGGGATTAAATGTGTGTGCCACTATA 1252
                                     *    *    **   **  *        ****      *   **   * *

ref|NT_099035.1|MmY_98672_33_5      GCTGGATTATGAACTTCTTATTTGGATCAAATTAAGTTTAGAAGCCCAAA 1357
ref|NT_078929.2|MmY_78994_33_4      GCTGGATTATGAACTTCTTATTTGGATCAAATTAAGTTTAGAAGCCCAAA 1356
ref|NT_099035.1|MmY_98672_33_c      TCAATACT-TAGACCACTAATTTGTGTCCATTATATATCAAATGCACCCT 1382
ref|NT_099026.1|MmY_98663_33_c      GCTGGATTATGAGCTTCTTATTTTGATTAAATTAAGTTTAGAAGCCCAAA 1302
                                     *   * * *   *  ** ****   *  * *  *  * * * ** *

ref|NT_099035.1|MmY_98672_33_5      CATGTTTAGCATAGCCTTCTTGACCTACTCAAAAATTCCATTAGAGAAGA 1407
ref|NT_078929.2|MmY_78994_33_4      CATGTTTAGCATAGCCTTCTTGACCTACTCAAAAATTCCATTAGAGAAGA 1406
ref|NT_099035.1|MmY_98672_33_c      C-TGTATACATGAGGGTCCAATATCTGTCTTGTAAGCTTGATGGGCCTGA 1431
ref|NT_099026.1|MmY_98663_33_c      CATGTTTAGCATAACCTTCTTGACCTGCTCAAAAATTCCATTAGAGAAGA 1352
                                    * *** **    *   * *   * **       **      * *    **
```

```
ref|NT_099035.1|MmY_98672_33_5      GCATTTCTCCAGCATTCTCTCCCATCCTACACTCATTGCCTTTCAGTAAC 1457
ref|NT_078929.2|MmY_78994_33_4      GCATTTCTCCAGCATTCTCTCCCATCCTACACTCATTGCCTTTCAGTAAC 1456
ref|NT_099035.1|MmY_98672_33_c      GATCATCTCTACTTGCCCCAGGCCCCAAACTTCACTTCCAGTCCAAAAGG 1481
ref|NT_099026.1|MmY_98663_33_c      GCCTTGGTCCTGCATTCTCTCCCACCCTACACCCATTGCCTTTCAGTAAC 1402
                                    *      **       * *   *  *  **     ** *  * **  *

ref|NT_099035.1|MmY_98672_33_5      TCATATATA--------GCAACAGCATTGTGTCTGGTGTAAACAGGCAAT 1499
ref|NT_078929.2|MmY_78994_33_4      TCATATATATGACAGTAGCAACAGCATTGTGTCTGGTGTAAACAGGCAAT 1506
ref|NT_099035.1|MmY_98672_33_c      TCTTCAATA--------ATTACCCTCTCTGACCAGAAAGAATCACAAACT 1523
ref|NT_099026.1|MmY_98663_33_c      TCATATATATGACAGTAGCAACAGCATTGTGTCTGGTTTAAACAGGCAAT 1452
                                    ** *  ***           **    *     * *    ** **   * *

ref|NT_099035.1|MmY_98672_33_5      ACCCTCAATCCAAGGCAATGGGCAAGTATGAT-ATTTGAACACAGAATGA 1548
ref|NT_078929.2|MmY_78994_33_4      ACCTTCAATCCAAGGCAATGGGCAAGTATGAT-ATTTGAACACAGAATGA 1555
ref|NT_099035.1|MmY_98672_33_c      ATTCTCTATCTGA--TAGAGGGCAAAAATTTCCATTTAACTGTGTCTCAC 1571
ref|NT_099026.1|MmY_98663_33_c      ATCTTCAATCCAAGGCAATGGGCAAGTAT----ATTTGAACACAGAATGA 1498
                                    *   ** ***  *   *  ******  **    **** *

ref|NT_099035.1|MmY_98672_33_5      TACCCACAAGACATCTGCCCAGGGTTGGTGTGGTCTACCACACCAGGGCT 1598
ref|NT_078929.2|MmY_78994_33_4      TAACCACAAGACATCTGCCCAGGGTTGGTGTGGTCTACCACACCAGGGCT 1605
ref|NT_099035.1|MmY_98672_33_c      TTCGAATTCCTCATTTGAA-AGGGGCACCAGAATGAATCTTTTTGTTCCA 1620
ref|NT_099026.1|MmY_98663_33_c      TAGCCACAAGACATCTGCCTGGGTTTGGTGTGGTATACCACAGCAAGGCT 1548
                                    *    *     *** **    **          *  * *         *

ref|NT_099035.1|MmY_98672_33_5      ACAGCCATGTAGAAGACAATACCTCT-GAAGAGTCTTAGCATGACATCCC 1647
ref|NT_078929.2|MmY_78994_33_4      ACAGCCATGTAGAAGACAATACCTCT-GAAGAGTCTTAGCATGACATCCC 1654
ref|NT_099035.1|MmY_98672_33_c      GCAGTTCTCAAATTTACATCCTCTGTCGAAGAGGTCTATAA--ATGTCAT 1668
ref|NT_099026.1|MmY_98663_33_c      ACAGCCATGTAGAAGAAAACACCTTT-GAAGAGGCTTAGCATGTCATCCC 1597
                                     ***   *  *    * *    ** * ******   **  *     **

ref|NT_099035.1|MmY_98672_33_5      TCATGAAGAAGAAGAGGAGGATGTCTTCTTCCAACGTCTTGAGGAATATT 1697
ref|NT_078929.2|MmY_78994_33_4      TCATGAAGAAAAAGAGGAGGATGTCTTCTTCCAACGTCTTGAGGAATATT 1704
ref|NT_099035.1|MmY_98672_33_c      CCAGTCATACCAGGGCTTCAGTGCCT-CTCCCTCTCCCTATGGGAGCCTC 1717
ref|NT_099026.1|MmY_98663_33_c      TCATGAAGAAGAGGAGGAGGAAGTCTTCTTCCAACACCCTGAGGAATATT 1647
                                     **   * *  * *        * ** ** **     *    ***   *

ref|NT_099035.1|MmY_98672_33_5      GTTGGCTGCAGAATT--TCTCAAGGTTGGAAGGAAGGTAATGAGCCTGTC 1745
ref|NT_078929.2|MmY_78994_33_4      GTTGGCTGCAGAATT--TCTCAAGGTTGGAAGGAAGGTAATGAGCCTGTC 1752
ref|NT_099035.1|MmY_98672_33_c      AGACACACCACATCTAGTCATAAATATTTCCTCTTCTCCCTGTGTCTATT 1767
ref|NT_099026.1|MmY_98663_33_c      GTCAGCTGCAGAATT--TCTCACAGTTGGAAGGAAGGTAATGAGCCTGTC 1695
                                         *  ** *  *  **  *    *             ** * ** *

ref|NT_099035.1|MmY_98672_33_5      ACTCAATGGAAGACCATAG----TTCTAGGTCAACTGCCAACAAACCCTT 1791
ref|NT_078929.2|MmY_78994_33_4      ACTCAATGGAAGACCATAG----TTCTAGGTCAACTGCCAACAAACCCTT 1798
ref|NT_099035.1|MmY_98672_33_c      GGGCAACAAAGCCTTCTCGAATGCCCTTATTGGACTGA-AATATTTCCTT 1816
ref|NT_099026.1|MmY_98663_33_c      ACCCAATGGAAGGCCATAG----TTCTAGATCAACTGCCAACAAACCCTT 1741
                                       ***   *      * *      **   *  ****  ** *   ****

ref|NT_099035.1|MmY_98672_33_5      CTCTTTATTTGGTGAAGTATGATGGAAATGACAGCGTCTATGGACAGGTG 1841
ref|NT_078929.2|MmY_78994_33_4      CTCTTTATTTGGTGAAGTATGATGGAAATGACAGCGTCTATGGACAGGAG 1848
ref|NT_099035.1|MmY_98672_33_c      TTTTTTTTTCAGTGGTG---GACACATACC-TACCCTCTCTTGTAAAGTG 1862
ref|NT_099026.1|MmY_98663_33_c      CTCTTTACTTGGTGAAGTATGATGGAATTGACAGCATCTACGTACTGGAG 1791
                                     * ***  *  ***  *   **   *      * * ***        * *

ref|NT_099035.1|MmY_98672_33_5      CTCTACAATGATGACAGGATTTTAAACCTTAAGGTTTTGCCTCCCAAAGT 1891
ref|NT_078929.2|MmY_78994_33_4      CTCTACAATGATGACAGGATTTTAAACCTTAAGGTTTTGCCTCCCAAAGT 1898
ref|NT_099035.1|MmY_98672_33_c      T----AAATAGATCTTGTCTTATTGACTTTG----TGTGCCTGCTATATT 1904
ref|NT_099026.1|MmY_98663_33_c      CTCTACAGTGATGACAGGATTTTAAACCTTAAGGTTTTGCCTCCCATAGT 1841
                                          * *       *  ** *  ** **     * ***** * * * *
```

```
ref|NT_099035.1|MmY_98672_33_5      AAGATTTCCTCAGGTAAGGGATGCCCACCTCGCCAGAGCCCTGGTTGGCA 1941
ref|NT_078929.2|MmY_78994_33_4      AAGATTTCCTCAGGTAAGGGATGCCCACCTCGCCAGAGCCCTGGTTGGCA 1948
ref|NT_099035.1|MmY_98672_33_c      CCAACTGTCTAAGATTAC--TTCCAAACTTCTTCCTGTTACAAATATTCG 1952
ref|NT_099026.1|MmY_98663_33_c      AGTATTTCCTCAGGTGAGGGATGCCCACCTCGCCAGAGCCCTGGTTGGCA 1891
                                       * *  ** ** * *    * *  ** **  *      *   *   *

ref|NT_099035.1|MmY_98672_33_5      GAGCGGTACAACACAAATTTAAGGGTAAAGATGGCTCTGAGGACAACTGG 1991
ref|NT_078929.2|MmY_78994_33_4      GAGCGGTACAACACAAATTTAAGGGTAAAGATGGCTCTGAGGACAACTGG 1998
ref|NT_099035.1|MmY_98672_33_c      GAGACATTCCTC-CATTCAAGAAAATTGGCCTTGCTCTTATGTCATCTGA 2001
ref|NT_099026.1|MmY_98663_33_c      GAGCGGTACAACACAAATTTGAGAGGAAAGATGGCTCTGAGGTCAACTGG 1941
                                    ***   * *  * **      *         * ***** * * ** ***

ref|NT_099035.1|MmY_98672_33_5      AGGAGGGTTATGCTAGCCCAGGTGCCAATCATGAAGGATTTGTTTTACAG 2041
ref|NT_078929.2|MmY_78994_33_4      AGGAGGGTTGTGCTAGCCCAGGTGCCAATCATGAAGGATTTGTTTTACAG 2048
ref|NT_099035.1|MmY_98672_33_c      CTAAAGGTT-TCATAGACCAAA-GATAACCATCCA--ACCTGT--TGGAA 2045
ref|NT_099026.1|MmY_98663_33_c      AGGGGGGTGGTGCTAGCCCAGGTGCCAATCATGAAGGATTTGTTTTACAT 1991
                                         ***  *  *** ***   *  **  ***  *  *  ***  *  *

ref|NT_099035.1|MmY_98672_33_5      TAC-TACAAGAAGAATCCAGCTCTCTACTTCTATCAGCTCCTGGATGACT 2090
ref|NT_078929.2|MmY_78994_33_4      TACCTACAAGAAGGATCCAGCTCTCTACTTCTATCAGCTCCTGGATGACT 2098
ref|NT_099035.1|MmY_98672_33_c      TAC---TGAAACAATTGCTGCCTGCT--TTCCACATACCACATAAAATAT 2090
ref|NT_099026.1|MmY_98663_33_c      TACCTACAAGAAGGATCCAGCTCTCTATGCTTATCAGCTCCTGGATGACT 2041
                                    ***     * *    * * **   **      *    *  *   *    *

ref|NT_099035.1|MmY_98672_33_5      ACAAGAAAGGGAACCTCCACATCATTCCAGACACTCCTCTG-GCTGAGGA 2139
ref|NT_078929.2|MmY_78994_33_4      ACAAGAAAGGGAACCTCCACATCATTCCAGAAACTCCTCTG-GCTGAGGA 2147
ref|NT_099035.1|MmY_98672_33_c      GTGGATATCCTAAATTCTGCAAAAAAAAAAAAAAGATTTGTAAGCTGATCA 2140
ref|NT_099026.1|MmY_98663_33_c      ACAAGGAAGGTAACCTCCACATGATTCCAGACACTCCTCCG-GCTGAGGA 2090
                                          *    **  **  **  *    * * *    *    *****  *

ref|NT_099035.1|MmY_98672_33_5      AAGATCAGGAGATGACAGTGATGTGTTGATAGGTAACTGGGTGCAGTACA 2189
ref|NT_078929.2|MmY_78994_33_4      GAGATCAGGAGATGACAGTGATGTGTTGATAGGTAACTGGGTGCAGTACA 2197
ref|NT_099035.1|MmY_98672_33_c      AGCAATACTGTGACTCCCTCTTCTCCAGA-AGGTATTTGCATTTTCACCA 2189
ref|NT_099026.1|MmY_98663_33_c      GAGATCAGGAGATGACAGTGATGTGTTGATTGGTAACTGGGTGGAGTACA 2140
                                       *  *        *  *  * *   **  ****  **  *      **

ref|NT_099035.1|MmY_98672_33_5      CCA-GAAAAGGTGGTTCCAAATAGTTCAGAAAGGTTGTTTACCAAATTCT 2238
ref|NT_078929.2|MmY_78994_33_4      CCA-GAAAAGGTGGTTCCAAATAGTTCAGAAAGGTTGTTTACCAAATTCT 2246
ref|NT_099035.1|MmY_98672_33_c      ATGTGAAATGGTATTTCTTATTTATTGAAAATAGCCAGAGATTTTACACT 2239
ref|NT_099026.1|MmY_98663_33_c      CCA-GAAAAGATGGTTCCAAAAAGTTCGGAAAGGTTGTTTACCAAGTTCT 2189
                                        **** * *  ***  *    **   **  *      *       **

ref|NT_099035.1|MmY_98672_33_5      AGCCAATCCTTC-ACATTCAATTTCACATTTGCAAAGTGTCACAGAAATG 2287
ref|NT_078929.2|MmY_78994_33_4      AGCCAATCCTTC-ACATTCAATTTCACATTTGCAAAGTGTCACAGAAATG 2295
ref|NT_099035.1|MmY_98672_33_c      TTTTACAAGTAG-GTTTAGAATTGCACATTTGCAAAGTGTCACAGAAATG 2288
ref|NT_099026.1|MmY_98663_33_c      AGCCAATCCTTCCGTGTACTTTATCAAGTTT-CATGGTGACATCCATATC 2238
                                        *    *      *    *  **  *** **  *** **   * **

ref|NT_099035.1|MmY_98672_33_5      C-TGCTTATGACCAGAGTGCTTAAACTTCCTTATTCT-------CATGAA 2329
ref|NT_078929.2|MmY_78994_33_4      C-TGCTTATGACCAGAGTGCTTAAACTTCCTTATTCT-------CATGAA 2337
ref|NT_099035.1|MmY_98672_33_c      C-TGCTTATGACCAGAATGCTTAAACTTCCTTATTCT-------CATGAA 2330
ref|NT_099026.1|MmY_98663_33_c      TATGTCTAT-ACTATGGTGCCAAAGATTCTTGAAGTTGAAAAATCATAAA 2287
                                      **  *** ** *   ***  **  *** * *   *       *** **

ref|NT_099035.1|MmY_98672_33_5      GTTT-GTCACATTACCTC------CTTGATGAAGGGCACAACCATCCTAC 2372
ref|NT_078929.2|MmY_78994_33_4      GTTT-GTCACATTACCTC------CTTGATGAAGGGCACAACCATCCTAC 2380
ref|NT_099035.1|MmY_98672_33_c      GTTT-GCCACATTACCTC------TTT-ATGAAGGGCACAACCATCCTAC 2372
ref|NT_099026.1|MmY_98663_33_c      GTACAGAAACGTAAACATATAGGACTGGAAGAAAAAAAAAGTTTTTTTTT 2337
                                    **   *  ** * * *         *  * ***    * *    *  *
```

```
ref|NT_099035.1|MmY_98672_33_5      TTCTGTG-------CAAAATTGCCCAAGCTCCTCCCC-TATCTTACTT-- 2412
ref|NT_078929.2|MmY_78994_33_4      TTCTGTG-------CAAAATTGCCCAAGCTCCTCCCC-TATCTTACTT-- 2420
ref|NT_099035.1|MmY_98672_33_c      TTCTGTG-------CAAAACTGCCCAAGCTCCTCCCC-TATCTTACTT-- 2412
ref|NT_099026.1|MmY_98663_33_c      TCCTGTGTTGGCTACATAAGGGTCTTTGATAATCCCAGTATCTTTGCCAA 2387
                                    * *****       ** **  * *   * *  ****  ******

ref|NT_099035.1|MmY_98672_33_5      TAGGAAGATTTAGACATTCACAACAGAGA---GTACTCCAACCTGTGTTG 2459
ref|NT_078929.2|MmY_78994_33_4      TAGGAAGATTTAGACATTCACAACAGAGA---GTACTCCAACCTGTGTTG 2467
ref|NT_099035.1|MmY_98672_33_c      TAGGAAGATTTATACATTCACAACAGAGA---GTGCTCCAACCTGTGTGG 2459
ref|NT_099026.1|MmY_98663_33_c      TAAAATGTGTTTTGTTCTAAAAATGTAAATGTGTGACATGACATGCTGTG 2437
                                    **  * *  **      * * **   * *   **      ** **    *

ref|NT_099035.1|MmY_98672_33_5      AGC--ACAGTGTGTTTGAGGAAATCCCCCAGATTC--ACCATCTTCCCA- 2504
ref|NT_078929.2|MmY_78994_33_4      AGC--ACAGTGTGTTTGAGGAAATCCCCCAGATTC--ACCATCTTCCCA- 2512
ref|NT_099035.1|MmY_98672_33_c      AGC--ACAGTGTGTTTGAGGAAATCCCCCAGATTC--TCCATCTTCCCA- 2504
ref|NT_099026.1|MmY_98663_33_c      AGTGAACAATTTGCTGGACGAGATGGCCTACAGTGGAAAGAACTTCAAAT 2487
                                    **   *** * ** * ** ** **  ** * * *      * ****  *

ref|NT_099035.1|MmY_98672_33_5      ----GTGTATGTCCTACCTATAACTTCTAATT-TCCATGGTCTGGGAAGC 2549
ref|NT_078929.2|MmY_78994_33_4      ----GTGTATGTCCTACCTATAACTTCTAATT-TCCATGGTCTGGGAAGC 2557
ref|NT_099035.1|MmY_98672_33_c      ----GTGTATGTCCTACTTATAACTTCTAATT-TCCGTGGTCTGGAAAGC 2549
ref|NT_099026.1|MmY_98663_33_c      GAAGATGAAAGTTTCATTTCAGGCGGTGAACAGTACATACACCTAAAATC 2537
                                         ** * **   *  *    *    **   * * *   *    ** *

ref|NT_099035.1|MmY_98672_33_5      AC-TGTTCTGATCTAAGAGATTTCTGAACACTCTGGACTGAAATCCCAAA 2598
ref|NT_078929.2|MmY_78994_33_4      AC-TGTTCTGACCTAAGAGATTTCTGAACACTCTGGACTGAAATCCCAAA 2606
ref|NT_099035.1|MmY_98672_33_c      AC-TGTTCTGGCCTAAGAGATTTCTGAACACTCTGGACTGAAATCCCAAA 2598
ref|NT_099026.1|MmY_98663_33_c      ATACATGCTAAAATGAACAGCTTCTCTGGTCTGCATTCCGAATAAGGAAT 2587
                                    *    * **    * *     ****     **     * ***     **

ref|NT_099035.1|MmY_98672_33_5      CAGAA---GAGTATCAAGGTAAGGGTAAATCAGATTACTATCTTTGTTTT 2645
ref|NT_078929.2|MmY_78994_33_4      CAGAA---GAGTATCAAGGTAAGGGTAAATCAGATTACTATCTTTGTTTT 2653
ref|NT_099035.1|MmY_98672_33_c      CAGAA---GAGTATCAAGGTAAGGGTAAATCAGTTTACTATCTTTGTTTT 2645
ref|NT_099026.1|MmY_98663_33_c      TGGAGATGGAACTTTGAGGAGCAGGGATGGCAGAAAAAGATGGATGTTTA 2637
                                      **    **   *  ***    ** *   ***   *  **   *****

ref|NT_099035.1|MmY_98672_33_5      GT---TACCTTAGACAGAGACATCATGGCC----TCCATGCAGAGGAATG 2688
ref|NT_078929.2|MmY_78994_33_4      GT---TACCTTAGAGAGAGACATCATGGCC----TCCATGCAGAGGAATG 2696
ref|NT_099035.1|MmY_98672_33_c      AT---TACCTTAAACAGAGACATCCTGGCC----TCCATGCAGATGAATG 2688
ref|NT_099026.1|MmY_98663_33_c      GTAAGTAGGGGAGAAGGACAGGACATAGGTAGAACCTCTGATGTGAAGAC 2687
                                     *   **    * *  ** *   * * *       *  **  *   *

ref|NT_099035.1|MmY_98672_33_5      TGTGGATGGACATATATTTAAGGAAGACACTAAACCAGAAAATACCACAT 2738
ref|NT_078929.2|MmY_78994_33_4      TGTGGATGGACATATATTTAAGGAAGACACTAAACCAGAAAATACCACAT 2746
ref|NT_099035.1|MmY_98672_33_c      TGTGGATGGACATATATTTAAGGAAGACACTAAACCAGAAAATACTACAT 2738
ref|NT_099026.1|MmY_98663_33_c      TGAAGGAGAAACAAAATTTGGGGAAAGTGGGACATTTAAAATTAATGTG- 2736
                                    **  *  * *   * ****  ****      * *    *** **

ref|NT_099035.1|MmY_98672_33_5      TACACCCTCTTAAAAGACTTCTAAGATTGTTGTCATGCCTACCACAGACA 2788
ref|NT_078929.2|MmY_78994_33_4      TACACCCTCTTAAAAGACTTCTAAGATTGTTGTCATGCCTACCACAGACA 2796
ref|NT_099035.1|MmY_98672_33_c      TGCACCCTCTTAAAAAACTTCTAAGATTGTTGTCATGACTACCACTGAAA 2788
ref|NT_099026.1|MmY_98663_33_c      ---GCCTTGTTGCACAAGGTGAAACCTAGAAAAC-TGATTGAAATAGAC- 2781
                                        ** * **  *  *  *  **  * *    * **  *   *  **

ref|NT_099035.1|MmY_98672_33_5      TTCAGATGATGTGTATTCTTGATTGTGATTTCATGTTTGGAGCTGATGAA 2838
ref|NT_078929.2|MmY_78994_33_4      TTCAGATGATGTGTATTCTTGATTGTGATTTCATGTTTGGAGCTGATGAA 2846
ref|NT_099035.1|MmY_98672_33_c      TTCAGATGATGTGTATTCTTGATTGTGATTTCATGTTTGGAGCTGATGAA 2838
ref|NT_099026.1|MmY_98663_33_c      -TCAGAAATCAGAGAAATCAGGTTTTCTTTGAATAATGACACTTAAAGAA 2830
                                     *****        *     * ** *  **  **  *   *  * * ***
```

```
ref|NT_099035.1|MmY_98672_33_5      GGAGAAAGTACCTGGAATGTCCTAGAATTCTCAAG-GTATCAATCAGTGT 2887
ref|NT_078929.2|MmY_78994_33_4      GGAGAAAGTACCTGGAATGTCCTAGAATTCTCAAG-GTATCAATCAGTGT 2895
ref|NT_099035.1|MmY_98672_33_c      GGAGTAAGTACCTGGAATGTCCTATAATTCTCAAG-GTATCAATCAGTGT 2887
ref|NT_099026.1|MmY_98663_33_c      ACCTGTCTTACATTTTAAGATTTATTATCTTTACTTATGTGGGTGGGGAT 2880
                                            *** *   * *   **  **  * *    * *   *  *  *

ref|NT_099035.1|MmY_98672_33_5      TCAATTTCTGCTCAATAGTGCTG-CAGACATAGGAATA-ACAGGATAAAA 2935
ref|NT_078929.2|MmY_78994_33_4      TCAATTTCTGCTCAATAGTGCTG-CAGACATAGGAATA-ACAGGATAAAA 2943
ref|NT_099035.1|MmY_98672_33_c      TCAATTTCTGATCAATAGTGCTG-CAGACATAGGAATA-ACAGGATAAAA 2935
ref|NT_099026.1|MmY_98663_33_c      ATGGTATGTGCTTTTGAGTACAGGTGAACACAGAGACATGCATTGTCAGG 2930
                                        * * ** *    *** * *    *** **  * *  **   * *

ref|NT_099035.1|MmY_98672_33_5      CTATTCTATACAATCCTATGAAGCTCCTATC-ACCATC--CTCCTATTTT 2982
ref|NT_078929.2|MmY_78994_33_4      CTATTCTATACAATCCTATGAAGCTCCTATC-ACCATC--CTCCTATTTT 2990
ref|NT_099035.1|MmY_98672_33_c      CTATTCTATACAATCCTATGAAGCTCCTATC-ACCATC--ATCCTATTTT 2982
ref|NT_099026.1|MmY_98663_33_c      TCCCCTGGAGCTGACATTAAAGGTGATTATTTGCCATTGGATATGAATTC 2980
                                              *   * *   * *    ***   ****    *   * **

ref|NT_099035.1|MmY_98672_33_5      AGAGAATCTTAGATCTATCACTAATCCTGAGACAGGCAGAAACAATTCTT 3032
ref|NT_078929.2|MmY_78994_33_4      AGAGAATCTTAGATCTATCACTAATCCTGAGACAGGCAGAAACAATTCTT 3040
ref|NT_099035.1|MmY_98672_33_c      AGAGAATCTTAGCTCTATCACAAATCCCGAGACCAGCAGAAACAATTCTT 3032
ref|NT_099026.1|MmY_98663_33_c      AAGAAACC--AAATCCATTTT---TCTTGTGGT--GCAAAAGTGCTTGTT 3023
                                    *   ** *  *  ** **      **  * *    *** **    ** **

ref|NT_099035.1|MmY_98672_33_5      CTCCTGCACATAGGAGT---ACTGCATATGTCTCCCCTTACCCAAAGTAT 3079
ref|NT_078929.2|MmY_78994_33_4      CTCCTGCACATAGGGGT---ACTGCATATGTCTCCCCTTACCGAAAGTAT 3087
ref|NT_099035.1|MmY_98672_33_c      CTCCTGCACATAGGGGT---ACTTCATATGTCTCCCCTTACCCAAAGTAT 3079
ref|NT_099026.1|MmY_98663_33_c      ACCATTGATTCATCTCTCTAATCCCATAAAGATATCTTTTTAAGAAATC 3073
                                      * *  *   *    *   *   ****    *  * **    * *

ref|NT_099035.1|MmY_98672_33_5      TCAACTTACTGT-CTTTTACTGAATTGGCTTTTAGACTATAC--CTGAAA 3126
ref|NT_078929.2|MmY_78994_33_4      TCAACTTACTGT-CTTTTACTGAATTGGCTTTTAGACTATAC--CTGAAA 3134
ref|NT_099035.1|MmY_98672_33_c      TCCACTTACTGTTCTTTTACTGAATTGGCTTTTAGACTATAC--CTGAAA 3127
ref|NT_099026.1|MmY_98663_33_c      CAAAATTCACATGCATAAAACAGACCAGATTGAAGATAAGAAAGTTCTAA 3123
                                       * **    * * *  *    *   * **  ***  * *    *  **

ref|NT_099035.1|MmY_98672_33_5      TATTCCACTTTTCACCAAAGGATCTACCAGCCCTCCCTGTTCTAATAGAG 3176
ref|NT_078929.2|MmY_78994_33_4      TATTCCACTTTTCACCAAAGGATCTACCAGCCCTCCCTGTTCTAATAGAG 3184
ref|NT_099035.1|MmY_98672_33_c      TATTCCACTTTTCACCAAAGGATCTTCCAGCCCTCCCTGTTCTAATAGAG 3177
ref|NT_099026.1|MmY_98663_33_c      T-TGATGGTGACCATTAAAAGCAATAAGGGACACACAAAGAGTAGAGGAC 3172
                                    * *     *   **  *** *   *    * *   *      **   **

ref|NT_099035.1|MmY_98672_33_5      AGCTACAGTCCACCACTATCCAA---AGGGTTCTCAGACATT----CTAA 3219
ref|NT_078929.2|MmY_78994_33_4      AGCTACAGTCCACCAATATCCAA---AGGGTTCTCAGACATT----CTAA 3227
ref|NT_099035.1|MmY_98672_33_c      AGCTACCGTCCACCAATATCCAA---AGGGTTCTCAGACATT----CTAA 3220
ref|NT_099026.1|MmY_98663_33_c      ACTGAGAGACAAATGAATTCTAAGTTAGAGTTCTCAAAAAGTTAAGCTGG 3222
                                    *   *  * * *      ** **   ** ******* * * *    **

ref|NT_099035.1|MmY_98672_33_5      ATATGCCCCTGTGGGAGTAAACTTACTAAGTCTATTTAA-TTGGGCC--- 3265
ref|NT_078929.2|MmY_78994_33_4      ATATGCCCCTGTGGGAGTAAACTTACTAAGTCTATTTAA-TTGGGCC--- 3273
ref|NT_099035.1|MmY_98672_33_c      ATATGCCCCTGTGGGAGTAAACTTACTAAGTCTATTTAA-TTGGGCC--- 3266
ref|NT_099026.1|MmY_98663_33_c      AGACACAGGAAGAAGAGAAATTTTAAGGAAATAGTCAAACTTGGGCCATA 3272
                                    * *  *        *** **  ***   *     *  ** *******

ref|NT_099035.1|MmY_98672_33_5      -TCAGATC--CTTCTGCTTTGACCAAAAGGATTTCAATCTGCTGACTCTG 3312
ref|NT_078929.2|MmY_78994_33_4      -TCAGATC--CTTCTGCTTTGACCAAAAGGATTTCAATCTACTGACTCTG 3320
ref|NT_099035.1|MmY_98672_33_c      -TCAGATC--CTTCTGCTTTGACCAAAAAGATTTCAATCTACTGACTCTG 3313
ref|NT_099026.1|MmY_98663_33_c      TCCACATTGGCTTCTTTGTTTTCTTAACAAATAGCCAGCCTGTCATTACA 3322
                                      ** **   *****   **  *  **   **  * * *   * * *
```

```
ref|NT_099035.1|MmY_98672_33_5      AGTTACCAGGGGCCTCAGATCTCATTT-TCCCCAAGTGGTCAT-CAGATG 3360
ref|NT_078929.2|MmY_78994_33_4      AGTTACCAGGGGCCTCAGATCTCATTT-TCCCCAAGTGGTCAT-CAGATG 3368
ref|NT_099035.1|MmY_98672_33_c      AGTTACCAGGGGCCTCAGATCTCATTT-TCCCCAAGTGGTCAT-CAAATA 3361
ref|NT_099026.1|MmY_98663_33_c      GATGGGTGTGTGTGTGTGTGTGTGTGTGTGTGTGTGTGTGTGTGTGTGTG 3372
                                      *      * *  *  *      * * *      ***    *     *

ref|NT_099035.1|MmY_98672_33_5      TCCCTTTCTG-ACCAATAATGACCTTTCAACGTTCTCCTCTGATGCAGAC 3409
ref|NT_078929.2|MmY_78994_33_4      TCCCTTTCTG-ACCAATAATGACCTTGCAATGTTCTCCTCTGATGCAGAC 3417
ref|NT_099035.1|MmY_98672_33_c      TGCCTTTCTG-ACCAATAATGACCTTGCAATGTTCTCCTCTGATGGAGAC 3410
ref|NT_099026.1|MmY_98663_33_c      TTTTTGTGTGTGTTGTTTGTGAGTGTGTGTGTGTGTGTGTGTGTGTGTCC 3422
                                    *   * * **      *  ***   *       * *       **    *

ref|NT_099035.1|MmY_98672_33_5      ACTTAGACACCCCCCCTCCAATTTTAACTCCCAAATGGACCTCCAGACCA 3459
ref|NT_078929.2|MmY_78994_33_4      ACTTAGACACCCCCC-TCCAATTTTAACTCCCAAATGGACCTCCAGACCA 3466
ref|NT_099035.1|MmY_98672_33_c      ACTTAGTCACCCCCC-TCTAATTTTAACTCCCAAATGGACCTCCAGACCA 3459
ref|NT_099026.1|MmY_98663_33_c      ATTTTTACACAGGCCATCTAGACT-AGTGAATGTTTATATTTATGAGACT 3471
                                    * **   ***   ** ** *   * *         *  *  *      *

ref|NT_099035.1|MmY_98672_33_5      CCCAAGCTTAAATTACCTGAGCCG-AACATTAAGAAGTACAGCCCAGAAC 3508
ref|NT_078929.2|MmY_78994_33_4      CCCAAGCTTAAAGTACCTGAGCCG-AACATTAAGAAGTACAGCCCAGAAC 3515
ref|NT_099035.1|MmY_98672_33_c      CCCAAGCTTAAATTACCTGAACCG-AACATTAAGAAGTACAGCCCAGAAC 3508
ref|NT_099026.1|MmY_98663_33_c      CCCTAAATCA--TCACAGAAACCTTGATTTTGTGTAATGAGTTCCAAAAG 3519
                                    *** *  * *    **   * **   *  **  * * *     *** **

ref|NT_099035.1|MmY_98672_33_5      AAAAGCTCATTTCCTCAAGGCAGAAACTAAAATGATGTTCTCCTCACCAT 3558
ref|NT_078929.2|MmY_78994_33_4      AAAAGCTCATTTCCTCAAGGCAGAAACTAAAATGATGTTCTCCTCACCAT 3565
ref|NT_099035.1|MmY_98672_33_c      AAAAGCTCATTTCCTCAATGCAGAAACTAAAATGATGTACTCCTCACCAT 3558
ref|NT_099026.1|MmY_98663_33_c      TAAAG-TAAAACAGAAAATGAATATGCTGTGAGATAATTTTGGGTTGGAA 3568
                                     **** * *       ** * * *  **   *     *  *       *

ref|NT_099035.1|MmY_98672_33_5      TCATACCTAGACCCAGGAGATGAACATATGCCTGAGAAGAAGAACTAGTG 3608
ref|NT_078929.2|MmY_78994_33_4      TCATACCTAGACCCAGGAGATGAACATATGCCTGAGAAGAAGAACTAGTG 3615
ref|NT_099035.1|MmY_98672_33_c      TCATACCTAGACCCAGGAGAAGAACATATGCCTGAGAAGAAGAACTAGTG 3608
ref|NT_099026.1|MmY_98663_33_c      TGGATCCTGAGTTCCTTCCGTGAGCACTCTTTCCCTGTGATGAATCC-TG 3617
                                    *    ***     *       ** **            ** ***    **

ref|NT_099035.1|MmY_98672_33_5      TGGAACTGTCAGCCAGGTTTTTGATGAG-TGCTCAGACTGCTTCCTGATC 3657
ref|NT_078929.2|MmY_78994_33_4      TGGAACTGTCAGCCAGGTTTTTGATGAG-TGCTCAGACTGCTTCCTGATC 3664
ref|NT_099035.1|MmY_98672_33_c      TGGAACTGTCAGCCAGGTTTTTGGTGAG-TGCTCAGACTGCTTCCTGATC 3657
ref|NT_099026.1|MmY_98663_33_c      TG-ATCTTTTG--CAGGTCTCCAATGAGATCCCCAGATAACCATAAAA-- 3662
                                    ** * ** *    ***** *    **** * * ****   *      *

ref|NT_099035.1|MmY_98672_33_5      CAGCACCACATTTACTATAGTGGACAGATGCTCTAGTCTCTGATGAGGAG 3707
ref|NT_078929.2|MmY_78994_33_4      CGGCACCACATTTACTATAGTGGACAGATGCTCTAGTCTCTGATGAGGAG 3714
ref|NT_099035.1|MmY_98672_33_c      TGGCACCACATTTACTACAGTGGACAGATGCTCTAGTCTCTGATAAGGAG 3707
ref|NT_099026.1|MmY_98663_33_c      -AGCCCCACAGTGGCC-TGTTCCACCTA-GCTCTCCTCTAAACACATGAC 3709
                                      ** ***** *  *     *  **  * *****  ***      * **

ref|NT_099035.1|MmY_98672_33_5      TTGCTAGCTG-TTAGGAAGAACCTGGAATGGGCTCCA--CTGCATGAGGT 3754
ref|NT_078929.2|MmY_78994_33_4      TTGCTAGCTG-TTAGGAAAAACCTGGAATGGGCTCCA--CTGCATGAGGT 3761
ref|NT_099035.1|MmY_98672_33_c      TTGCTAGCTG-TTAGGAAGTACCTGTAATGGGCTCCA--CTGCATGAGGT 3754
ref|NT_099026.1|MmY_98663_33_c      CCCCTACATAACTATGTATAACTTCAGCCTGTCTATAGCCTGGAAATTCT 3759
                                       ***  *   ** * *  ** *      * **  *  *** *     *

ref|NT_099035.1|MmY_98672_33_5      AGGCTGTGTCTTTGATCTCACGTCAAGGAAAGAACATGCAAATTTGAACT 3804
ref|NT_078929.2|MmY_78994_33_4      AGGCTGTGTCTTTGATCTCACGTCAAGGAAAGAACATGCAAATTTGAACT 3811
ref|NT_099035.1|MmY_98672_33_c      AGGCTGAGTCTTTGAGCTCACGTCAAGGAAAGAACATGCAAATTTAAACT 3804
ref|NT_099026.1|MmY_98663_33_c      AGTCTTTTACTTATATGTGACCTCATTCTTAGAAAAC----ACTGAAGGT 3805
                                    ** **    ***  *  * ** ***     **** *     * *  *  *
```

```
ref|NT_099035.1|MmY_98672_33_5      CTACCCAAC-CAAACATACTAGTTTACCTG-TACAA-AGGGGATTTAGAT 3851
ref|NT_078929.2|MmY_78994_33_4      CTACCCAAC-CAAACATACTAGTTTACCTG-TACAA-AGGGGATTTAGAT 3858
ref|NT_099035.1|MmY_98672_33_c      CTACCCAAC-CAAACATACTAGTTTACCTG-TACAA-AGGGGTTTTAGAT 3851
ref|NT_099026.1|MmY_98663_33_c      GTAACCAATGCATATATGCTGACCTCTTTTCTACGCCAGTTGACATGGGT 3855
                                     ** ****  ** * ** **    *   *  ***   **  *   * * *

ref|NT_099035.1|MmY_98672_33_5      TTCCAATTTCTCACAAAGATACCC-------------CTCTTACCATTGA 3888
ref|NT_078929.2|MmY_78994_33_4      TTCCAATTTCTCACAAAGATACCC-------------CTCTTACCATTGA 3895
ref|NT_099035.1|MmY_98672_33_c      TTCCAAATTCTCACAAAGATGCCCTCGTGTATTTCCCCTCTGACCATTGG 3901
ref|NT_099026.1|MmY_98663_33_c      TTCAAA--TGTTATGAGTTTCCCCACTGT--GTGGGTCACTTTTTGAGGA 3901
                                    *** **  * * *  *   * ***             * **       *

ref|NT_099035.1|MmY_98672_33_5      GTTCCTATCTATTTTTCATGGCTCATAGGGTCTCATTTCTTCCCAATGTC 3938
ref|NT_078929.2|MmY_78994_33_4      GTTCCTATCTATTTTTCATGGCTCATAGGGTCTCATTTCTTCCCAATGTC 3945
ref|NT_099035.1|MmY_98672_33_c      GGTCCTATCTATTTTTCATGGCTCATAGGGTCTCATTTCTTCCCAATGTC 3951
ref|NT_099026.1|MmY_98663_33_c      GCCCGTGTACTTCATGGAGGTCTGCCTGGGTTCCATCTATACTCTCAGAG 3951
                                    *  * * *   *  *  * * **    ****  *** * * * *   *

ref|NT_099035.1|MmY_98672_33_5      CATTACTGGCTCACTTCTGACCACTAGCCATACATCTCTGATTAGTGTTG 3988
ref|NT_078929.2|MmY_78994_33_4      CATTACTGGCTCACTTCTGACCACTAGCCATACATCTCTGATTAGTGTTG 3995
ref|NT_099035.1|MmY_98672_33_c      CATTACCCACTCACCTCTGACCACTAGCCATACATCTCTGATTAGTGTTG 4001
ref|NT_099026.1|MmY_98663_33_c      -GTTTTTTTTTTTTTTTTTTTTTTTTTTTTTTTTTTTTTTTTTTTGAGGCA 4000
                                      **      *    * *      *     *   * * *  ** * *

ref|NT_099035.1|MmY_98672_33_5      GGGGTGTTCTCGGTATGA-CACTAAGGCCAACAATAGT-GCCTACATGCC 4036
ref|NT_078929.2|MmY_78994_33_4      GGGGTGTTCTCGGTATGA-CACTAAGGCCAACAATAGT-GCCTACATGCC 4043
ref|NT_099035.1|MmY_98672_33_c      GGGATGTTCTCGGTATGA-CAATAAGGCCAACAGTAGT-GCCTACATGCC 4049
ref|NT_099026.1|MmY_98663_33_c      GGAATGCCATGGACTCAGTCATTACAATTTTCCTCTCTAGAATAAGTTTG 4050
                                    **  **   * *       ** **       *     *  **   *

ref|NT_099035.1|MmY_98672_33_5      ATTTCTGAATAATTGGATCTTAAATTCCACCATTGTCCAAGTGTACT-TC 4085
ref|NT_078929.2|MmY_78994_33_4      ATTTCTGAATAATTGGATCTTAAATTCCACCATTGTCCAAGTGTACT-TC 4092
ref|NT_099035.1|MmY_98672_33_c      ATTTTTGAAAAATTGGATCTTAAATTCCACCTCTGTCCAAGGGTACT-TC 4098
ref|NT_099026.1|MmY_98663_33_c      TATGCTTCAGCATTTGATAATAATCTCTATTGTTGATTAATTTTAATATC 4100
                                      *  *  *  *** ***  ***  ** *    **   **   ** * **

ref|NT_099035.1|MmY_98672_33_5      TGAAAGACTTTTCTCATTACATGGGGTCTCTACAGTAGCATCTTTGTTCA 4135
ref|NT_078929.2|MmY_78994_33_4      TGAAAGACTTTTCTCATTACATGGGGTCTCTACAGTAGCATCTTTGTTCA 4142
ref|NT_099035.1|MmY_98672_33_c      AGAAAGACCTTTCTCATTACATGGGTTCTCTATAGTAGCTTCTTTATTCA 4148
ref|NT_099026.1|MmY_98663_33_c      TGGGAGAGTTTCTGCAGTA-ACTGATTCTCTTAGGGATTAGTTGCTGTGA 4149
                                     *  ***  **   ** ** *  *  *****   * *     *    * *

ref|NT_099035.1|MmY_98672_33_5      TCAGGCCTCTAGTTGTACCTTCTGGCCTAAGCAGATCTTATACTTCACTT 4185
ref|NT_078929.2|MmY_78994_33_4      TCAGGCCTCTAGTTGTACCTTCTGGCCTAAGCAGGTTTTATACTTCAATT 4192
ref|NT_099035.1|MmY_98672_33_c      ACAGGCCTCTGGTTGTACCTTCTGGCC-AAGCAGGTCTTACACTTCACTT 4197
ref|NT_099026.1|MmY_98663_33_c      AAAGACACCATGTCAAAGGCCA----CTCATAAGAGCCAACATTTCA-TT 4194
                                      ** *  *  **   *         *  *  **     * * **** **

ref|NT_099035.1|MmY_98672_33_5      AAAGAGATGCCAAATTTACACAATGTAGACAAAGATGCATTGAGCATTC- 4234
ref|NT_078929.2|MmY_78994_33_4      AAAGAGATGCCAAATTTACACAATGTAGACAAAGATGCATTGAGCATTC- 4241
ref|NT_099035.1|MmY_98672_33_c      AAAGAGATGCCACATTTACACAATGTAGACAAAAAGGATTTGAGCATTC- 4246
ref|NT_099026.1|MmY_98663_33_c      AGTGCTTTTCTACATTT-CATTACACGTTCAGAGATTTACTCCACGATCA 4243
                                    *  *   * * * **** **  *      ** * *     *   *  **

ref|NT_099035.1|MmY_98672_33_5      TCATACTCATTAATATAACATTCCTCCCCTCAGGTTTGAAGTCTCCAGAG 4284
ref|NT_078929.2|MmY_78994_33_4      TCATACTCATTAATATAACATTCCTCCCCTCAGGTTTGAAGTCTCCAGAG 4291
ref|NT_099035.1|MmY_98672_33_c      TCATACTCATCAATATAACATTCCTCCCCTCAGGTTTGAAGTCTCCAGAG 4296
ref|NT_099026.1|MmY_98663_33_c      TCAAGGCAGAAAATATGGCACT-----------GTCT-AGGCAGACATGG 4281
                                    ***        *****  ** *           ** * * *    **  *
```

```
ref|NT_099035.1|MmY_98672_33_5      CAGCAAAGAGAGTGGGGAGTAGAACTCATCATCTTTAGGTCACTCAGCAT 4334
ref|NT_078929.2|MmY_78994_33_4      CAGCAAAGAGAGTGGGGAGTAGAACTCATCATCTTTAGGTCACTCAACAT 4341
ref|NT_099035.1|MmY_98672_33_c      CAGCAAGGAGAGTGGGGAGTAGAACTCATCATCTTTATGTCACTCATCAC 4346
ref|NT_099026.1|MmY_98663_33_c      TGGTGGAGAG---GAAGTTTAGAATTATGCATCTTCATCCAAAT--GCTT 4326
                                      *    ***   *  *  ***** *   ****** *    * *   *

ref|NT_099035.1|MmY_98672_33_5      CGATGGGTTGTTTGCAACCTTAGTATACAGACATGGATGGTTGAACAC-- 4382
ref|NT_078929.2|MmY_78994_33_4      CGATGGGTTGTTTGCAACCTTAGTATACAGACATGGATGGTTGAACAC-- 4389
ref|NT_099035.1|MmY_98672_33_c      CGATGGTTTGTTCCCAACCTTAGTATACAGACATGGATGGTTGAACAC-- 4394
ref|NT_099026.1|MmY_98663_33_c      CCA-GGAAAAACTATGTATTCTGCATGCAGGGAGGGTCTCTTCCACACTG 4375
                                    * * **             *  * ** ***  * **    **  ****

ref|NT_099035.1|MmY_98672_33_5      --AAACCTGTCAGGCTGATAAAGAACTAGACATCTACTAACGTCCTTGGA 4430
ref|NT_078929.2|MmY_78994_33_4      --AAACCTGTCAGGCTGATAAAGAACTAGACATCTACTAACGTCCTTGGA 4437
ref|NT_099035.1|MmY_98672_33_c      --AAACCTGTCAGGCTGACAACGCAATAGACATCTACTAAAGTCCTTGGA 4442
ref|NT_099026.1|MmY_98663_33_c      GTAGAGCTTTAAAGCCC-CAAAATGATATGCTTCCTCCAGCAAGGCCAGA 4424
                                      * * ** * * **    **     **  * **  * *         **

ref|NT_099035.1|MmY_98672_33_5      ACCATGTTCAGATTGCTGCACATCCTCCTGAAGATGCCACAGATGACATG 4480
ref|NT_078929.2|MmY_78994_33_4      ACCATTTTCAGATTGCTGCACATCCTCCTGAAGATGCCACAGATGACATG 4487
ref|NT_099035.1|MmY_98672_33_c      ACCATGTTCAGAGTGCTGCACATCCTCCTGAAGATGCCACGGATGACATG 4492
ref|NT_099026.1|MmY_98663_33_c      CCTATTCCTACAAGGCCACACCTCCTCATAGACAAAATATATTTAGAATG 4474
                                     * **    * *  **  *** ***** *  * *    *    *   ***

ref|NT_099035.1|MmY_98672_33_5      GTTCCTTGTGATTGTTGTTCTCTTCTTGTTGTTGCTGTTTTCTTTT---- 4526
ref|NT_078929.2|MmY_78994_33_4      GTTCCTTGTGATTGTTGTTCTCTTCTTGTTGTTGCTGTTTTCTTTT---- 4533
ref|NT_099035.1|MmY_98672_33_c      GTTCCTAGTGATTGTTGTTCTCTTCTTGTTGTTGCTGTTTTCTTTT---- 4538
ref|NT_099026.1|MmY_98663_33_c      CCACACTGGTTTCATAGCAATTGACAAGATCTAGCTTCTTCAAAGAGGAG 4524
                                       *   *   *  * *   *   *  * * * ***  **

ref|NT_099035.1|MmY_98672_33_5      -AACTTT--TGAAGAGGATACTTTATTAGTCTGTTGCTATGTTAGTCATC 4573
ref|NT_078929.2|MmY_78994_33_4      -AACTTT--TGAAGAGGATACTTTATTAGTCTGTTGCTATGTTAGTCATC 4580
ref|NT_099035.1|MmY_98672_33_c      -AACTTT--TGAAGAGGATACTTTATTAGTCTGTTGCTATGTTAGTCATC 4585
ref|NT_099026.1|MmY_98663_33_c      GAGCCTCAGTGGAGAAGATGCCTCCACAAGATCCAGCTGT-TAGGAAATT 4573
                                     * * *   ** *** *** * *    *   *   *** * *  *  **

ref|NT_099035.1|MmY_98672_33_5      TCCTGGCATTAGAAACCAACATCCCACTGTTTAAATGTTTTACATTTCCT 4623
ref|NT_078929.2|MmY_78994_33_4      TCCTGGCATTAGAAACCAACATCCCACTGTTTAAATGTTTTACATTTCCT 4630
ref|NT_099035.1|MmY_98672_33_c      TCCTGGCATTAGAAACCAACATGCCACTGCTTAAATGTTTTACATTTCCT 4635
ref|NT_099026.1|MmY_98663_33_c      TCATA--ATTGGTGGGAGAGCACCCACTCCATTGTGGGTGTTGACATCCT 4621
                                    ** *   *** *      *    *****   *    * * *  *  ****

ref|NT_099035.1|MmY_98672_33_5      ACTCTTTTTGGAAAACACAACGTATGCGCATCATTTTTTCCTATTGTTTC 4673
ref|NT_078929.2|MmY_78994_33_4      ACTCTTTTTGGAAAACACAACGTATGCACATCATTTTTTCCTATTGTTTC 4680
ref|NT_099035.1|MmY_98672_33_c      ACTCTTTTTGGAAAACACAATGTATGCACATCATTTTTTCCTATTTTTTC 4685
ref|NT_099026.1|MmY_98663_33_c      TGTATTAGTAGTCCTGGGTTCTTTAAGAAAGCAATCTAAGCAAG-ACTAG 4670
                                      * **  * *           *      * ** * *   * *    *

ref|NT_099035.1|MmY_98672_33_5      AAAGACATACCACTATGTAATTCAACTTTACAATT-----GTAAACATTT 4718
ref|NT_078929.2|MmY_78994_33_4      AAAGACATACCACTATGTAATTCAACTTTACAATT-----GTAAACATTT 4725
ref|NT_099035.1|MmY_98672_33_c      AAAGACATACCACTATGTAATTCAACTTTACAATT-----GTAATCATTT 4730
ref|NT_099026.1|MmY_98663_33_c      AGGAGCAAGACAATAAGCATCACCACACCATAGCCTCTGAATAAGCTCCT 4720
                                    *    **  ** ** * *   * **   * *         *** *   *

ref|NT_099035.1|MmY_98672_33_5      TGACCAATATTCACATGAATATAAATTTTATGTGTAAAATTCTTCCTGTG 4768
ref|NT_078929.2|MmY_78994_33_4      TGACCAATATTCACATGAATATAAATTTTATGTGTAAACTTCCTCCTGTG 4775
ref|NT_099035.1|MmY_98672_33_c      TGACCAATATTCACATGAATATAAATTTTATGTGTAAACATCTTCCTGTG 4780
ref|NT_099026.1|MmY_98663_33_c      GGCTCCAGGATCCTTTGTTGTTTCAGTTTCTGTCCCAACTTCTTTGATAA 4770
                                     *  * *   **   **    *  * *** ***   **  ** *
```

```
ref|NT_099035.1|MmY_98672_33_5      CTGT--GTTATTTTTCACCAGATGACAGTTTCATTTGAGGTCATTTGAAA 4816
ref|NT_078929.2|MmY_78994_33_4      CTGT--GTTATTTTTCACCAGATGACAGTTTCATTTGAGGTCATTTGAAA 4823
ref|NT_099035.1|MmY_98672_33_c      CTGT--GTTATTTTCCACCAGTTGACAGTTTCATTTGAGGTAATTTGGAA 4828
ref|NT_099026.1|MmY_98663_33_c      TTAACAACAATGTGGAAGTATAAGCTGAATACAACCTTTGCTATGGAATT 4820
                                     *       ** *   *  *   *     * **      *  **

ref|NT_099035.1|MmY_98672_33_5      CA-TCGTTCCTTACTATGTGTCTTTAAAA-GATATATTTTGGAGCTGAGG 4864
ref|NT_078929.2|MmY_78994_33_4      GA-TCATTCCTTACTATGTGTCTTTAAAA-GATATATTTTGGAGCTGAGG 4871
ref|NT_099035.1|MmY_98672_33_c      GT-TCGTTCCTTACTATGTGTCTTTAAAA-GATATATTTTGGAGCTGAGG 4876
ref|NT_099026.1|MmY_98663_33_c      TGCTCTTTGGACATGGTGGATCCTTACAACAATAAACATAGTAAGACATT 4870
                                       ** **    *   **  ** *** **  *** *  * * *    *

ref|NT_099035.1|MmY_98672_33_5      GGTATGGCTGAATATCTT-CAGCACAATAAACACCTTCTATAACATTACT 4913
ref|NT_078929.2|MmY_78994_33_4      GGTATGGCTGAATATCTT-CAGCACAATAAACACCTTCTATAACATTACT 4920
ref|NT_099035.1|MmY_98672_33_c      GGTATGGCTGAATATCTT-CAGCACAATAAATACCTTCTGTAACGTTACT 4925
ref|NT_099026.1|MmY_98663_33_c      CATA-GGAAAAATGTCTAACAGTGCCAATGCCACTAATCTTAAAACCAC- 4918
                                      ** **   *** ***  ***  * *     **      ***    **

ref|NT_099035.1|MmY_98672_33_5      ATCTAGTGGATTATAAGTATCAGGTTGT-ACACAGTAGTAAC-TAAAATA 4961
ref|NT_078929.2|MmY_78994_33_4      ATCTAGTGGATTATAAGTATCAGGTTGT-ACACAGTAGTAAC-TAAAATA 4968
ref|NT_099035.1|MmY_98672_33_c      ATCTAGTGTATTATAAGTATCAGGTTGT-ACACAGTAGTAAG-TAAAATA 4973
ref|NT_099026.1|MmY_98663_33_c      AAATAATAATGTGTAGACATTTTATTTTTATTTAAAGGTAAGATATACTC 4968
                                    *  ** *    * **   **    ** * *   *   ****  ** * *

ref|NT_099035.1|MmY_98672_33_5      ATGGACAA-TTTGAGA---AAGATTTTAGGGTGAAATCTCTCATAATATA 5007
ref|NT_078929.2|MmY_78994_33_4      ATGGACAA-TTTGAGA---AAGATTTTAGGGTGAAATCTCTCATAATATA 5014
ref|NT_099035.1|MmY_98672_33_c      ATGGACAAGTTTGAGA---AAGATTTTAGGGTGTAATCTCTCATAATATA 5020
ref|NT_099026.1|MmY_98663_33_c      GTGTACCTACCTGAGATGTAATTGTTCTAGTTTGCTTCTTTCTCACTATG 5018
                                     ** **     *****   **   **   * *    *** **  * ***

ref|NT_099035.1|MmY_98672_33_5      CAGAAAA----AAAAGGAAAT--CCTAGATATTAA-----TCAAATCTAT 5046
ref|NT_078929.2|MmY_78994_33_4      CAGAAAA----AAA-GGAAAT--CCTAGATATTAA-----TCAAATCTAT 5052
ref|NT_099035.1|MmY_98672_33_c      CACAAAA----AGA---AAAT--CCTAGATATTAA-----TCAAATCTAT 5056
ref|NT_099026.1|MmY_98663_33_c      TAGAATACTACAATCAAAAATAACTTGGAAATAGAAGTGTTATAGTCTAT 5068
                                     * ** *    *     ****  * * ** **  *     *  * *****

ref|NT_099035.1|MmY_98672_33_5      -GCACACCAAATGAACATTGTGGGTTTTGTGGGTT-TGTTTGTATATGTC 5094
ref|NT_078929.2|MmY_78994_33_4      -GCACACCAAATGAACATTGTGGGTTTTGTGGGTT-TGTTTGTATATGTC 5100
ref|NT_099035.1|MmY_98672_33_c      -GCACACCAAATGAACATTATGAGTTTTGTGGATT-TGTTTTTATATGTC 5104
ref|NT_099026.1|MmY_98663_33_c      CACCGAGGAAGACAAAACAGGAAGTTGTATCAGTAACAGCAAAAAACAGC 5118
                                      *  *  **   ** *      *** * *   *         * *   *

ref|NT_099035.1|MmY_98672_33_5      TTTGTAGTTCGAATGTCCAACCTTTATGTCTTAATTGATGATTGTAAACA 5144
ref|NT_078929.2|MmY_78994_33_4      TTTGTAGTTCGAATGTCCAACCTTTATGTCTTAATTGATGATTGTAAACA 5150
ref|NT_099035.1|MmY_98672_33_c      TTTGTAGTTCGAATGTCCAACCTTTATGTCTTAATTGAGGATTGTAAACA 5154
ref|NT_099026.1|MmY_98663_33_c      TTATTGGCTTGC---CACTGTCTTAGTCACTCTTTTATTGAT-GTGAAGA 5164
                                    **  * * * *      *   ***  *  **   **   *** ** ** *

ref|NT_099035.1|MmY_98672_33_5      GTGCTCAGAGTGTGTGCATGTGAATTAAAGGGCACCATGTCTTTAGTGTT 5194
ref|NT_078929.2|MmY_78994_33_4      GTGCTCAGAGTGTGTGCATGTGAATTAAAGGGCACCATGTCTTTAGTGTT 5200
ref|NT_099035.1|MmY_98672_33_c      ATGCTCAGGGTGTATGTGTGTGAGTTAAAGGGCACCATGTCTTTAGTGTT 5204
ref|NT_099026.1|MmY_98663_33_c      GATACCATGGATAAGGCAACTATTAGAAAACAAAACATTTAACTAGAGGC 5214
                                         **  *     *    *     ***    * *** *   *** *

ref|NT_099035.1|MmY_98672_33_5      TAGATTAA--TTTCACATATCTGTCTATCTGTC-ATCTATG-----TATG 5236
ref|NT_078929.2|MmY_78994_33_4      TACATTAA--TTTCACATAT--AATTATCTGTCTATCTGT------CATC 5240
ref|NT_099035.1|MmY_98672_33_c      TAGATTAA--TATCACATATC--ATTATCTGTCTATCCATCA--TCTATA 5248
ref|NT_099026.1|MmY_98663_33_c      TTGCTTACATTTTCAGAAGTTTGGTCATTCACCAAGGGAGGGATCCCATC 5264
                                    *   ***   * *** *  *      **    * *            **
```

```
ref|NT_099035.1|MmY_98672_33_5      TATGTATGTATGTATGTATGTATGTA-TGAAGTAATGATTGAA----TGT 5281
ref|NT_078929.2|MmY_78994_33_4      TATGTATGTATGTATGTATGTATGTA-TGAAGTAATGATTGAA----TGT 5285
ref|NT_099035.1|MmY_98672_33_c      TATGTATGTATGTATGTTTGTATGTA-TGAAGTAATGATTGAA----TGT 5293
ref|NT_099026.1|MmY_98663_33_c      ACTGGAGCAATAGAGAACTATTGATCCTTAAGCAAAGAGAGAGATTCTGC 5314
                                      ** *   **  *    * *   *  * *** ** **  **     **

ref|NT_099035.1|MmY_98672_33_5      ATTTATATAGATATGTATGCAACT--ATTTCTCTATCATCTACCTATC-- 5327
ref|NT_078929.2|MmY_78994_33_4      ATTTATATAGATATGTATGCAACT--ATTTCTCTATCATCTACCTATC-- 5331
ref|NT_099035.1|MmY_98672_33_c      ATTTATATAGATATGTATGCAACT--ATTTCTCTATCATCTACCAATCAC 5341
ref|NT_099026.1|MmY_98663_33_c      CATTGCTTTGGCTTCTTGAAAACTCAAACCCATCCTTGTTGACACATC-C 5363
                                      **   * *   * *    ****  *   *    *  * **   ***

ref|NT_099035.1|MmY_98672_33_5      TCTCTCTCTTTCTATTTCTCTCACCCTCTTTCTCTGTTTATCTCTTATGC 5377
ref|NT_078929.2|MmY_78994_33_4      TCTCTCTCTTTCTATTTCTCTCACCCTCTTTCTCTGTTTATCTCTTATGC 5381
ref|NT_099035.1|MmY_98672_33_c      TCTTTCTCTTTCTATTTCTCTCACCCTCTCTCTCTGTTTATCTCTTATGC 5391
ref|NT_099026.1|MmY_98663_33_c      ACTTCCTCCAATGAAGCCACACATTCTCTTATTCCTTCTAATCTTTAAAC 5413
                                     **  ***     *   * * **  ****   **  * **    ***  *

ref|NT_099035.1|MmY_98672_33_5      --ATGAATATATGCATGCATGTAGCTAGGTAGGTGTG-GTATTTTTAATG 5424
ref|NT_078929.2|MmY_78994_33_4      --ATGAATATATGCATGCATGTAGCTAGGTAGGTGTG-GTATTTTTAATG 5428
ref|NT_099035.1|MmY_98672_33_c      --ATGAATATATGTATGCATGTAGCTAGGTAGGTGTG-GTAGTTTTAATG 5438
ref|NT_099026.1|MmY_98663_33_c      TCACTGCCACTTCCCAGTGACTAAGTATTCATATATATGTGTCTATAGAG 5463
                                      *     *  *    *    **  **   *  * *  **   * **  *

ref|NT_099035.1|MmY_98672_33_5      TAATTATTT--CCCATCGGCCCATAGGCTGTATTAGGATGAT------TA 5466
ref|NT_078929.2|MmY_78994_33_4      TAATTGGTT--CCCATCGGCCCATAGGCTGTATTAGGATGAT------TA 5470
ref|NT_099035.1|MmY_98672_33_c      TAATTGGTT--CCCATTGGCCCATAGGCTGTATTAGGGTGAG------TA 5480
ref|NT_099026.1|MmY_98663_33_c      AACTTTCTTATTCAAACTACCAATTTTCTATGGCTTGATCATCTTTTATT 5513
                                     * **  **   * *    ** **   ** *     * * *       *

ref|NT_099035.1|MmY_98672_33_5      ACCTTGTTGGA--CTAGGTGTTTCCTTGTTGGAG-GAAGGGTGTCA--CT 5511
ref|NT_078929.2|MmY_78994_33_4      ACCTTGTTGGA--CTAGGTGTTTCCTTGTTGGAG-GAAGGGTGTCA--CT 5515
ref|NT_099035.1|MmY_98672_33_c      ACCTTGATGGA--CTAGGTGTTTCCTTGTTGGAG-GAAAGGTGTCA--CT 5525
ref|NT_099026.1|MmY_98663_33_c      ATTTTAGTACAGTCTAAAAGGCTCCCAATTAAAATGACTACTGTTGAGCT 5563
                                    *  **  *  *  ***   *  ***   **  *  **    ***    **

ref|NT_099035.1|MmY_98672_33_5      GGAGGAGGTGGGTGGTCTCACACATGTTCAAGTTAGTTATGCACAGTAGG 5561
ref|NT_078929.2|MmY_78994_33_4      GGAGGAGGTGGGTGGTCTCACACATGTTCAAGTTAGTTATGCCCAGTAGG 5565
ref|NT_099035.1|MmY_98672_33_c      GGAAGAGGTGGGTGGTCTCATTCATGTTCAAGTTA----TGCCCAGTAGG 5571
ref|NT_099026.1|MmY_98663_33_c      TAGCCACAGTGGTGGGCCCTCCCACATC--AATCA-TTACCCAAGAAAAT 5610
                                         *    ***** * *   **  *   * * *      *     *

ref|NT_099035.1|MmY_98672_33_5      GCTCTGTGTGTTTGCTGTCTCTCTGTCTTTCCAAGAGTAGCTGTGATAGA 5611
ref|NT_078929.2|MmY_78994_33_4      GCTCTGTGTGTTTGCTGTCTCTCTGTCTTTCCAAGAGTAGCTGTGATAGA 5615
ref|NT_099035.1|MmY_98672_33_c      GTTCTGTGTGTTTGTTGTCTGTTTGTCTTTCCAAGAGTAGCTGTGATAGT 5621
ref|NT_099026.1|MmY_98663_33_c      ATCCTATAGACTTACCTAATAACCAAGTGAATAGAGACAGATCT--TCAA 5658
                                       ** *    **      *       *    *     ** * *  *

ref|NT_099035.1|MmY_98672_33_5      GTGTTCAGTGATTCAAGGAGGGGATGTTGGTTAGATGGCATATAAAGCAG 5661
ref|NT_078929.2|MmY_78994_33_4      GTGTTCAGTGATTCAAGGAGGGGATGTTGGTTAGATGGCATATAAAGCAG 5665
ref|NT_099035.1|MmY_98672_33_c      GTGTTCAGTGATTCAAGGAGGGGATGTTGGTTAGATGGCATATAAAGCAG 5671
ref|NT_099026.1|MmY_98663_33_c      TTGCTCTCTCTTCTCAGGTAT--CCGTTGATTGTCAAGATTGGGAAAAAA 5706
                                     ** **  *  *   ***       **** **     *  *   **  *

ref|NT_099035.1|MmY_98672_33_5      GCAAGACACCTT-TACATACATGGTAGAAGAAGCA-AGCTTTAAGAAGCA 5709
ref|NT_078929.2|MmY_78994_33_4      GCAAGACACATT-TACATACATGTTAGAAGAAGCA-AGCTTTAAGAAGCA 5713
ref|NT_099035.1|MmY_98672_33_c      GCAAGACACATT-TACATACATGGTAGAAGAAGCA-AACTTTAAGAAGCA 5719
ref|NT_099026.1|MmY_98663_33_c      TAAAGTCCCATCACAGGAATATTTACTGACTCCACAACCTACCCAAACA 5756
                                      *** * * *   *   * **  **       ** * * *    ** **
```

```
ref|NT_099035.1|MmY_98672_33_5      CAAACAATCACACACACACACACACACACACACACACACACACA------ 5753
ref|NT_078929.2|MmY_78994_33_4      CAAACAATCACACACACACACACACACACACACACACACACACACACACA 5763
ref|NT_099035.1|MmY_98672_33_c      CAAACAATCACACACACACACACACACACACACACACACACACACACACA 5769
ref|NT_099026.1|MmY_98663_33_c      TTACTGAACA-GCATTTCCTCCTCTCTGCCCCTTCATAGACCCACTTCCA 5805
                                      *   * **  **    * *       * *   ** * ** **

ref|NT_099035.1|MmY_98672_33_5      ----------------TTTGTTGGACAGTGCAAAGCTTCAACCACTTTAA 5787
ref|NT_078929.2|MmY_78994_33_4      CACA------------TTTGTTGGACAGTGCAAAGCTTCAACCACTTTAA 5801
ref|NT_099035.1|MmY_98672_33_c      CACACACACACACACATTTGTTGGACAGTGCAAAGCTTCAACCACTTTCC 5819
ref|NT_099026.1|MmY_98663_33_c      TGCA------------TGGCATAAGTGAAGGATAACTTCTTTCA-TCAGC 5842
                                                    *    *       * * * ****   ** *

ref|NT_099035.1|MmY_98672_33_5      ATATATACTCA-CATTTGGTGGATGGAACAAAGCACCAACTTTATTTTTC 5836
ref|NT_078929.2|MmY_78994_33_4      ATATATACTCA-CATTTGGTGGATGGAACAAAGCACCAACTTTATTTTTC 5850
ref|NT_099035.1|MmY_98672_33_c      ATATATACTCA-CCTTTGGTGGATGGAACAAAGCACCAACTTTATTTTTC 5868
ref|NT_099026.1|MmY_98663_33_c      ATTGATACTCAATAATAGGTGAAATGATGTGAATATGTCCTATAGCCACA 5892
                                    **  *******    * **** *  **    *  *    ** **

ref|NT_099035.1|MmY_98672_33_5      TCACACTGCTTATAAATCTCTGCACAATCTTTCAATTAGTATAA---AAT 5883
ref|NT_078929.2|MmY_78994_33_4      TCACACTGCTTATATATCTCTGGACAATCTTTCAATTAGTATAA---AAT 5897
ref|NT_099035.1|MmY_98672_33_c      TCACACTGCTTATATATCACTGCGCAATCTTTCAATTAGTATAA---AAT 5915
ref|NT_099026.1|MmY_98663_33_c      CAACCCCCCACATTCTACTGTGTGTTACACTTACTGGGGTACAACCCAAT 5942
                                      ** *  *  **    *  **    *   **      *** **   ***

ref|NT_099035.1|MmY_98672_33_5      TTATACTGGTTACTTTCTATTTTCTTCTAGTGAATAATTATGAGT--ATG 5931
ref|NT_078929.2|MmY_78994_33_4      TTATACTGGTTACTTTCTATTTTCTTCTAGTGAATAATTATGAGT--ATG 5945
ref|NT_099035.1|MmY_98672_33_c      TTGTACTGGTTACTTTCTATTTTCTTCTAGTGAATAATTATGAGA--ATA 5963
ref|NT_099026.1|MmY_98663_33_c      CTGTCCAATTTTCTGCCAACTCAGCTATGAGAAAAAAAAATCTCCTCATC 5992
                                     * * *   ** **  * * *    * *    ** **  **      **

ref|NT_099035.1|MmY_98672_33_5      TATAAGGAAAAGAATCTTCTCACTATACTTTTATGATGAGCTATTTATAA 5981
ref|NT_078929.2|MmY_78994_33_4      TATAAGGAGAAGCATCTTCTCACTATACTTTTATGATGAGCTATTTTTAA 5995
ref|NT_099035.1|MmY_98672_33_c      CATAAGGAAAAGCATCTTCTCACTATACTTTTATGATGAGCTATTTATAA 6013
ref|NT_099026.1|MmY_98663_33_c      AATAGATCCAATGAATTTTACCTGATTTCTTTCTCCTGGGGCAACTCAGT 6042
                                     ***     **  *  **  *   **   *** *  ** *  *  *

ref|NT_099035.1|MmY_98672_33_5      ATTATGTTTATAAATATCCAATATTATTCCCAAGAACCAACACGCATTTT 6031
ref|NT_078929.2|MmY_78994_33_4      ATTATGTTTATAAATATCCAATATTATTCCCAAGAACCAACACGCATTTC 6045
ref|NT_099035.1|MmY_98672_33_c      ATTATGTTTATAAATATCCAATATTATCCCCAAGAACCAACACACATTTC 6063
ref|NT_099026.1|MmY_98663_33_c      GTTATCCCTTCATGCATATATTGGTGTCTT-----------ATTCATCTG 6081
                                     ****   *  *   **  * *  * *              *  *** *

ref|NT_099035.1|MmY_98672_33_5      TAACTAAAAAACTTGTTATAGATTAACAAGTTGTAAGCAAGTAGGGTAAT 6081
ref|NT_078929.2|MmY_78994_33_4      TAACTAAAAAACTTGTTATAGATTAACAAGTTGTAAGCAAGTAGGGTAAT 6095
ref|NT_099035.1|MmY_98672_33_c      TAACTAAAAAACTTGTTATAGATTAACAAGTTGTAAGCAAGTAAGGTATT 6113
ref|NT_099026.1|MmY_98663_33_c      TA-CTAAAACCACTACTATTAATAGATACATCACAGG-AGATATGACAAA 6129
                                    ** ******    *  ***  **  * *  *   * * *  ** *  *

ref|NT_099035.1|MmY_98672_33_5      TTTTATTTCAGCCAGTTTCTCTTACTGGTAGACTGAAATTTGCCGTTATA 6131
ref|NT_078929.2|MmY_78994_33_4      TTTTATTTCAGCCAGTTTCTCTTACTGGTAGACTGAAATTTGCCGTTATA 6145
ref|NT_099035.1|MmY_98672_33_c      TTTT---TCAGCCAGTTTCTCTTACTGGTAGACTGAAATTTGCTGTTAAA 6160
ref|NT_099026.1|MmY_98663_33_c      TACCAACTCCCATGAGCAGCTGCACAGTTGGCCAAGCCTTTTCAGTGCTT 6179
                                    *      **              ** * * * *     *** * **

ref|NT_099035.1|MmY_98672_33_5      CAGAAAGGATTAGTTTTTAT-CTATCTCAAAAATTAATCCTTCAAAAATC 6180
ref|NT_078929.2|MmY_78994_33_4      CAGAAAGGATTAGTTTTTAT-CTATCTCAAAAATTAATCCTTCAAAAATC 6194
ref|NT_099035.1|MmY_98672_33_c      CATAAAGGATTAGTTTTTAT-CTATCTCAAAAATTAATCCTTCAAAAATC 6209
ref|NT_099026.1|MmY_98663_33_c      TTTTGAAGTTTCCTTTGTATACTAAAAAATCAATTGATTTCTTTAAGTGT 6229
                                         * * **  *** *** ***    *  **** **   *  **
```

```
ref|NT_099035.1|MmY_98672_33_5      TTAAAAGAATCACAAAACTAAACTTTTATATAAAAACCTATCATCCCTTT 6230
ref|NT_078929.2|MmY_78994_33_4      TTAAAAGAATCACAAAACTAAACTTTTATATAAAAACCTATCATCCCTTT 6244
ref|NT_099035.1|MmY_98672_33_c      TTAAAAGAATCACAAAACTAAACTTTTATGTAAAAACCTATCATCCCTTT 6259
ref|NT_099026.1|MmY_98663_33_c      ATAATTTAATGATTCTAACACATTTTCACCTCCATTCCATT--TCCCCTC 6277
                                     ***   *** *    *  * * *** *  *  *  **  *  **** *

ref|NT_099035.1|MmY_98672_33_5      CTACTTTGAATCCTCAGGGTGCACATCTGTTCATTAACCATGTTTTTCTT 6280
ref|NT_078929.2|MmY_78994_33_4      CTACTTTGAATCCTCAGGGTGCACATCTGTTCATTAACCATGTTTTTCTT 6294
ref|NT_099035.1|MmY_98672_33_c      CTACTTTGAATCCTCAGGGTGCACATCTGTTCATTAACCATGTCTTTCTT 6309
ref|NT_099026.1|MmY_98663_33_c      ATTTTCTGCTTCTTCATATGATATTGCAATCTATTAAA-ATACAGTACTG 6326
                                     *  * **  ** ***      *   *  *  *****  **    * **

ref|NT_099035.1|MmY_98672_33_5      AAATCATATCAGGAATCAGAATGAGGAAATAGGTACAAGGAAATAATAAT 6330
ref|NT_078929.2|MmY_78994_33_4      AAATCATATCAGGAATCAGAATGAGGAAATAGGTACAAGGAAATAATAAT 6344
ref|NT_099035.1|MmY_98672_33_c      AAATCATATCAGGAATCAGAATGAGGAAATAGGTATAAGGAAATAATAAT 6359
ref|NT_099026.1|MmY_98663_33_c      AAGGCAGAGGGAGGATCAGTGTGGGAA----GCTGCAAAGTTCTT-TAAT 6371
                                    **  ** *    * *****  ** * *    * *  ** *   *  ****

ref|NT_099035.1|MmY_98672_33_5      CATCTGAACACCAGCCTGACTTT----GAAAAAAAAAAGGCATGTTTTTT 6376
ref|NT_078929.2|MmY_78994_33_4      CATCTGAACACCAGCCTGACTTT----GAAAAAAAAAAGGCATGTTTTTT 6390
ref|NT_099035.1|MmY_98672_33_c      CATCTGAACACCAGCCTGACTTTTGAAAAAAAAAAAAAGGCATGCTTTTC 6409
ref|NT_099026.1|MmY_98663_33_c      TCTA-AAACAACAGATACACTGTC---TGAATATTCCAGATACAACTTTT 6417
                                      *   **** ***    *** *      ** *    **  *    ***

ref|NT_099035.1|MmY_98672_33_5      GTAGTAATTCTT--TTCAAGATTGTGGTTGAAGTATTAATC-GCATCAGC 6423
ref|NT_078929.2|MmY_78994_33_4      GTAGTAATTCTT--TCCAAGATTGTGGTTGAAGTATTAATC-GCATCAGC 6437
ref|NT_099035.1|MmY_98672_33_c      GTAGTAATTCTT--TCCAAGATCGTGATTGAAGTATTAATCTGAATCAGC 6457
ref|NT_099026.1|MmY_98663_33_c      -TGGTAACTTTGGGTCTACTGTTAGCATGAAATTTCTAGAAAAAAAAAAC 6466
                                     * **** * *   *  *   *     *  ** *  **      *  * *

ref|NT_099035.1|MmY_98672_33_5      TGCAATCCTTGAG--AAAGTTCGATCTAAATAACACTGTGGGTGGCAATG 6471
ref|NT_078929.2|MmY_78994_33_4      TGCAATCCTTGAG--AAAGTTCGATCTAAATAACACTGTGGGTGGCAATG 6485
ref|NT_099035.1|MmY_98672_33_c      TGCAATCCTTGAG--AAAGTTAGATCTAACTAACACTGTGGGTGGCAATG 6505
ref|NT_099026.1|MmY_98663_33_c      TTAAAGAATTAAGCCATGGTTCCTCTGGACCAGAGAGTTTAGTATCACTA 6516
                                    *  **   ** **  *  ***       *  *      *  **  ** *

ref|NT_099035.1|MmY_98672_33_5      ATATTGCCCAGTGAGGGGCTGGAACCCCCCTTAAATTTTCATACAATCCA 6521
ref|NT_078929.2|MmY_78994_33_4      ATATTGCCCAGTGAGGGGCTGGAACCCCCCTTAAATTTTCATACAATCCA 6535
ref|NT_099035.1|MmY_98672_33_c      ATATTGCCCAGTGAGGGGCTGGAACCCCCCTTAAATTTTCATACAATCCA 6555
ref|NT_099026.1|MmY_98663_33_c      ATGAGTCATATTCATG---TCAATCATTTTCATCACTGTTACAAGATGCC 6563
                                    **    *  * * * *   *  * *         * * * * *  ** *

ref|NT_099035.1|MmY_98672_33_5      TAGATTATGAGGAATGTGATGTGAGGGCAGCAAGTAGGGAGAAACTCTAC 6571
ref|NT_078929.2|MmY_78994_33_4      TAGATTATGAGGAATGTGATGTGAGGGCAGCAAGTAGGGAGAAACTCTAC 6585
ref|NT_099035.1|MmY_98672_33_c      TAGATTATGAGGGATGTGATGTGAGGGCAGCAAGTAGAGAGAAACTCTAC 6605
ref|NT_099026.1|MmY_98663_33_c      TAG-CAACAAGGAAAACAGGTCCATGGTCACAAG-AGTATGAAA------ 6605
                                    ***   *  *** *         * **   **** **   ****

ref|NT_099035.1|MmY_98672_33_5      AACAGCATGAGTGGTAACAGAGTGGTACCAGCTTTTAAGTGTTTAAGAGT 6621
ref|NT_078929.2|MmY_78994_33_4      AACAGCATGAGTGGTAACAGAGTGGTACCAGCTTTTAAGTGTTTAAGAGT 6635
ref|NT_099035.1|MmY_98672_33_c      AACAGCATGAGTGGTAACAGAGTGGTACCAGCTTTTAAGTGTTTAAGAGT 6655
ref|NT_099026.1|MmY_98663_33_c      -GGGGCATGAATG--AGCACAATTGT-CCAATTTATTGGCAGCCAGTAAA 6651
                                        ****** **  * ** * * ** ***  ** *  *     *  *

ref|NT_099035.1|MmY_98672_33_5      GGTAAGAGCTTTTAAGTGGTTCACCTCACCAAGAATCCATCAAAGCCCTG 6671
ref|NT_078929.2|MmY_78994_33_4      GGTAAGAGATTTTAAGTGGTTCACCTCACCAAGAATCCATCAAAGCCCTG 6685
ref|NT_099035.1|MmY_98672_33_c      GGTAAGAGCTTTTAAGTGGTTCACCTCACCAAGAATCCATCAAAGCCCAG 6705
ref|NT_099026.1|MmY_98663_33_c      CATCA-ACACAGGAAGCGACCAAATACAATAAACACCTTCCAAGAC---A 6697
                                      * * *      *** *    *   **  **  * *   ***  *
```

```
ref|NT_099035.1|MmY_98672_33_5      TATTTTGGAGGTAAAATGTACATTCTGGGGAGCCCCTCTGCCATGCTTAA 6721
ref|NT_078929.2|MmY_78994_33_4      TATTTTGGAGGTAAAATGTACATTCCTGGGAGCCCCTCTGCCATGCTTAA 6735
ref|NT_099035.1|MmY_98672_33_c      TATTTTGGAGGTAAAATGTACATTCCTGGGAGCCCCTCTGCCATGCTTAA 6755
ref|NT_099026.1|MmY_98663_33_c      TGTTCCCAATG-ACCACGTTCCTT--TACAAGTTTCTCACCCCTTCCATG 6744
                                    * **    * * *  * ** * **      **   ***  ** * *

ref|NT_099035.1|MmY_98672_33_5      TATTCTAATAAAATTAAAAATAATATTAATTTCTCATTTAATAGACCCTG 6771
ref|NT_078929.2|MmY_78994_33_4      TATTCTAATAAAATTAAAAATAATATTAATTTCTCATTTAATAGACCCTG 6785
ref|NT_099035.1|MmY_98672_33_c      TATTCTAGTAAAATTAAAAATAATATTAATTTCTCATTTAATAGACCCTG 6805
ref|NT_099026.1|MmY_98663_33_c      CACACCCATCAATCAACGCATAGATTAAAGCCCTCA--GGGTCCAATCAC 6792
                                     *  *   * **   *   ***   * **   ****     *  *  *

ref|NT_099035.1|MmY_98672_33_5      CTTACAGTCCCTCCAAATTTTACTCCCCACGGTTCTTTTACTACCTCCCA 6821
ref|NT_078929.2|MmY_78994_33_4      CTTACAGTCCCCCCAAATTTTACTCCCCACGGTTCTTTTACTACCTCCCA 6835
ref|NT_099035.1|MmY_98672_33_c      CTTACAGTCCCCCCAAATTTTACTCCCCACGGTTCTTTCACTACCTCCCA 6855
ref|NT_099026.1|MmY_98663_33_c      TTTTCAGATTGAGTATATTGTTCTACCTATGAACATTGTACTTCGGGCCA 6842
                                     ** ***       * *** * ** ** * *    **  *** *   ***

ref|NT_099035.1|MmY_98672_33_5      ---TCCTCTCCAGTTCCTCC------CCCTTTCGGTCATCATTCATCAAA 6862
ref|NT_078929.2|MmY_78994_33_4      ---TCCTCTCCAGTTCCTCC------CCCTTTCCGTCATCATTCATCAAA 6876
ref|NT_099035.1|MmY_98672_33_c      ---TCCTCTCCAGTTCCTCC------ACCTTTCGGTCATCATTCATCAAA 6896
ref|NT_099026.1|MmY_98663_33_c      AAACCTTCATCAAAGACCCAGGACAGACATTTCAAACCCATTTCACCACA 6892
                                        * **  **    * *        * ****   *    **** ** *

ref|NT_099035.1|MmY_98672_33_5      TATTTCTCTTTCAAATATCTCTTCCTG-----CATTTCCTTATTCAACCC 6907
ref|NT_078929.2|MmY_78994_33_4      TACTTCTCTTTCAAATATCTCTTCCTG-----CATTTCCTTATTCAACCC 6921
ref|NT_099035.1|MmY_98672_33_c      TACTTCTCTTTCAAATATCTCTTTCTG-----CATTTCCTTATTCAACCC 6941
ref|NT_099026.1|MmY_98663_33_c      GCATTCTTTATGCTACACAAAGTGATAGGATCTATCTGTCTCTTTAAGGG 6942
                                       **** * *   * *     *  *       ** *   * ** **

ref|NT_099035.1|MmY_98672_33_5      CATCTCCCTCCACCA--TGCCACCTGATCTTCTCCTTCTTGTCCACCTTT 6955
ref|NT_078929.2|MmY_78994_33_4      CATCTCCCTCCACCA--TGCCACCTGATCTTCTCCTTCTTGTCCACCTTT 6969
ref|NT_099035.1|MmY_98672_33_c      CATCTCCCTCCACCA--TGCCACCTGATCTTCTCCTTCTTGTCCACCTTT 6989
ref|NT_099026.1|MmY_98663_33_c      TATCTCTATCAAGCAGTTCCAAAAGGAAAATGTCCAGGTAAACAGCATCA 6992
                                     *****  ** * **  * * *   **   * ***   *   *  * *

ref|NT_099035.1|MmY_98672_33_5      GTACACTAATAAA-TCCATTCTATTTCTCCATACCATGGGCATCCATGTA 7004
ref|NT_078929.2|MmY_78994_33_4      GTACACTAATAAA-TCCATTCTATTTCTCCATACCATGGGCATCCATGTA 7018
ref|NT_099035.1|MmY_98672_33_c      GTCCACTAATAAAATCCATTCTATATCCCCATACCATGGGCATCCATGTA 7039
ref|NT_099026.1|MmY_98663_33_c      GCACCTCCAGGAG--CTTTTATAAATGTGAGT-TCAAGAAGAGCAAAAT- 7038
                                    *  *    *  *   *  ** **  *     *  ** *   * * *  *

ref|NT_099035.1|MmY_98672_33_5      TGCACCCAAGTCC-TTCCTCTATACCTAACTTCACTGGATCTACAGATTG 7053
ref|NT_078929.2|MmY_78994_33_4      TGCACCCAAGTCC-TTCCTCTATACCTAACTTCACTGGATCTACAGATTG 7067
ref|NT_099035.1|MmY_98672_33_c      TGCACCCAAGTCCCTTCCTCTATACCTAACTTCACTGGATCTACAGATTG 7089
ref|NT_099026.1|MmY_98663_33_c      TGCAATGAAATTTAAGGGTCTTTGTCCCGCATCTCTGCATCTTAAAACCA 7088
                                    ****   ** *       *** *  *   * ** *** ****  * *

ref|NT_099035.1|MmY_98672_33_5      TAGACAGGCCATCACATATGTAATGTTGAATGTCTTAATGTTTCTATTGC 7103
ref|NT_078929.2|MmY_78994_33_4      TAGACAGGCCATCACATATGTAATGTTGAATGTCTTAATGTTTCTATTGC 7117
ref|NT_099035.1|MmY_98672_33_c      TAGACAGGCCATCACATATGTAATGTAGAATGTGTTAATCTTTCTATTGC 7139
ref|NT_099026.1|MmY_98663_33_c      TAGGGTGAACA-CAGAAATGTAG-GTCAAAAGTGTGAATTTCACCTATAC 7136
                                    ***   *  ** ** * *****  **  ** ** * *** *  *   * *

ref|NT_099035.1|MmY_98672_33_5      TGTCACCAAAAACTATAAC--CCAAGCACAAGTTGGGGAGGGTAGGACTT 7151
ref|NT_078929.2|MmY_78994_33_4      TGTCACCAAAAACTATAAC--CCAAGCACAAGTTGGGGAGGGTAGGACTT 7165
ref|NT_099035.1|MmY_98672_33_c      TGTCACCAAAAACTATAAC--CCAAGCACAAGTTTGGGAGGGTAGGACTT 7187
ref|NT_099026.1|MmY_98663_33_c      TACAACTAATTGGGATGATTTCTAGCCATCTCTACGTTCTGTCTGTTACG 7186
                                    *   ** **     ** *   * *  **    *  *    *   *
```

```
ref|NT_099035.1|MmY_98672_33_5      ATTTGCAATGTGGGTACTGTAAGGAAAAGAGACACTGAGCAAGAGACTGC 7201
ref|NT_078929.2|MmY_78994_33_4      ATTTGCAATGTGGGTACTGTAAAGAAAAGAGACACTGAGCAAGAGACTGC 7215
ref|NT_099035.1|MmY_98672_33_c      ATTTGGTTTACAC-TTCTTCATTGCTATTCATCATCTGAAGACAGACAGA 7236
ref|NT_099026.1|MmY_98663_33_c      GAGTCCACCAAGTCTGCCTCAAGGCATATTG-CATAGTGTGAAATAGTGC 7235
                                       *          * *   *  *        **       * * *  *

ref|NT_099035.1|MmY_98672_33_5      CCCAAGAAAGAGACAGATACCAGACCACCTAAGTCCTTTTCTCAGAGGAT 7251
ref|NT_078929.2|MmY_78994_33_4      CCCAAGAAAGAGACAGATACCAGACCACCTAAGTCCTTTTCTCAGAGGAT 7265
ref|NT_099035.1|MmY_98672_33_c      AC--AGGAACTGAAATGGGCCAGGAACCTAGAGGCAGGAGCT---AATAC 7281
ref|NT_099026.1|MmY_98663_33_c      CAGCAGTTGATTGCAAACAACACTATGTGTTGACTTGCATGT---AAAAT 7282
                                        **        *     **                   *   *  *

ref|NT_099035.1|MmY_98672_33_5      GATGACTACATGATATGGGGAA---CAGACAC-CCTACGTGACACTAGGG 7297
ref|NT_078929.2|MmY_78994_33_4      GATGACTACATGATATGGGGAA---CAGACAC-CCTACGTGACACTAGGG 7311
ref|NT_099035.1|MmY_98672_33_c      AGAGACCA---GGGATGGGTG----CTCCTGC-CACGCACACCAGTCGAG 7323
ref|NT_099026.1|MmY_98663_33_c      GAGACATGTATAATATAGACACATTTGTACATACACACATACTCACAGAA 7332
                                                  ** *               *   *         *

ref|NT_099035.1|MmY_98672_33_5      TAACCCCAGCTGTGGAAGAGACCCTTATGGCTTCCCTTCATGATATC-AG 7346
ref|NT_078929.2|MmY_78994_33_4      TAACCCCAGCTGTGGAAGAGACCCTTATGGCTTCCCTTCATGATATC-AG 7360
ref|NT_099035.1|MmY_98672_33_c      TCAGCTTGACAGGCCTAGAGGCCTGGAAG-----CACTCATGA------- 7361
ref|NT_099026.1|MmY_98663_33_c      AGAGGCAAGCAGTAGAGGAGCTTTGTCTTAGGAACCCTCATGGAAGAGAT 7382
                                      *      * *     ***              *  *****

ref|NT_099035.1|MmY_98672_33_5      AGCAGAGTTTTT---------AGTCCTGACAGAGCCACTGGAGAAATTGA 7387
ref|NT_078929.2|MmY_78994_33_4      AGCAGAGTTTTT---------AGTCCTGACAGAGCCACTGGAGAAATTGA 7401
ref|NT_099035.1|MmY_98672_33_c      GGCAAAGAGTTT---------CACGGAAGCTCACCTTCTGGAGTTTTGGC 7402
ref|NT_099026.1|MmY_98663_33_c      GGTAGAAATCTTGTAAGGCAGAGAGGTCACTGAGACACTGTCTCTACTGC 7432
                                     * * *    **                 *  *    ***        *

ref|NT_099035.1|MmY_98672_33_5      GGACCCAGAAATCCTTAGTATTCGGGGCTACTGGTCAGAAACCATACTCT 7437
ref|NT_078929.2|MmY_78994_33_4      GGACCCAGAAATCCTTAGTATTCGGGGCTACTGGTCAGAAACCATACTCT 7451
ref|NT_099035.1|MmY_98672_33_c      CTTCTCATTAACCC---ATATACTAATCCCCTATTCCCGCGTTAG---TC 7446
ref|NT_099026.1|MmY_98663_33_c      ACAAAATCAGGCCAGTCTTCCTTGCATCCCTAAGTGGTATGGTATCACTA 7482
                                                *     *        *      *        *

ref|NT_099035.1|MmY_98672_33_5      TAGACCA--CTGATAAGAAAGTTAATCTATGAATAAGCCAAGTAACTCAT 7485
ref|NT_078929.2|MmY_78994_33_4      TAGACCA--CTGATAAGAAAGTTAATCTATGAATAAGCCAAGTAACTCAT 7499
ref|NT_099035.1|MmY_98672_33_c      TGGTGTA--TGGATCCACGTGCTCTCTTTTAGTGTTATCTTATTATAAGT 7494
ref|NT_099026.1|MmY_98663_33_c      TCCATCATCCCCATCTGAGAATATATGGACTATTGATGAAGGGTGGAGAA 7532
                                    *     *     **

ref|NT_099035.1|MmY_98672_33_5      TCCTCCCTCATCATTCCTCAAGCCCCACCACATTGTTG---TGGAGAGAC 7532
ref|NT_078929.2|MmY_78994_33_4      TCCTCCCTCATCATTCCTCAAGCCCCACCACATTGTTG---TGGAGAGAT 7546
ref|NT_099035.1|MmY_98672_33_c      TCTTTTTAAGATTGAATTCTGATATAGCTAAGCTTTCG---CCA----GT 7537
ref|NT_099026.1|MmY_98663_33_c      TCAGTTTTCATCAAAGGTAAGGCTTTTGTATGTGATAGGTTTGCCATGCT 7582
                                    **               *           *     * *

ref|NT_099035.1|MmY_98672_33_5      ATACTAG------CATATTTAAAATCCCAAATAAGCTGCTCTTTGGAAGG 7576
ref|NT_078929.2|MmY_78994_33_4      ATACTAG------CATATTTAAAATCCCAAATAAGCTGCTCTTTGGAAGG 7590
ref|NT_099035.1|MmY_98672_33_c      GTTCCAA------TATCCTAGAAAGTCCT--TGAGCTGACCATGGGCTTG 7579
ref|NT_099026.1|MmY_98663_33_c      CTACTGGTAGCCCCATGTCCAAGAGTTTA--TGGGCAACACAATTGATTT 7630
                                     * *          **     * *       *  **    *    *

ref|NT_099035.1|MmY_98672_33_5      AACTTCAATC----CCTTGGCAAGCCTGTCCC-TATAAATTTAACTTTAA 7621
ref|NT_078929.2|MmY_78994_33_4      AACTTCAATC----CCTTGGCAAGCCTGTCCC-TATAAATTTAACTTTAA 7635
ref|NT_099035.1|MmY_98672_33_c      GATTTCAGTAAGAATGTTGCCTATTCAATGGCATTCAGAATTGCCTCTGG 7629
ref|NT_099026.1|MmY_98663_33_c      GAATTGGGTTGTATCAAAGGGGAGAAAAGTAGACTTGAACTTGGGAGGAT 7680
                                     * **   *         *   *               * **
```

```
ref|NT_099035.1|MmY_98672_33_5      AATTAGAAGAAGAATATAGATTACATGAGTCTCAA--GCCCCGTCAAGAA 7669
ref|NT_078929.2|MmY_78994_33_4      AATTAGAAGAAGAATATAGATTACATGAGTCTCAA--GCCCCGTCAAGAA 7683
ref|NT_099035.1|MmY_98672_33_c      TATTATACTACCCCTTTGAATAAAAGCTAAGTCAGT-GCCCTACACAGAA 7678
ref|NT_099026.1|MmY_98663_33_c      GATTAAGGTGGGAATGGGTCTGGTTTTGGTCTGGAGGAGTTATCAAGAG 7730
                                     ****         *     *          *     *        ***

ref|NT_099035.1|MmY_98672_33_5      AAGTACTAGAATGGACCTATTTAGAGAGATATCCAACAGCCT--GGGCAG 7717
ref|NT_078929.2|MmY_78994_33_4      AAGTACTAGAATGGACCTATTTAGAGAGATATCCAACAGCCT--GGGCAG 7731
ref|NT_099035.1|MmY_98672_33_c      ATTTATCATCAT----CTAGGAAGAAGGAAGTTTGAGACCTA--AGGAGG 7722
ref|NT_099026.1|MmY_98663_33_c      AATTGG-AAAGTGAATTCCATCAAAAAATTGTATGAAGTTCTCAAGGAAT 7779
                                    *  *   *   *          * *      *   *         **

ref|NT_099035.1|MmY_98672_33_5      AGACTGGTGGTATAATTGAGACAA-AAATGG-TGTCCCCCCCCATCATTG 7765
ref|NT_078929.2|MmY_78994_33_4      AGACTGGTGGTATAATTGAGACAA-AAATGG-TGTCCCCCCC-ATCATTG 7778
ref|NT_099035.1|MmY_98672_33_c      AAGCAGAGTACATACTTAGAACTATAGATAGCTGAGTTCCACCCATATAC 7772
ref|NT_099026.1|MmY_98663_33_c      TAATTAATGTAGTTCTTAATATAC--AATGTCTGTACTGAACATATACAG 7827
                                                *  **   *      **   **       *    *

ref|NT_099035.1|MmY_98672_33_5      -----TGACATTGAAAACTGGTGAG-----ATTCCTTTCGGGGTACA-AC 7804
ref|NT_078929.2|MmY_78994_33_4      -----TGACATTGAAAACTGGTGAG-----ATTCCTTTTGGGGTACA-AC 7817
ref|NT_099035.1|MmY_98672_33_c      -----ACGTATTTACAATGGCCTAG-----GGGGAAGGTGGTCTATA--C 7810
ref|NT_099026.1|MmY_98663_33_c      ACCACTTATATTTCAATCTCCTAAATAGCAACTCATTTTATAGCACTTAC 7877
                                             ***   *       *                    *    *

ref|NT_099035.1|MmY_98672_33_5      AATACACCATGAGCAAAG--AGGCCAGTGAGGGCATCAGACCTC--ACCT 7850
ref|NT_078929.2|MmY_78994_33_4      AATACACCATGAGCAAAG--AGGCCAGTGAGGGCATCAGACCTC--ACCT 7863
ref|NT_099035.1|MmY_98672_33_c      AATAGACTA-AAATAGGA--ACTATAGCAAGGTCACGAA-------GCCA 7850
ref|NT_099026.1|MmY_98663_33_c      ATTACATTACGTGCAAAAGTAATCCAGAGATTATTTAAAATAAAGAAGGA 7927
                                    * ** *  *     *     *    **  *       *

ref|NT_099035.1|MmY_98672_33_5      TCAAAGGCT----CTTAGAACTAGGCGTGTTTGGTCCTTTGTCAGTCTTC 7896
ref|NT_078929.2|MmY_78994_33_4      TCAAAGGCT----CTTAGAACTAGGCGTGTTTGGTCCTTTGTCAGTCTTC 7909
ref|NT_099035.1|MmY_98672_33_c      TTGACTTCT----TCTAAGACTATG------TGGATCTTTGGTGGAGTTC 7890
ref|NT_099026.1|MmY_98663_33_c      TCAGGGGATATGGCCCAGAGACAATGCCATGTGTGTGTTCAAACATTCAC 7977
                                    *       *       *     *        **    **          *

ref|NT_099035.1|MmY_98672_33_5      CTGGAATACTCTGTTGCTACCAGTTAAAAAGCAAGGCTTTGCCACCTGGC 7946
ref|NT_078929.2|MmY_78994_33_4      CTGGAATACTCTGTTGCTACCAGTTAAAAAGCAAGGCTTTGCCACCTGGC 7959
ref|NT_099035.1|MmY_98672_33_c      TTTTTGATCCCAGTTGTTAACAGTGAA---------TTTTATC-CCAGGT 7930
ref|NT_099026.1|MmY_98663_33_c      CTACAACAC-CAGTGCTTATGATTTTGG----GAGGGGCAGAGACAGGAA 8022
                                     *      * * **   **  * *                    *  *

ref|NT_099035.1|MmY_98672_33_5      ACTCCCTAATAATCCCCCCTTACTAAAGAGACTGGGACTTTCTCATGGAC 7996
ref|NT_078929.2|MmY_78994_33_4      ACTCCCTAATAATCCCCCCTTACTAAAGAGACTGGGACTTTCTCATGGAC 8009
ref|NT_099035.1|MmY_98672_33_c      GTTTCCTGTGAGACCTTCTGAGTTTTTATTCCTCCGTTTTCTGTAAGAAT 7980
ref|NT_099026.1|MmY_98663_33_c      GATGACTGTGGCTTCCTGAAAGCCAGCCTCACTACCAGCTCAGCAAGAAA 8072
                                      *  **       *                **      *    * * *

ref|NT_099035.1|MmY_98672_33_5      CCAATATATGGGGAGGCCTTTAAAAAGATTAAAAAATCGTGCTTAGTGC- 8045
ref|NT_078929.2|MmY_78994_33_4      CCCATATATGGGGAGGCCTTTAAAAAGATTAAAAGATCGTGCTTAGTGC- 8058
ref|NT_099035.1|MmY_98672_33_c      CCAGTA---------ACCTCTTTGTACCTT----GCTTGTGTGTCACCC- 8016
ref|NT_099026.1|MmY_98663_33_c      CCCAGTCACAAGGGGATAAGTTATGGAGGAAAAGAGTAGATCACACTATA 8122
                                    **                  *               *  *

ref|NT_099035.1|MmY_98672_33_5      CTCAGCCCTAGTCCTGACAGATGTGACCGAGCCATTCACTCT-CTATTTA 8094
ref|NT_078929.2|MmY_78994_33_4      CTCAGCCCTAGTCCTGACAGATGTGACCGAGCCATTCACTCT-CTATTTA 8107
ref|NT_099035.1|MmY_98672_33_c      AACTTCCCTATTCTCTTC---TGT-ATAAAAAGTTTGATGCT-CAATTTG 8061
ref|NT_099026.1|MmY_98663_33_c      CCCTTCCCTAGCCTCTGAACTTAC--CTGTACAGTTCTCTATGCAGACTC 8170
                                      *  *****  *        *            **     * *    *
```

```
ref|NT_099035.1|MmY_98672_33_5      G-ATAAAAGAG-CAGAAATAGTGAGATAGGTCCTTAACTAAGCTCTGGGG 8142
ref|NT_078929.2|MmY_78994_33_4      G-ATAAAAGAG-CAGAAATAGTGAGATAGGTCCTGAACTAAGCTCTGGGG 8155
ref|NT_099035.1|MmY_98672_33_c      ACACATTACATTCAGATTCTACACAACCTCTCCTCTGTGTATCTGTCTAT 8111
ref|NT_099026.1|MmY_98663_33_c      ACATAAATATGATTAAAATATGCAAGAGGTTATTTTGCATAGCTTATATT 8220
                                      * *          *              *  *      * **

ref|NT_099035.1|MmY_98672_33_5      ACATAGAAAAGAC-TTGTGGCCTACTTGTAAAAAAAAAAAAAAATAGACCC 8191
ref|NT_078929.2|MmY_78994_33_4      ACATAGAAAAGAC-TTGTGGCCTACTTGTAAAAAAAAAAAAAAATAGACCC 8204
ref|NT_099035.1|MmY_98672_33_c      CATTTCATCCAAT-GCTTTGCCCACCTGTGACTAGAGATCCGTTCCACGC 8160
ref|NT_099026.1|MmY_98663_33_c      CAAAAGCAATGTTGTTTTAGACTAGAAACCTGAGGACTTTATATCATTAT 8270
                                                     * * * *           *       *

ref|NT_099035.1|MmY_98672_33_5      AATGCCCAGTGGATGGCCGTCTTTCTTAAAAGATATCAATACTTCTCAAA 8241
ref|NT_078929.2|MmY_78994_33_4      AATGCCCAGTGGATGGCCGTCTTTCTTAAAAGATATCAATACTTCTCAAA 8254
ref|NT_099035.1|MmY_98672_33_c      A--GACAA-AGGTGCCCAGAATGTCTGCAGCATTCTGCTTAGTGCCTAG- 8193
ref|NT_099026.1|MmY_98663_33_c      AGTTCCTGGAGCAAACTCCCAAGATATAGATGATGGGTATATCTTCTTAC 8320
                                    *    *           *        *  *  **

ref|NT_099035.1|MmY_98672_33_5      GATGCTGACAAACTGACTCTAGGTCAAGGAACTATAGTAGTAGCCCCCCT 8291
ref|NT_078929.2|MmY_78994_33_4      GATGCTGACAAACTGACTCTAGGTCAAGGAACTATAGTAGTAGCCCCCCT 8304
ref|NT_099035.1|MmY_98672_33_c      --------------------------------------------------
ref|NT_099026.1|MmY_98663_33_c      TGTGC----AAAGGAAACCTCAGTTAAGTGTGTGTGTCAGTGACTCCAAA 8366

ref|NT_099035.1|MmY_98672_33_5      CTGCCCCCATGCCTTAGAAAGCATTGTTAGGCAGCTGCCAGATTGCTGGA 8341
ref|NT_078929.2|MmY_78994_33_4      CTGCCCCCATGCCTTAGAAAGCATTGTTAGGCAGCTGCCAGATTGCTGGA 8354
ref|NT_099035.1|MmY_98672_33_c      --------------------------------------------------
ref|NT_099026.1|MmY_98663_33_c      CAGCTTTCCTCCTCTATGTTCCTTTATATAGCA-----TAGAATGATCTG 8411

ref|NT_099035.1|MmY_98672_33_5      TGACTAATGCTCACAGGACCCATTATCAGAGCCTCTTTCTGACTGAGAGA 8391
ref|NT_078929.2|MmY_78994_33_4      TGACTAATGCTCACAGGACCCATTATCAGAGCCTCTTTCTGACTGAGAGA 8404
ref|NT_099035.1|MmY_98672_33_c      --------------------------------------------------
ref|NT_099026.1|MmY_98663_33_c      TAAATAATGAAAATATGAACG-----CAAAGCTTTTTTGTGATTTAAGGC 8456

ref|NT_099035.1|MmY_98672_33_5      GTGTAGAGAGAGTGTAATTTGCACCCCTGCTATCCTGAATCCTTCCACCT 8441
ref|NT_078929.2|MmY_78994_33_4      GTGTAGAGAGAGTGTAATTTGCACCCCTGCTATCCTGAATCCTTCCACCT 8454
ref|NT_099035.1|MmY_98672_33_c      --------------------------------------------------
ref|NT_099026.1|MmY_98663_33_c      TTGTGTTAAAATCATA-TTTATAAC---ATTGTATTAAAT-----GGAAG 8497

ref|NT_099035.1|MmY_98672_33_5      TGCTGCCTGAAGCTGACAACTCCCTACCAGTGAAGGTGTATAGACATCCT 8491
ref|NT_078929.2|MmY_78994_33_4      TGCTTCCTGAAGCTGACATCTCCCTACCAGTGAAGGTGTATAGACATCCT 8504
ref|NT_099035.1|MmY_98672_33_c      --------------------------------------------------
ref|NT_099026.1|MmY_98663_33_c      TGAAATCTTCAGCTCTCAGGAACAATACAGTCAAT-CCTATAGGCTTCTG 8546

ref|NT_099035.1|MmY_98672_33_5      GGCAGAAGAAACCAAAACTAAGAAAGAAGTCATTGACCAGCCATGGTCAG 8541
ref|NT_078929.2|MmY_78994_33_4      GGCAGAAGAAACCAAAACTAAGAAAGAAGTCATTGACCAGCCATGGTCAG 8554
ref|NT_099035.1|MmY_98672_33_c      --------------------------------------------------
ref|NT_099026.1|MmY_98663_33_c      TTTTG-------CTCCATTGTGAGCAAA---ATTAATTGCACATCAACAG 8586

ref|NT_099035.1|MmY_98672_33_5      GTTGCCCCAATTGGTACACAGATGGTATCAGCTTTTTGATTGAAGGTAAA 8591
ref|NT_078929.2|MmY_78994_33_4      GTTGCCCCAATTGGTACACAGATGGTATCAGCTTTTTGATTGAAGGTAAA 8604
ref|NT_099035.1|MmY_98672_33_c      --------------------------------------------------
ref|NT_099026.1|MmY_98663_33_c      GTCCCACACTTTTAACTAAATCAGGGAGAAGGGCTATGTTTCCGTACAAA 8636
```

```
ref|NT_099035.1|MmY_98672_33_5      AGAAAAGTGGTGCCAGCAGTGGTCAACGGAAAAGAAACC-ATCCGTGTAA 8640
ref|NT_078929.2|MmY_78994_33_4      AGAAAAGTGGTGCCAGCAGTGGTCAACGGAAAAGAAACC-ATCCGTGTAA 8653
ref|NT_099035.1|MmY_98672_33_c      --------------------------------------------------
ref|NT_099026.1|MmY_98663_33_c      T---AAGTAATGTCAAT-GTGATTATTCAAGTAGAAATCTGGCAGGGTTA 8682

ref|NT_099035.1|MmY_98672_33_5      GCAGCTTTACTGAAGAAATGACAGTCCAGAGAGAAGAGCTAATCAC-ACA 8689
ref|NT_078929.2|MmY_78994_33_4      GCAGCTTTACTGAAGAAATGACAGTCCAGAGAGAAGAGCTAATCAC-ACA 8702
ref|NT_099035.1|MmY_98672_33_c      --------------------------------------------------
ref|NT_099026.1|MmY_98663_33_c      GGATGCGTTTGGCTTATGTGATAGTGATGACTGATTAGTCTCTTGTTATG 8732

ref|NT_099035.1|MmY_98672_33_5      TACCCAAGCCTTGCAGCTGGCCATAGAAAAGAACATCAATATCTAAACAG 8739
ref|NT_078929.2|MmY_78994_33_4      TACCCAAGCCTTGCAGCTGGCCATAGAAAAGAACATCAATATCTAAACAG 8752
ref|NT_099035.1|MmY_98672_33_c      --------------------------------------------------
ref|NT_099026.1|MmY_98663_33_c      CAAATAACCCTTACCTCTG-----AGAAAAGACAAGTAATGATATAAAAT 8777

ref|NT_099035.1|MmY_98672_33_5      ACAGCATGTA-TGCCTTTGCCACATGCATGGTAGTATCTGTAGAAAAAGG 8788
ref|NT_078929.2|MmY_78994_33_4      ACAGCATGTA-TGCCTTTGCCACATGCATGGTAGTATCTGTAGAAAAAGG 8801
ref|NT_099035.1|MmY_98672_33_c      --------------------------------------------------
ref|NT_099026.1|MmY_98663_33_c      ATACTATGAAGTGACATGGACACTTAT-TACTAGATTTCATTTTC----- 8821

ref|NT_099035.1|MmY_98672_33_5      ATGCTACTCACTTCTACTGGGGAAGATATTAAAAATAAAAAGGAAACCTT 8838
ref|NT_078929.2|MmY_78994_33_4      ATGCTACTCACTTCTACTGGGGAAGATATTAAAAATAAAAAGGAAACCTT 8851
ref|NT_099035.1|MmY_98672_33_c      --------------------------------------------------
ref|NT_099026.1|MmY_98663_33_c      -CTTTACATACTTTAGTTGTTTGAAAAATTGTGGCCCACAG---AAGCTC 8867

ref|NT_099035.1|MmY_98672_33_5      GAGACTATTAGAAGCCATACACTTTGCAAAAAACTGGACATCATACATTG 8888
ref|NT_078929.2|MmY_78994_33_4      GAGACTATTAGAAGCCATACACTTTGCAAAAAACTGGACATCATACATTG 8901
ref|NT_099035.1|MmY_98672_33_c      --------------------------------------------------
ref|NT_099026.1|MmY_98663_33_c      AAA--TATTCAAAGGTGTTAGTTTTCCAGGTGGTGAGATGTTAAGAA--A 8913

ref|NT_099035.1|MmY_98672_33_5      CCATTGTCACCACAAAGTCCATGAGGCCATAGTGAAGGGAAATCTGATGC 8938
ref|NT_078929.2|MmY_78994_33_4      CCATTGTCACCACAAAGTCCATGAGGCCATAGTGAAGGGAAATCTGATGC 8951
ref|NT_099035.1|MmY_98672_33_c      --------------------------------------------------
ref|NT_099026.1|MmY_98663_33_c      GAATT--------AGAATT--TGAATTTTTA-TTAAAGGAGATTTG---- 8948

ref|NT_099035.1|MmY_98672_33_5      CTGAACTGACTGGCCTAAAAAAAAATAAATGAATAAAATAAAGAACGTGA 8988
ref|NT_078929.2|MmY_78994_33_4      CTGAACTGACTGGCCTAAAAAAAA-TAAATGAATAAAATAAAGAACGTGA 9000
ref|NT_099035.1|MmY_98672_33_c      --------------------------------------------------
ref|NT_099026.1|MmY_98663_33_c      ------TCACTATTCTTTGAAATATCAAA--AATCCATCAGGCTCTCTCT 8990

ref|NT_099035.1|MmY_98672_33_5      AGACCATTATAAACTCAAAG--ATGCAGGCTTTGACTATACTCCAGAAGA 9036
ref|NT_078929.2|MmY_78994_33_4      AGACCATTATAAACTCAAAG--ATGCAGGCTTTGACTATACTCCAGAAGA 9048
ref|NT_099035.1|MmY_98672_33_c      --------------------------------------------------
ref|NT_099026.1|MmY_98663_33_c      CTCTCATTCTTAATCTCTCCTCATTCTCTCTCTATCTCTGTCTCTTTGTC 9040

ref|NT_099035.1|MmY_98672_33_5      TCAGAAATTAACAGGCAAAATACCTGATATATGTGGGTGTACTACCCGGG 9086
ref|NT_078929.2|MmY_78994_33_4      TCAGAAATTAACAGGCAAAATACCTGATATATGTGGGTGTACTACCCGGG 9098
ref|NT_099035.1|MmY_98672_33_c      --------------------------------------------------
ref|NT_099026.1|MmY_98663_33_c      TCTATCTCTGTCTCTCTC--TCTCTGTTTCAT-TCTCTGTTTCTCTCTGT 9087
```

```
ref|NT_099035.1|MmY_98672_33_5      GAGTCTCTAGGACCAAGATGGTTGCTCTGTCCTGCCCACAATAGAAAGGT 9136
ref|NT_078929.2|MmY_78994_33_4      GAGTCTCTAGGACCAAGATGGTTGCTCTGTCCTGCCCACAATAGAAAGGT 9148
ref|NT_099035.1|MmY_98672_33_c      --------------------------------------------------
ref|NT_099026.1|MmY_98663_33_c      CTCTGCTTGTTGTCTGTAGTATTGCA-TGTGAGCTCTCAGATACTGCTCT 9136

ref|NT_099035.1|MmY_98672_33_5      AACAATATGTCTCCAATCTAAACCACCTCATGCACTTGGAAATTGAGAAG 9186
ref|NT_078929.2|MmY_78994_33_4      AACAATATGTCTCCAATCTAAACCACCTCATGCACTTGGAAATTGAGAAG 9198
ref|NT_099035.1|MmY_98672_33_c      --------------------------------------------------
ref|NT_099026.1|MmY_98663_33_c      ACACATGTGTGCCTGCCTGTTGCCATGGTGTTTACCATGATGTCCTGGAG 9186

ref|NT_099035.1|MmY_98672_33_5      --GTCAAAGAAAATGTTGAGAGCTCAAACTGCAATGTTATAAGATTGCCA 9234
ref|NT_078929.2|MmY_78994_33_4      --GTCAAAGAAAATGTTGAGAGCTCAAACTGCAATGTTATAAGATTGCCA 9246
ref|NT_099035.1|MmY_98672_33_c      --------------------------------------------------
ref|NT_099026.1|MmY_98663_33_c      TCGCCTTCTGAAACTGTAAGACCGCAATTAAATATTTCATCTTATAATTT 9236

ref|NT_099035.1|MmY_98672_33_5      GATGTTGCCCAGGGGATTGTTAAT-GAATGCCAAGCATG-TGCCCTCACC 9282
ref|NT_078929.2|MmY_78994_33_4      GATGTTGCCCAGGGGATTGTTAAT-GAATGCCAAGCATG-TGCCCTCACC 9294
ref|NT_099035.1|MmY_98672_33_c      --------------------------------------------------
ref|NT_099026.1|MmY_98663_33_c      GGTGTGGTCATGTCTCACATCAATAGAACAGTAACCAAGACATCCATATT 9286

ref|NT_099035.1|MmY_98672_33_5      ATTGCTGGGCACCACAAGAACACCCCCCCC-AGAAGGTGGATAAGAGTTG 9331
ref|NT_078929.2|MmY_78994_33_4      ATTGCTGGGCACCACAAGAACACCCCCCCCCAGAAGGTGGATAAGAGTTG 9344
ref|NT_099035.1|MmY_98672_33_c      --------------------------------------------------
ref|NT_099026.1|MmY_98663_33_c      CCAGTTTATTTTTTCAATCATTCTGTACTT-----ATTTCATTAAAGTTA 9331

ref|NT_099035.1|MmY_98672_33_5      ACT---GGCCATGAGCATACTGAAAAGTAGACTTTACTGAAGTTAAACCA 9378
ref|NT_078929.2|MmY_78994_33_4      ACT---GGCCATGAGCATACTGAAAAGTAGACTTTACTGAAGTTAAACCA 9391
ref|NT_099035.1|MmY_98672_33_c      --------------------------------------------------
ref|NT_099026.1|MmY_98663_33_c      CCTTTTGTCCATTGAAAAGTTTTAAGAATACTTTTAAAGGTCCTGAACAA 9381

ref|NT_099035.1|MmY_98672_33_5      GCCAAGTACAGTAATAAATATCTATTTGTTTTTTTGTAAACACCTTTTCA 9428
ref|NT_078929.2|MmY_78994_33_4      GCCAAGTACAGTAATAAATATCTATTTGTTTTTTTGTAAACACCTTTTCA 9441
ref|NT_099035.1|MmY_98672_33_c      --------------------------------------------------
ref|NT_099026.1|MmY_98663_33_c      AT--AGTGC--CAGTAGGAGTGGCACTGTTAGTAGGTGTGGCCTTTTTGA 9427

ref|NT_099035.1|MmY_98672_33_5      GGCTTGGTCGAAGCCTTCCCCACTAATAACAAGAACACCCATGCCATGGG 9478
ref|NT_078929.2|MmY_78994_33_4      GGCTTGGTCGAAGCCTTCCCCACTAATAACAAGAACACCCATGCCATGGG 9491
ref|NT_099035.1|MmY_98672_33_c      --------------------------------------------------
ref|NT_099026.1|MmY_98663_33_c      AGTATGTGTGA------CCCTGTTGG-AGTAGATGTAGCCTTGTTGGAGG 9470

ref|NT_099035.1|MmY_98672_33_5      AAAGAAGATATTAGAGGAAAATTTCTCAAGATTTGGCATCTCTAAGGTAC 9528
ref|NT_078929.2|MmY_78994_33_4      AAAGAAGATATTAGAGGAAAGTTTCCCAAGATTTGGCATCTGTAAGGTAC 9541
ref|NT_099035.1|MmY_98672_33_c      --------------------------------------------------
ref|NT_099026.1|MmY_98663_33_c      AAATGTAACACTGTGCTTGTGGACTTTGAGATCGGCTATGCTTAAGCTGT 9520

ref|NT_099035.1|MmY_98672_33_5      TTTCATCAGACAATGGCCCCTCCTTCATTGTCCAGGTAAGCCAAGGGTTA 9578
ref|NT_078929.2|MmY_78994_33_4      TTTCATCAGACAATGGCCCCTCCTTCATTGTCCAGGTAAGCCAAGGGTTA 9591
ref|NT_099035.1|MmY_98672_33_c      --------------------------------------------------
ref|NT_099026.1|MmY_98663_33_c      CCCCAGTGTGGCACACAGTCTCCATC-TTCTACCTGTGTATCAAGATGTA 9569
```

```
ref|NT_099035.1|MmY_98672_33_5      GCCAGACAATGGGGGATTCATTGGAAGATACATTGTTCTTACAGATTCCA 9628
ref|NT_078929.2|MmY_78994_33_4      GCCAGACAATGGGGGATTCATTGGAAGATACATTGTTCTTACAGATTCCA 9641
ref|NT_099035.1|MmY_98672_33_c      --------------------------------------------------
ref|NT_099026.1|MmY_98663_33_c      ---AAACTCTCAGCTCCTTCTCTAGCATTGTGTCTGTCTGAATGCTTCCA 9616

ref|NT_099035.1|MmY_98672_33_5      GACTTCAGGACAGGTAGAGTGGATGAATAGAGCATTAAAAGAAACCTTGA 9678
ref|NT_078929.2|MmY_78994_33_4      GACTTCAGGACAGGTAGAGTGGATGAATAGAGCATTAAAAGAAACCTTGA 9691
ref|NT_099035.1|MmY_98672_33_c      --------------------------------------------------
ref|NT_099026.1|MmY_98663_33_c      TATTTCCACTCATG-ATAATAATGGAGTAAACCTCTGAAAACAAGTT--A 9663

ref|NT_099035.1|MmY_98672_33_5      CTAAATTGGTTTTAGAGACCTACAGAAAAGATTATGCAGCTCTTTTTCCC 9728
ref|NT_078929.2|MmY_78994_33_4      CTAAATTGGTTTTAGAGACCTACAGAAAAGATTATGCAGCTCTTTTTCCC 9741
ref|NT_099035.1|MmY_98672_33_c      --------------------------------------------------
ref|NT_099026.1|MmY_98663_33_c      CAAA----GTGTTCAAGAGTTACTGTGATCCTGGAGACTCTTCATGGCAA 9709

ref|NT_099035.1|MmY_98672_33_5      TTTGCCTTGTTCTGAGTATGGAACACCACAGGGAAATTTAAACTTACACC 9778
ref|NT_078929.2|MmY_78994_33_4      TTTGCCTTGTTCTGAGTATGGAACACCACAGGGAAATTTAAACTTACACC 9791
ref|NT_099035.1|MmY_98672_33_c      --------------------------------------------------
ref|NT_099026.1|MmY_98663_33_c      TAAAACCCAAACTAA-CACAAACACAAACATTCAAAAACACATTTGATAT 9758

ref|NT_099035.1|MmY_98672_33_5      ATTCAAACTCCTCAACTTGGGACGCCCCTCCCTTTAA-CAGAAGCAGGTG 9827
ref|NT_078929.2|MmY_78994_33_4      ATTCAAACTCCTCAACTTGGGACGCCCCTCCCTTTAAACAGAAGCAGGTG 9841
ref|NT_099035.1|MmY_98672_33_c      --------------------------------------------------
ref|NT_099026.1|MmY_98663_33_c      TTTGACTATTCTTGGCCTAGGGAGTGGCACTACTGGGAGCTAATAACCTT 9808

ref|NT_099035.1|MmY_98672_33_5      CAATGTTTGAACTTGATGTTTCTTTTTCAGAACCCCCTCTA-----GCTC 9872
ref|NT_078929.2|MmY_78994_33_4      CAATGTTTGAACTTGATGTTTCTTTTTCAGAACCCCCTCTA-----GCTC 9886
ref|NT_099035.1|MmY_98672_33_c      --------------------------------------------------
ref|NT_099026.1|MmY_98663_33_c      GTTGAAGTAAGTTTGGCCTTGTTGAAAGAAATTTATCTTTGTGAACATGG 9858

ref|NT_099035.1|MmY_98672_33_5      ACTTGAAGGCTCTACAATTGGTCAGAAAAGATGCCTTGGAAATGCTGAAA 9922
ref|NT_078929.2|MmY_78994_33_4      ACTTGAAGGCCCTACAATTGGTCAGAAAAAAATGCCTTGGAAATGCTGAAA 9936
ref|NT_099035.1|MmY_98672_33_c      --------------------------------------------------
ref|NT_099026.1|MmY_98663_33_c      ACTTTAATGCCCTCTGCCTAGCTGCCTGAAA-GCTAATCATTTCCTAGCA 9907

ref|NT_099035.1|MmY_98672_33_5      CACAATAAAAAACAAAAACAAAAACAAAAAAACAAAAAAACATACTAACC 9972
ref|NT_078929.2|MmY_78994_33_4      CACAATAAAAAACAAAAACAAAAACAAAAAAACAAAAAAACATACTAACC 9986
ref|NT_099035.1|MmY_98672_33_c      --------------------------------------------------
ref|NT_099026.1|MmY_98663_33_c      CCCTTCAGATGAAGATGTAGAACTCTCAGCTCCAAATGCACATGCCTGCC 9957

ref|NT_099035.1|MmY_98672_33_5      AGGA----ACCATCTTCGTGCCACATAAATTTCAAACAGAAGATCCTGTC 10018
ref|NT_078929.2|MmY_78994_33_4      AGGA----ACCATCTTCGTGCCACATAAATTTCAAACAGAAGATCCTGTC 10032
ref|NT_099035.1|MmY_98672_33_c      --------------------------------------------------
ref|NT_099026.1|MmY_98663_33_c      AGGATCCTGCCATATTCCTACCTTGATGATAATGGACTGAATTTCTGAAC 10007

ref|NT_099035.1|MmY_98672_33_5      CGGGTCCGGTGCCACCATTCTGGCAAACTTGAACTCAGATCAAAAGGATG 10068
ref|NT_078929.2|MmY_78994_33_4      CGGGTCCGGTGCCACCATTCTGGCAAACTTGAACTCAGATCAAAAGGATG 10082
ref|NT_099035.1|MmY_98672_33_c      --------------------------------------------------
ref|NT_099026.1|MmY_98663_33_c      CTG--TAAGCCAGGGTAAATTGAAAAGTGTCTCTGCTGAT-AGTTGGTTG 10054
```

```
ref|NT_099035.1|MmY_98672_33_5      CTCCCTGGTGCTTCTGACAAGCCCTGCTGCCGCACCTCCAGCATGTAACC 10118
ref|NT_078929.2|MmY_78994_33_4      CTCCCTGGTGCTTCTGACAAGCCCTGCTGCCGCACCTCCAGCATGTAACC 10132
ref|NT_099035.1|MmY_98672_33_c      --------------------------------------------------
ref|NT_099026.1|MmY_98663_33_c      CTATTCCAGAAAGACCAAAAGTTATGACGTACCATCCAGAGTCTGAGAAT 10104

ref|NT_099035.1|MmY_98672_33_5      GCTTCTGGCCTGCCATGGCCCACTGGCCTGGCACTGCTCACACACAGTGG 10168
ref|NT_078929.2|MmY_78994_33_4      GCTTCTGGCCTGCCATGGCCCACTGGCCTGGC------------------ 10164
ref|NT_099035.1|MmY_98672_33_c      --------------------------------------------------
ref|NT_099026.1|MmY_98663_33_c      ACATTGGTTTAGCTTCTAGCATTTGACAGAAAACAGGATTAACAAGATAA 10154

ref|NT_099035.1|MmY_98672_33_5      GCACTGCACAGGATCCCCCCTTCTTCCCTCCTCCCACTGGCAAGAGTCTC 10218
ref|NT_078929.2|MmY_78994_33_4      --ACTGCACAGGATCCCCCCTTCTTCCCTCCTCCCACTGGCAAGAGTCTC 10212
ref|NT_099035.1|MmY_98672_33_c      --------------------------------------------------
ref|NT_099026.1|MmY_98663_33_c      TGAGAATATAAAACTTCC---TATTTCATACTTAGAGACACATGGGTTAT 10201

ref|NT_099035.1|MmY_98672_33_5      TTTCGTCTCATTCAAGGGGCTTCTGAGGTTCTCAATCTTACAAATCCTAA 10268
ref|NT_078929.2|MmY_78994_33_4      TTTCGTCTCATTCAAGGGGCTTCTGAGGTTCTCAATCTTACAAATCCTAA 10262
ref|NT_099035.1|MmY_98672_33_c      --------------------------------------------------
ref|NT_099026.1|MmY_98663_33_c      AGATCTGCGGAACTCAAGGTTTCATGGGTGGT--ATGGTAGGGTGATTAG 10249

ref|NT_099035.1|MmY_98672_33_5      AGCTGTGACCTCAGGTTGGCTTTATTTGGCAG--CTGG-GCCAGAGGTTT 10315
ref|NT_078929.2|MmY_78994_33_4      AGCTGTGACCTCAGGTTGGCTTTATTTTGCAG--CTGG-GCCAGAGGTTT 10309
ref|NT_099035.1|MmY_98672_33_c      --------------------------------------------------
ref|NT_099026.1|MmY_98663_33_c      TGTCTTTATATGAGTGCAGTGTCTGTTCCCAAGTCTGATGTCACACATAC 10299

ref|NT_099035.1|MmY_98672_33_5      AATTTTCTAACTTATAAAATTAGAACTATGTACTAGAAGGAGTGGGAAAT 10365
ref|NT_078929.2|MmY_78994_33_4      AATTTTCTAACTTATAAAATTAGAACTATGTACTAGAAGGAGTGGGAAAT 10359
ref|NT_099035.1|MmY_98672_33_c      --------------------------------------------------
ref|NT_099026.1|MmY_98663_33_c      ACATATGTTAAGTAATGAGTAGAATATCTTCAACACTTGCTCTCTTAGA- 10348

ref|NT_099035.1|MmY_98672_33_5      GTGAGACCC-----CAACTTAGAGCATTTCTCCCTGGAAGGTGAAGCCCC 10410
ref|NT_078929.2|MmY_78994_33_4      GTGAGAACC-----CAACTTAGAGCATTTCTCCCTGGAAGGTGAAGCCCC 10404
ref|NT_099035.1|MmY_98672_33_c      --------------------------------------------------
ref|NT_099026.1|MmY_98663_33_c      GTGAAACCCTCTCACCATTTTGACTAGTT-TGACTGGCCAATACACTATT 10397

ref|NT_099035.1|MmY_98672_33_5      TGGACATTCTCCAAGCTTGTATTCCCTGAGCTCTAAAAATACAGCCAGAA 10460
ref|NT_078929.2|MmY_78994_33_4      TGGACATTGTCCAAGCTTGTATTCCCTGAGCTCTAAAAATACAGCCAGAA 10454
ref|NT_099035.1|MmY_98672_33_c      --------------------------------------------------
ref|NT_099026.1|MmY_98663_33_c      GGGATATCTACCTGTCTCCA--CCCCACAGTGTTGCAGATATGAATAGAC 10445

ref|NT_099035.1|MmY_98672_33_5      AGAAGCTCTATGTTCTCTACAGCCACCAAAAA-GCATTTTGGTTTCTGAG 10509
ref|NT_078929.2|MmY_78994_33_4      AGAAGCTCTATGTTCTCTACAGCCACCAAAAA-GCATTTTGGTTTCTGAG 10503
ref|NT_099035.1|MmY_98672_33_c      --------------------------------------------------
ref|NT_099026.1|MmY_98663_33_c      AT--GTCTGGAATTTTACAGAGGTGCTGAGAATACATGAACTCCACTGAG 10493

ref|NT_099035.1|MmY_98672_33_5      CCTTACAGCTAGAGATTCGAAAATTGGAGTTTTGCCAAG---GACATCTG 10556
ref|NT_078929.2|MmY_78994_33_4      CCTTACAGCTAGAGATTCGAAAATTGGAGTTTTGCCAAG---GACATCTG 10550
ref|NT_099035.1|MmY_98672_33_c      --------------------------------------------------
ref|NT_099026.1|MmY_98663_33_c      CCAACTTTCTAGCCCCCAGCAGGGTATTGTTTTTTGAAATCAATGATCAC 10543
```

```
ref|NT_099035.1|MmY_98672_33_5      GGCAGAGAAGAACATTCCTCCCGACTCTTCCCAACTCTCCAAACTCTCCT 10606
ref|NT_078929.2|MmY_78994_33_4      GGCAGAGAAGAACACTCCTCCCGACTCTTCCCAACTCTCCAAACTCTCCT 10600
ref|NT_099035.1|MmY_98672_33_c      --------------------------------------------------
ref|NT_099026.1|MmY_98663_33_c      ATCAGTAGAGAA-ATGAAAACCAACATGACCCTGATATTCTACCTTACCT 10592

ref|NT_099035.1|MmY_98672_33_5      CCCAACTCATCTCAATTCCTCAGGTAGCTAATAAAAACCCTCTTC-TGTC 10655
ref|NT_078929.2|MmY_78994_33_4      CCCAACTCATCTCAATTCCTCAGGTAGCTAATAAAAACCCTCTTC-TGTC 10649
ref|NT_099035.1|MmY_98672_33_c      --------------------------------------------------
ref|NT_099026.1|MmY_98663_33_c      CC-ACCTTACACCAATAAGTGAA-TGTTAAATATAAATTTAATTTATGTC 10640

ref|NT_099035.1|MmY_98672_33_5      ---ACATCTCAGAGTCATA------------------------------- 10671
ref|NT_078929.2|MmY_78994_33_4      ---ACATCTCAGAGTCATACGCCCCTGCCCTGCACAGTGGAAGGAGTTTG 10696
ref|NT_099035.1|MmY_98672_33_c      --------------------------------------------------
ref|NT_099026.1|MmY_98663_33_c      CAAATGAATCCAGATTACATTCTAAT------------------------ 10666

ref|NT_099035.1|MmY_98672_33_5      --------------------------------------------------
ref|NT_078929.2|MmY_78994_33_4      TCCTTAGCTAGCTGATAATAAAACCTCTTACAGTTTGCATCAGGTGTGGA 10746
ref|NT_099035.1|MmY_98672_33_c      --------------------------------------------------
ref|NT_099026.1|MmY_98663_33_c      --------------------------------------------------
```
