# Supplementary material for: Identification of novel Y chromosome encoded transcripts by testis transcriptome analysis of mice with deletions of the Y chromosome long arm
Source: Genome Biol. 2005 Dec 2;6(12):R102. doi: 10.1186/gb-2005-6-12-r102 (PMC1414076; doi:10.1186/gb-2005-6-12-r102)
Supplement: Additional data file 4 — Images showing Astx transcriptional analysis [file gb-2005-6-12-r102-S4.pdf]

Additional data file 4

In order to assess the spermatogenic cell types in which *Astx* is transcribed we carried out RT-PCR on testis RNA samples throughout the first spermatogenic wave using putatively *Astx*-specific primers, RNA FISH with an X chromosomal BAC containing the locus encoding the *Astx* cDNA AK076884, and RNA *in situ* with an *Astx* exon 4 probe. The RT-PCR (A) showed transcripts to be predominantly present from 22.5 dpp, consistent with spermatid expression, although there was also weak amplification at 14.5 and 19.5 dpp. Sequencing of the amplification products at 14.5 and 22.5 dpp confirmed that the transcripts amplified matched *Astx*. RNA FISH (B) on spread spermatogenic cells detected hybridizing X-encoded transcripts exclusively in spermatids. However, RNA *in situ* (C,D) detected strong hybridization in spermatogonia and primary spermatocytes, including pachytene stages in which the X is expected to be transcriptionally repressed, but no hybridization above background was detected in spermatids. Our interpretation of these apparently contradictory results is that the RT-PCR and RNA FISH techniques are detecting low level *Astx* transcripts that are not detectable above background by RNA *in situ*, while the latter technique is also detecting a related transcript that is expressed more strongly. Because this latter transcript is present in pachytene spermatocytes we suspect it may be autosomally encoded (and thus not subject to transcriptional repression during pachytene), but we have not been able to identify any related autosomal loci by BLAST with the two known *Astx* cDNAs.

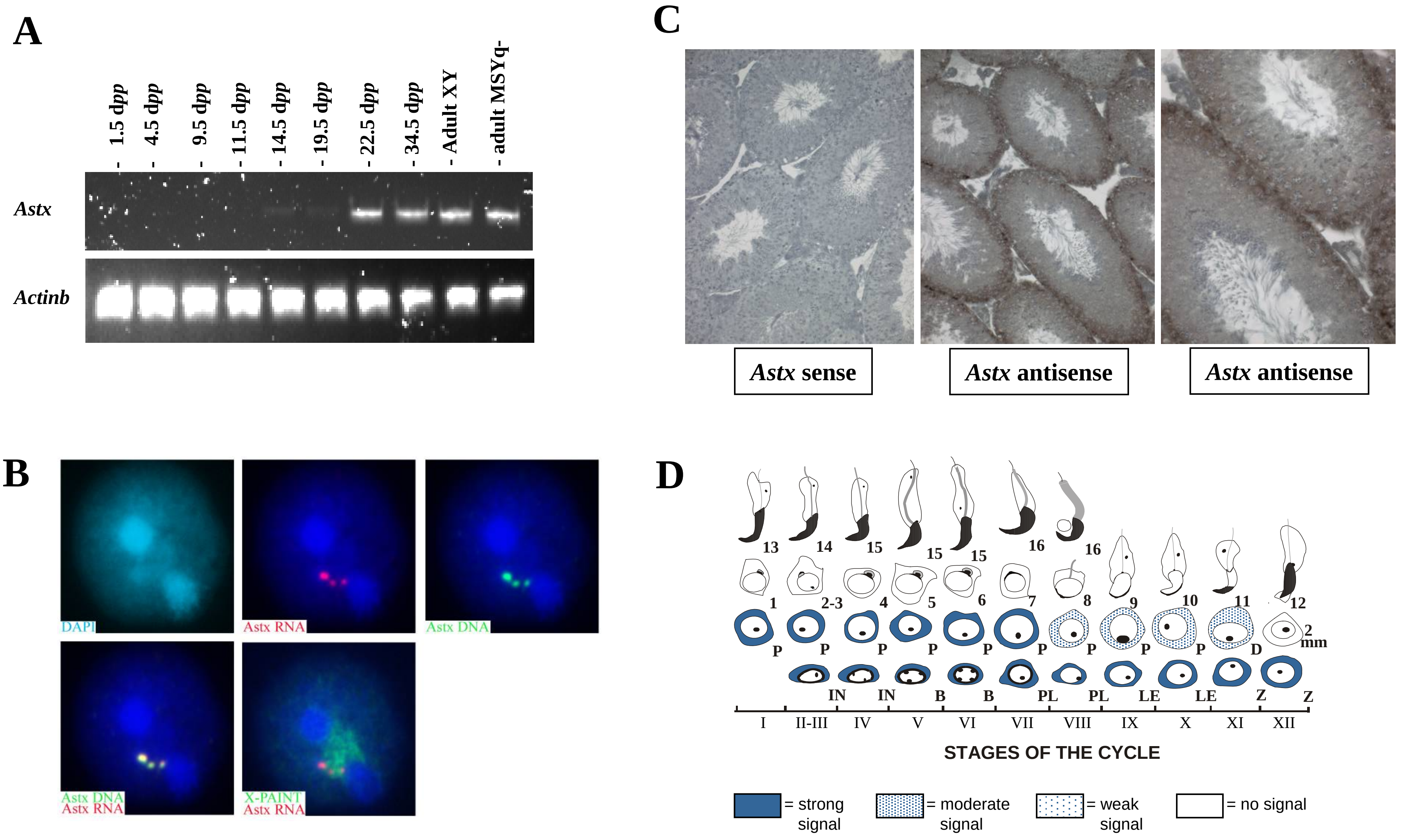
A
1.5 dpp
4.5 dpp
9.5 dpp
11.5 dpp
14.5 dpp
19.5 dpp
22.5 dpp
34.5 dpp
Adult XY
adult MSYq-
Astx
Actinb
B
DAPI
Astx RNA
Astx DNA
Astx DNA
Astx RNA
X-PAINT
Astx RNA
C
Astx sense
Astx antisense
Astx antisense
D
13 14 15 15 15 16 16
1 2-3 4 5 6 7 8 9 10 11 12
P P P P P P P P P D 2 mm
IN IN B B PL PL LE LE Z Z
I II-III IV V VI VII VIII IX X XI XII
STAGES OF THE CYCLE
= strong signal
= moderate signal
= weak signal
= no signal

Figure legend. Analysis of *Astx* transcription in the testis. (A) RT-PCR for testes from 1.5 dpp to adult together with adult MSYq- testes (that must lack MSYq-encoded *Asty* transcripts). The predominant expression is from 22.5 dpp after the appearance of spermatids. (B) RNA FISH on spread spermatogenic cells from adult testes. The only hybidization observed was to X-bearing spermatids (identified by their size, DAPI morphology and the fact that they are X paint positive but Y paint negative). Three RNA signals are seen that colocalise with the DNA FISH signals with the same BAC and are at the periphery of the X chromatin domain. Taken together with the RT-PCR results, we conclude that *Astx* is expressed in spermatids. (C,D) RNA *in situ* on adult XY testes (the same pattern is seen with adult MSYq- testes). Strong hybridization is apparent in spermatogoial and primary spermatocyte stages through to mid pachytene after which the signal attenualtes. No hybridization above background is seen in spermatids.
